# A Cobalt Mediated Nitrene Transfer *aza*-Wittig Cascade Reaction To Access 1,3,4-Oxadiazole Scaffolds

**DOI:** 10.1021/acs.orglett.3c00959

**Published:** 2023-05-24

**Authors:** Daniël S. Verdoorn, Prabhat Ranjan, Tim de Reuver, Elwin Janssen, Christophe M. L. Vande Velde, Jordy M. Saya, Bert U. W. Maes, Romano V. A. Orru

**Affiliations:** † Division of Organic Synthesis, Department of Chemistry, University of Antwerp, Groenenborgerlaan 171, B-2020 Antwerp, Belgium; ‡ Organic Chemistry, Aachen-Maastricht Institute for Biobased Materials (AMIBM), Maastricht University, Urmonderbaan 22, 6167RD Geleen, The Netherlands; § Department of Chemistry and Pharmaceutical Sciences and Amsterdam Institute for Molecular and Life Sciences (AIMMS), Vrije Universiteit Amsterdam, De Boelelaan 1108, 1081 HZ Amsterdam, The Netherlands; ∥ Intelligence in Processes, Advanced Catalysts and Solvents (iPRACS), Faculty of Applied Engineering, University of Antwerp, Groenenborgerlaan 171, B-2020 Antwerp, Belgium

## Abstract

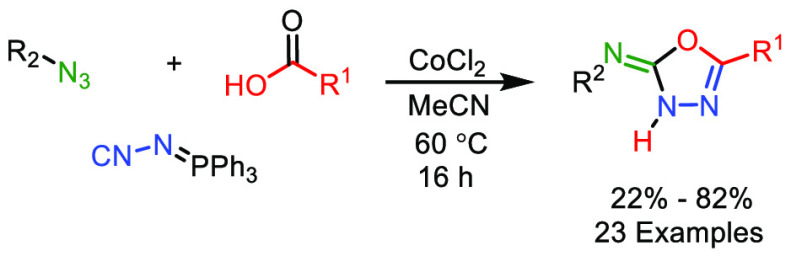

A cobalt(II) mediated three-component synthesis of 5-substituted-*N*-sulfonyl-1,3,4-oxadiazol-2(3*H*)-imines using sulfonyl azides, *N*-isocyaniminotriphenylphosphorane (NIITP), and carboxylic acids has been developed. This one-pot tandem reaction starts with a nitrene transfer to NIITP, followed by addition of the carboxylic acid to the *in situ* formed carbodiimide and subsequent intramolecular *aza*-Wittig reaction. Both the steric constraints of carboxylic acid and the stoichiometry of the employed cobalt salt determine the selectivity toward the two products, i.e. 5-substituted-*N*-sulfonyl-1,3,4-oxadiazol-2(3*H*)-imine versus 5-substituted-4-tosyl-2,4-dihydro-3*H*-1,2,4-triazol-3-one.

Multicomponent reactions (MCRs) have advanced as a reliable synthetic tool, especially in drug discovery and development. In these one-pot reactions, three or more reagents are combined to directly access complex and structurally diverse molecules.^1,2^ Although the potential of MCRs has long been recognized, the field developed rapidly in the past 20 years.^3^ At the heart of MCRs are the isocyanide based MCRs (IMCRs). Recently, utilizing transition metals to perform such IMCRs^4−6^ received considerable attention by the organic chemistry community, which greatly expanded the scope and applicability of IMCRs.

Transition metal (TM) catalyzed IMCRs involving (in)organic azides allow the formation of carbodiimides through nitrene transfers with isocyanides.^7^ This chemistry has been well established with palladium and rhodium. However, noble metals are expensive and scarce; therefore, moving toward base metals is essential from a sustainable chemistry perspective. Cobalt is an interesting candidate compared to other base metals, because it does not require complex ligands to promote the nitrene transfer, and it tolerates *in situ* functionalization of carbodiimides.^7−10^

In continuation of our interest to explore the reactivity of functionalized isocyanides in IMCRs, *N*-isocyaniminotriphenylphosphorane (NIITP) (**1**) attracted our attention, as it combines two functionalities in one reactant.

The synthesis of **1** was first reported in 1980^11^ by Weinberger and Felhammer; however, it was not until 2000 that Aller and Molina showed the usefulness of **1** in organic chemistry by accessing α-diazoketones (**5**) (Scheme 1 A).^12^ In their work, isocyanide **1** reacts with acid chlorides (**2**) to form intermediate **3**. Hydrolysis of **3**, and subsequent treatment with catalytic tosyl chloride, forms compound **5**.

**Scheme 1:**
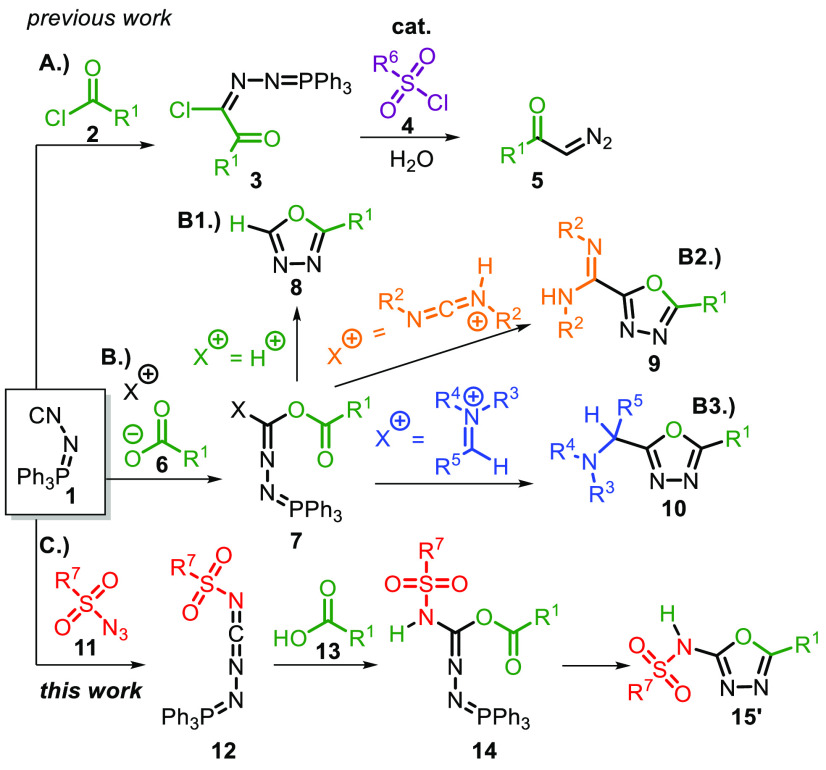
Synthetic Utilization of NIITP (**1**)

Later, the dual reactivity of **1** was recognized by Souldozi and Ramanzani,^13,14^ who demonstrated this in a reaction with benzoic acids to generate 1,3,4-oxadiazole **8** (Scheme 1**B1**). The authors rationalized that the reaction proceeds via protonation of **1**, followed by interception of the nitrilium ion by the carboxylate anion. The resulting *O*-acyl formimidate **7** subsequently undergoes an intramolecular *aza*-Wittig reaction. This chemistry was later extended by adding different electrophiles,^15−18^*i.e*. carbodiimidium and iminium, Scheme 1**B2** and **B3**, generating 1,3,4-oxadiazoles **9** and **10**, respectively. In addition, **1** is used toward the synthesis of other heterocycles such as 1,3,4-triazoles, or 1,3,4-thiodiazoles.^19,20^ Reports of **1** in transition metal chemistry are limited but known, with silver and molybdenum toward the synthesis of pyrazoles^21^ and unsymmetrical azines.^22^ Furthermore, **1** in combination with silver can also be employed as a safe cyanation reagent for terminal alkynes.^23^

For our work, the routes to acces 1,3,4-oxadiazoles are especially relevant. The five-membered heteroaromatic oxadiazole core contains two carbons, two nitrogens, and one oxygen atom, which exists in different regioisomeric forms. This motif is popular in many druglike molecules, and can be regarded as important amide (and ester) bioisosters.^24^ We envisioned a short and efficient route to these privileged scaffolds in medicinal chemistry^25^ using **1** in a base metal-catalyzed nitrene transfer, generating functionalized carbodiimide (**12**) *in situ*, which after attack of the carboxylic acid (**13**) forms intermediate **14**. The iminophosphorane functionality undergoes a subsequent *in situ aza*-Wittig reaction (Scheme 1**C**), providing 5-substituted-*N*-sulfonyl-1,3,4-oxadiazol-2-amines **15**′.

We commenced our studies by employing *para*-toluenesulfonyl azide (**11a**), **1**, and acetic acid (**13b**) as model reactants (Table 1). In the preliminary screening of the reaction conditions, CoCl_2_ (10 mol %) was employed in MeCN at 80 °C.

**Table 1:**
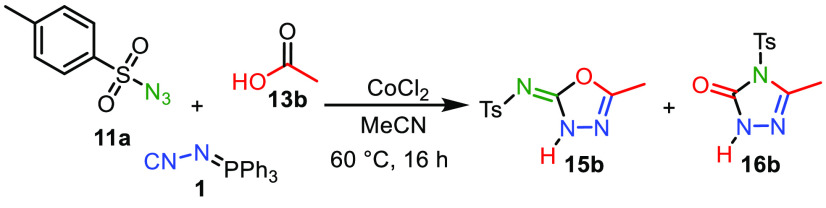
Reaction Optimization of the Three-Component Reaction^a^^,^^b^^,^^c^

Entry	Catalyst	Cat. mol %	*T* (°C)	Selectivity **15b**:**16b**	Yield (%)^b^^,^^c^**15b** + **16b** (**15b**)
**1**	**CoCl**_**2**_	10	**60**	**∼1:1**	**90**^c^**(50)**
2	CoBr_2_	10	60	∼1:1	78^c^ (42)
3	CoI_2_	10	60	∼1:1	51^c^ (30)
4	Co(OTf)_2_	10	60	∼1:1	65^c^ (39)
5	Co(acac)_3_	10	60		0
6	Pd(OAc)_2_	10	60		0
7	Pd(PPh_3_)_4_	10	60		0
8^d^	CoCl_2_	10	60	∼1:1	10 (5)
9	CoCl_2_	10	25	∼1:1	86^c^ (43)
10	CoCl_2_	10	80	∼1:1	90^c^ (50)
**11**	**CoCl**_**2**_	**100**	**60**	**>99:1**	**98 (98)**

a Standard conditions: **11a** (0.25 mmol, 1 equiv), **1** (1.2 equiv, **13b** (2 equiv), cat. (*X* mol %), in acetonitrile (10 mL, 25 mM), 60 °C, 16 h.

b ^1^H NMR yield using 2,5-dimethylfuran as internal standard.

c Combined yield of **15b** and **16b**, yield of **15b** between brackets.

d Concentration 0.1 M.

Initially, both **11a** and **1** were reacted; however, no formation of the desired carbodiimide intermediate **12a** could be detected via ^1^H NMR and LC-MS. Only when we included carboxylic acid **13b** at the start of the reaction, we could directly observe the desired 1,3,4-oxadiazole in 50% NMR yield (entry 1). Surprisingly, this compound occurred in its tautomeric 5-methyl-*N*-tosyl-1,3,4-oxadiazol-2(3*H*)-imine (**15b**) form instead of its **15b′** form (*vide infra*). It proved to be vital for the desired transformation to prestir the carboxylic acid with CoCl_2_. Additionally, we observed a constitutional isomer in 40% yield. Thorough NMR analysis revealed that this isomer is triazolone **16b** (for details see the Supporting Information). Acetonitrile proved to be the optimal solvent, as other solvents used in known TM catalyzed nitrene transfers, such as toluene,^26^ 1,4-dioxane,^27^ and tetrahydrofuran,^28^ showed no formation of **15b** and **16b** (Table S1). The use of polar protic solvents, such as isopropanol, or aprotic solvent, such as DMF, gave the products in similar selectivity, albeit in lower yields (Table S1). When varying cobalt sources, we did not observe any improvement in either yield or selectivity (Table S5). When varying the halogen counterion of the cobalt salt, we saw a declining trend in the overall yield (entries 1–3). However, their respective acids HX increase in acidity, in which **1** is prone to isomerization to the corresponding cyanamide.^29^ Switching to a pseudohalide, i.e. Co(OTf)_2_ (entry 4), showed a similar effect as the halides CoX_2_. When a cobalt(III), palladium(II), or palladium (0) source under otherwise similar conditions was used, no products were formed (entries 5–7), although conversion of **1** was observed.

Increasing the concentration from 0.025 M (10 mL) to 0.1 M (2.5 mL) drastically decreased the overall yield (from 90% to 10%) (entry 8). The temperature has little effect on the yield and selectivity of the reaction (entries 1, 9–10), with 60 °C as the optimal reaction temperature. When changing the amount of CoCl_2_ from catalytic to stoichiometric, we unexpectedly observed full selectivity to desired oxadiazol **15b** (entry 11).

All attempts to control the selectivity using catalytic amounts of CoCl_2_ in the presence of various additives (*i.e*., ligands, Lewis and Brønsted acids, Tables S3, S5) did not succeed.

Under the optimal conditions [**11a** (1 equiv), **13b** (2 equiv), **1** (1.2 equiv), CoCl_2_ (1 equiv), CH_3_CN (0.025 M), 60 °C, 16 h] we charted the scope and limitations of our three-component reaction. First, we examined the scope of the carboxylic acids (Scheme 2). Generally, the reaction allows a broad range of aliphatic carboxylic acids, giving the desired 1,3,4-oxadiazol-2(3*H*)-imines **15** (**a-j**) in 22–82% yield. The yields increase with the growing chain length of the primary carboxylic acids (**15a**–**15c**; 40–64%). Although, in certain cases the use of secondary- and tertiary-branched acids results in higher yields (**15f**; 71% and **15g**; 80%), no general trend is observed. Other secondary- and tertiary-branched acids gave similar [**15d** (46%), **15e** (60%), **15h** (50%), **15i** (48%)], or lower [**15j** (22%)], yields. Noteworthily, products **15f**, **15i**, and **15j** can be synthesized selectively with 10 mol % CoCl_2_, generating target products **15** in comparable yield. This effect indicates that the selectivity toward oxadiazoles **15** may result from steric congestion close to the carboxylic acid moiety. Cobalt complexes are well-known to act as Lewis acids,^8^ which in turn can provide a steric effect, similar to a carboxylic acid. This is supported by the linear increase in selectivity for **15** versus **16**, with the mol % CoCl_2_ loading used (Table S6).

**Scheme 2:**
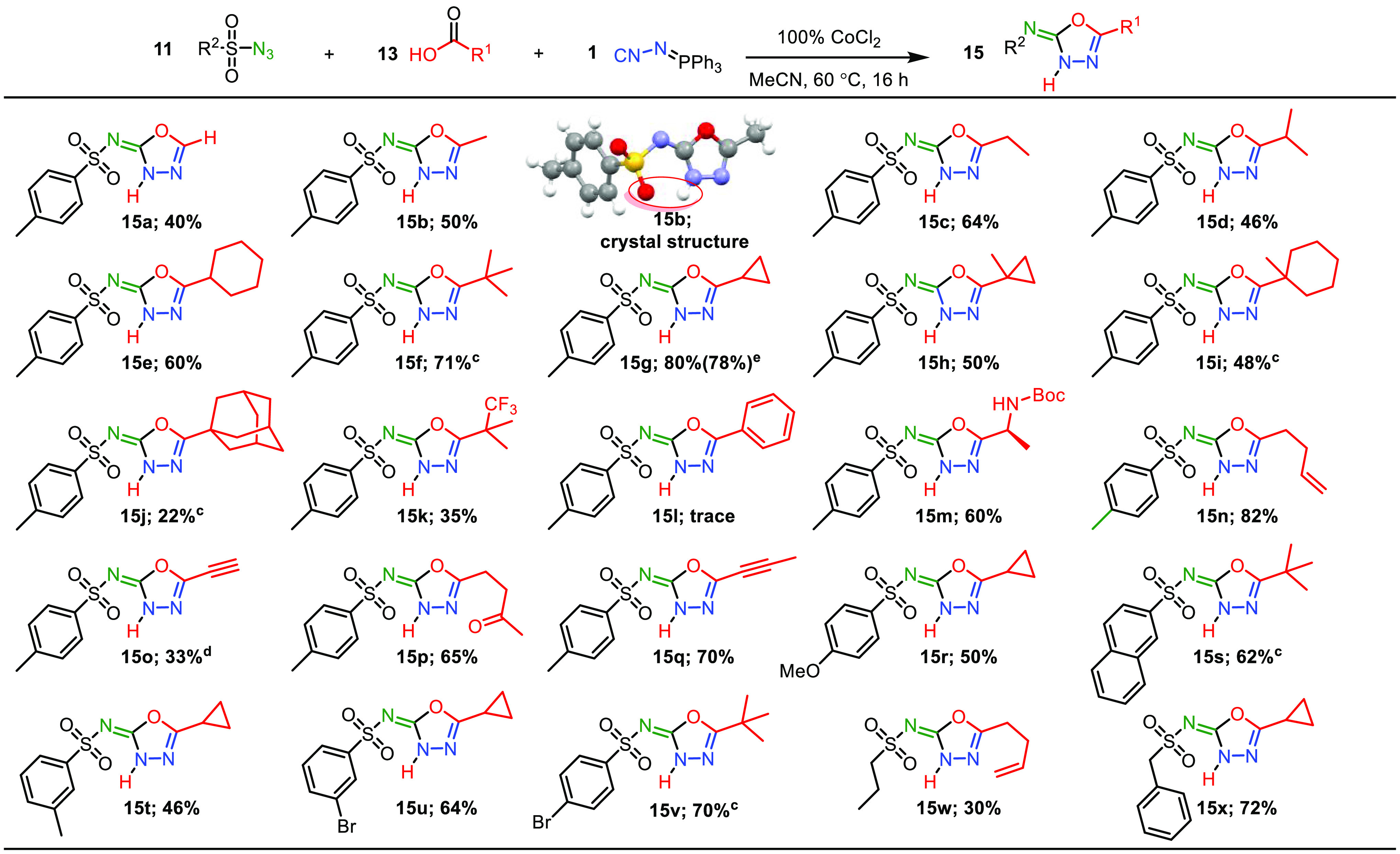
Reaction Scope of the Three-Component Reaction^,^ Reaction conditions: **11** (0.5 mmol), **1** (0.6 mmol), **13** (1 mmol), CoCl_2_ (100%), CH_3_CN(25 mM), N_2_ atmosphere, 60 °C, 16 h. Isolated yields. 10 mol % CoCl_2_ used. TMS protected propargylic acid used. 1 mmol scale.

Most of the other used carboxylic acids followed a similar trend, when these catalytic CoCl_2_ conditions were employed (Scheme S1). The only notable exception being 1-methyl-cyclopropylic acid, which favored formation of triazolone **16h** in a 3:1 ratio with 10 mol % CoCl_2_. Fortunately, when stoichiometric CoCl_2_ is used, full selectivity to oxadiazole **15h** is reached.

Additionally, our three-component reaction tolerates a variety of functional groups in the carboxylic acid. Functionalities such as a double bond (**15n**), triple bond (**15o**, **15q**), ketone (**15p**), and trifluoromethyl (**15k**) are accepted in moderate to good yield (33–82%).

Furthermore, *N-*protected amino acids can be used (**15m**), which after deprotection can liberate an amine moiety. These tolerated functional groups are synthetic handles which can be utilized to create additional diversity and complexity. The process is sensitive to the p*K*_a_ of the carboxylic acid. TFA and benzoic acid were not accepted in the transformation.

The structure of **15b** was unambiguously confirmed via X-ray crystallography (CCDC 2225280). The crystal structure showed that the 1,3,4-oxadiazole formed the 1,3,4-oxadiazol-2(3*H*)-imine tautomer through a hydrogen bond between N3–H and S=O (Scheme 2, **15b** crystal structure).

Next, we investigated the azide scope (Scheme 2). Both arene- and alkanesulfonyl azides are accepted well in the reaction, in 30–72% yield (**15r**–**x**). Benzenesulfonyl with an electron donating 4-methoxy group (**15r**) and 3-methyl (**15t**) proved less effective compared to 4-methyl (**15g**). A 4-bromobenzenesulfonyl azide (**15v**) gave intermediate (70%) results. Polycyclic aromatic hydrocarbons are also compatible, as exemplified by naphthalensulfonyl derivative **15s**. Aliphatic alkanesulfonyl azides, such as propane (**15w**) and phenylmethane (**15x**), also provided the target compound. Other classes of azides did not provide the title compound **15** (Scheme S2).

To demonstrate the synthetic potential of our developed method, we installed 1,3,4-oxadiaziol-2(3*H*)-imine on dehydrocholic acid, generating **15y** in 42% yield (Scheme 3). Moreover, deprotection of the tosyl group of **15g** generates 1,3,4-oxazol-2-amine (**17g**) in 72% yield. Heterocycle **17g** is a precursor to the antibacterial compound **18**.^30^

**Scheme 3:**
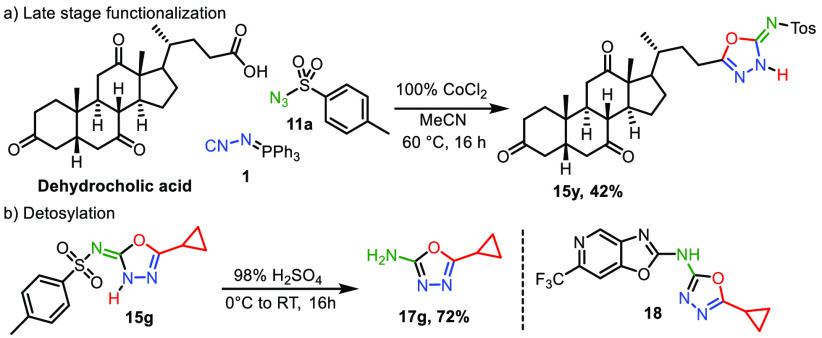
Application of the Method on Dehydrocholic Acid (a) and *N*-Ts Deprotection of the *N*-Tosyl-1,3,4-oxadiaziol-2(3*H*)-imines (b)

Our proposed mechanism for the formation of products **15** and **16** is depicted in Scheme 4. The possible mechanisms of the formation of carbodiimide **12** are described and analyzed in detail in our review.^7^ Both products are formed from the *in situ* generated carbodiimide **12**. We provide a mechanistic rationale for both, the formation of the desired 1,3,4-oxadiazol-2(3*H*)-imine product **15** (route **a**), and the undesired 1,2,4-triazol-3-one product **16** (route **b**).

**Scheme 4:**
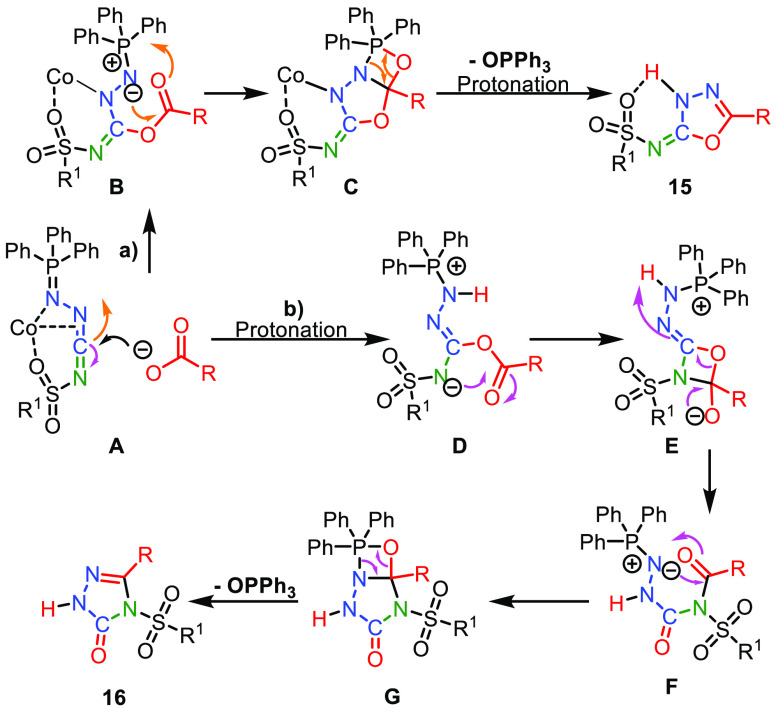
Proposed Reaction Mechanism towards **15** and **16**

Formation of **15** is initiated by an attack of the carboxylate onto carbodiimide **A**, providing intermediate **B** (route **a**). The iminophosphorane moiety of **B** subsequently reacts with its ester carbonyl via an *aza*-Wittig reaction, involving the formation of the imine double bond of the desired product **15**. In accordance to the proposed mechanism, we speculate that pathway B is enhanced as it can stabilize the ylide, before the aza-Wittig reaction.

Concomitantly, triphenylphosphine oxide is released. Formation of product **16** also starts with addition of the carboxylate onto the protonated carbodiimide **A**, providing intermediate **D** (route **b**). *N*,*O*-Acyl transfer through the four-membered intermediate **E** leads to *N*-acylurea **F**. The iminophosphorane moiety and the amide carbonyl of **F** subsequently undergo an *aza*-Wittig reaction, releasing product **16** and triphenylphosphine oxide byproduct. Interestingly, this *aza*-Wittig reaction in **F** is faster than the potentially competing isocyanate elimination.^31^ We investigated the possibility of isomerization of **15** into **16**; however, when **15** is subjected to the general reaction conditions, no isomerization occurred. Further studies into the role of CoCl_2_ in the selectivity of this phenomenon are being done in our lab.

In conclusion, we developed a cobalt(II) mediated/catalyzed synthesis of 5-substituted-*N*-sulfonyl-1,3,4-oxadiazol-2(3*H*)-imines (**15**), using a three-component reaction of sulfonyl azides (**11**), NIITP (**1**), and carboxylic acids (**13**). Selective formation of 5-substituted-*N*-sulfonyl-1,3,4-oxadiazol-2(3*H*)-imines (**15**) in reasonable to good yields is achieved by employing a sterically hindered carboxylic acid and catalytic CoCl_2_, or a nonsterically hindered carboxylic acid and stoichiometric CoCl_2_. The *N*-sulfonyl functionality in **15** can be deprotected providing 1,3,4-oxazol-2-amines, which allows post-functionalization transformations.

## Data Availability

The data underlying this study are available in the published article and its Supporting Information.

## Supporting Information Available

Detailed experimental procedures, crystallographic data for **15b**, detailed characterization of **16a** and **16b**, detailed spectroscopic data for all new compounds, and copies of ^1^H and ^13^C NMR spectra (PDF)
