# Supplementary material for: A Cobalt Mediated Nitrene Transfer aza-Wittig Cascade Reaction To Access 1,3,4-Oxadiazole Scaffolds
Source: Org Lett. 2023 May 24;25(22):4005–9. doi: 10.1021/acs.orglett.3c00959 (PMC10262268; doi:10.1021/acs.orglett.3c00959)

Supporting information

A Cobalt Mediated Nitrene Transfer *aza*-Wittig Cascade Reaction to access 1,3,4-Oxadiazole Scaffolds

Daniël S. Verdoorn[a,b], Prabhat Ranjan[b], Tim de Reuver[b], Elwin Janssen[c], Christophe M.L. Vande Velde[d], Jordy M. Saya[b]*, Bert U.W. Maes[a]*, Romano V.A. Orru[b]*

a. Division of Organic Synthesis, Department of Chemistry, University of Antwerp, Groenenborgerlaan 171, B-2020 Antwerp, Belgium. b. Organic Chemistry, Aachen-Maastricht Institute for Biobased Materials (AMIBM), Maastricht University, Urmonderbaan 22, 6167RD Geleen, the Netherlands. c. Department of Chemistry and Pharmaceutical Sciences and Amsterdam Institute for Molecular and Life Sciences (AIMMS), Vrije Universiteit Amsterdam, De Boelelaan 1108, 1081 HZ Amsterdam, The Netherlands. d. Intelligence in Processes, Advanced Catalysts and Solvents (iPRACS), Faculty of Applied Engineering, University of Antwerp, Groenenborgerlaan 171, B-2020 Antwerp, Belgium.

*E-mail: j.saya@maastrichtuniversity.nl
*E-mail: bert.maes@uantwerpen.be
*E-mail: r.orru@maastrichtuniversity.nl

Contents

Supporting information S1
1.1 General information S6
2.1 Optimization studies S7
Figure S1: S7
Table S1: Solvent effect[a,b] S8
Table S2: Temperature effect [a,b] S9
Table S3: Additive effect [a,b] S10
Table S4: Stoichiometry effect[a,b] S11
Table S5: Catalyst screening [a,b] S12
Table S6: Catalyst loading effect[a,b] S13
Figure S2: S13
2.2 Effect of Electronics and Sterics of Carboxylic Acid on the Selectivity. S14
Scheme S1: Steric and electronic effect of carboxylic acids on selectivity [a,b] S14
2.3 Unsuccessful results S15
Scheme S2: Unsuccessful results[a] S15
2.4 Methods S16
Synthesis of sulfonyl azides (GP-A)[1]: S16
Synthesis of acyl azides (GP-B)[2]: S16
General procedure synthesis of oxadiazoles 100% Co(II) loading (GP-C): S16
General procedure synthesis of oxadiazoles 10% Co(II) loading (GP-D): S17
Detailed procedure for 1 mmol scale: S17
General procedure for detosylation, generation of free amine (GP-E)[3]: S18
Special remarks regarding methodology: S18
2.5 Characterization side product 16 .... S19
2.6 X-ray analysis S29
Figure S3: S29
2.7 References S30
3.1 Spectral data S31
(15a): S31
(15b): S31
(15c): S32
(15d): S32

(15e): S33
(15f): S33
(15g): S34
(15h): S34
(15i): S35
(15j): S35
(15k): S36
(15m): S36
(15n): S37
(15o): S37
(15p): S38
(15q): S38
(15r): S39
(15s): S39
(15t): S40
(15u): S40
(15v): S41
(15w): S41
(15x): S42
(15y): S42
(17g): S43
^{1}H spectrum 15a (300 MHz, $CDCl_{3,}$ DMSO) S44
^{13}C spectrum 15a (75 MHz, CDCl3, DMSO) S45
^{1}H spectrum 15b (300 MHz, $CDCl_3$) S46
^{13}C spectrum 15b (75 MHz, $CDCl_3$) S47
^{1}H spectrum 15c (300 MHz, $CDCl_3$) S48
^{13}C spectrum 15c (75 MHz, $CDCl_3$) S49
^{1}H spectrum 15d (300 MHz, CDCl3) S50
^{13}C spectrum 15d (75 MHz, $CDCl_3$) S51
^{1}H spectrum 15e (300 MHz, $CDCl_3$) S52
^{13}C spectrum 15e (75 MHz, $CDCl_3$) S53
^{1}H spectrum 15f (300 MHz, $CDCl_3$) S54
^{13}C spectrum 15f (75 MHz, $CDCl_3$) S55

^{1}H spectrum 15g (300 MHz, $CDCl_3$) S56
^{13}C spectrum 15g (75 MHz, $CDCl_3$) S57
^{1}H spectrum 15h (300 MHz, $CDCl_3$) S58
^{13}C spectrum 15h (75 MHz, $CDCl_3$) S59
^{1}H spectrum 15i (300 MHz, $CDCl_3$) S60
^{13}C spectrum 15i (75 MHz, $CDCl_3$) S61
^{1}H spectrum 15j (300 MHz, $CDCl_3$) S62
^{13}C spectrum 15j (75 MHz, $CDCl_3$) S63
^{1}H spectrum 15k (300 MHz, $CDCl_3$, DMSO) S64
^{19}F spectrum 15k (282 MHz, $CDCl_3$, DMSO) S65
^{13}C spectrum 15k (75 MHz, Acetone-D_6) S66
^{1}H spectrum 15m (300 MHz, $CDCl_3$) S67
^{13}C spectrum 15m (75 MHz, $CDCl_3$) S68
^{1}H spectrum 15n (300 MHz, $CDCl_3$) S69
^{13}C spectrum 15n (75 MHz, $CDCl_3$) S70
^{1}H spectrum 15o (300 MHz, $CDCl_3$) S71
^{13}C spectrum 15o (75 MHz, $CDCl_3$) S72
^{1}H spectrum 15p (300 MHz, $CDCl_3$) S73
^{13}C spectrum 15p (75 MHz, $CDCl_3$) S74
^{1}H spectrum 15q (300 MHz, $CDCl_3$) S75
^{13}C spectrum 15q (75 MHz, $CDCl_3$) S76
^{1}H spectrum 15r (300 MHz, $CDCl_3$) S77
^{13}C spectrum 15r (75 MHz, $CDCl_3$) S78
^{1}H spectrum 15s (300 MHz, $CDCl_3$) S79
^{13}C spectrum 15s (75 MHz, $CDCl_3$) S80
^{1}H spectrum 15t (300 MHz, $CDCl_3$) S81
^{13}C spectrum 15t (75 MHz, $CDCl_3$) S82
^{1}H spectrum 15u (300 MHz, $CDCl_3$) S83
^{13}C spectrum 15u (75 MHz, $CDCl_3$) S84
^{1}H spectrum 15v (300 MHz, $CDCl_3$) S85
^{13}C spectrum 15v (75 MHz, $CDCl_3$) S86
^{1}H spectrum 15w (300 MHz, $CDCl_3$) S87
^{13}C spectrum 15w (75 MHz, $CDCl_3$) S88

^{1}H spectrum 15x (300 MHz, $CDCl_3$) S89

^{13}C spectrum 15x (75 MHz, $CDCl_3$) S90

^{1}H spectrum 15y (300 MHz, $CDCl_3$) S91

^{13}C spectrum 15y (75 MHz, $CDCl_3$) S92

1.1 General information

Commercially available reagents were purchased from Sigma-Aldrich, Fischer Scientific, Strem Chemicals, TCI Chemicals, Activate Scientific, or Fluorochem and were used as purchased unless mentioned otherwise. Isocyanide **1** (TCI Chemicals), and all acids (Sigma-Aldrich) were commercially available. *p*-toluene sulfonyl azide (Sigma-Aldrich), is commercially available, other azide were synthesized and are known compounds, see reference general procedure). Solvents were purchased from VWR Chemicals or Sigma-Aldrich and used without purification, unless stated otherwise. Reagent grade, anhydrous solvent was used for the optimization. Thin layer chromatography (TLC) was performed using plates from Merck (SiO_2, Kieselgel 60 F254 neutral, on aluminium with fluorescence indicator) and compounds were visualized by UV detection (254 nm), $KMnO_4$, and/or hanessian's, stain. Manual flash column chromatography was performed by employing silica (200-300 mesh) as support and *n*-heptane/ethyl acetate as eluent. NMR spectra were recorded on a Brüker Avance 300 using the residual $CDCl_3$ as internal reference (^{1}H: δ 7.26 ppm, ^{13}C: δ 77.16 ppm). Chemical shifts (δ) are given in ppm and coupling constants (J) are quoted in hertz (Hz). Resonances are described as s (singlet), d (doublet), t (triplet), q (quartet), br (broad singlet), and m (multiplet) or combinations thereof. Structural assignments were made with additional information from gCOSY, gHSQC, and gHMBC experiments. Electrospray ionization (ESI) high-resolution mass spectrometry was carried out using a Waters Synapt G2 (Q-TOF) in positive ion mode.
X-ray diffraction data were collected at room temperature with Cu Kα radiation via ω-scans on a Rigaku Rapid-S diffractometer, using CrystalClear 2.1.[1] Data reduction was done with HKL3000[2], and the structure was solved with SHELXS 2013/1.[3,4] After structure solution, the atoms were modeled with calculated non-spherical structure factors[5] from the DiSCaMB library[6] according to the TAAM method[7,8] as implemented via NoSpherA2 in Olex2.[9,10,11,12] This allows for meaningful refinement of hydrogen distances and thermal parameters, and a closer fit of the observed electron density to the proposed structure model. NMR Data were processed with Mestre nova version 12. Crystals suitable for single crystal X-ray diffraction were grown by slow evaporation of DCM.

2.1 Optimization studies

General procedure for optimization

A flame-dried schlenk vial was charged with cobalt chloride (and carboxylic acid **if solid**) under a continuous nitrogen flow (Figure S1 a). Subsequentyl, dry MeCN was added and after the catalyst was completely dissolved the solution was stirred for 10 minutes, before **liquid** carboxylic acid **13a** was added and the mixture was again stirred for 10 minutes (blue solution, figure S1 b). Then, N-isocyanoiminotriphenylphosphorane (**1**) was added in one portion and the flask was rinsed with additional dried MeCN under continuous nitrogen flow. The mixture was stirred until the isocyanide was completely dissolved (25 mM, brown solution, **if** catalytic cobalt chloride; Blue/Green solution **if** stoichiometric cobalt chloride, Figure S1 c). *p*-toluenesulfonyl azide (**11a**) was added to the solution under continuous nitrogen flow. The mixture was heated to 60 °C and stirred for 16 h (Green solution). The reaction was concentrated *in vacuo* and re-dissolved in DCM. The organic layer was washed with HCl (1 M), subsequently, the organic layer was extracted three times with NaOH (1 M) and the organic layer is discarded. The aqueous layer was acidified with HCl (12 M) until pH 1 and was extracted three times with DCM. The combined organic layers were dried over $MgSO_4$, filtered, and concentrated *in vacuo*. All yields for the optimization were determined by using 2,5-dimethylfuran (0.25 mmol) as internal standard.

Figure S1: a) Flask charged with cobalt chloride (and acid, in case **solid**); b) Cobalt chloride dissolved in 5 mL MeCN; c) isocyanide added and dissolved in an additional 5 mL MeCN. All under continuous N_2 flow.

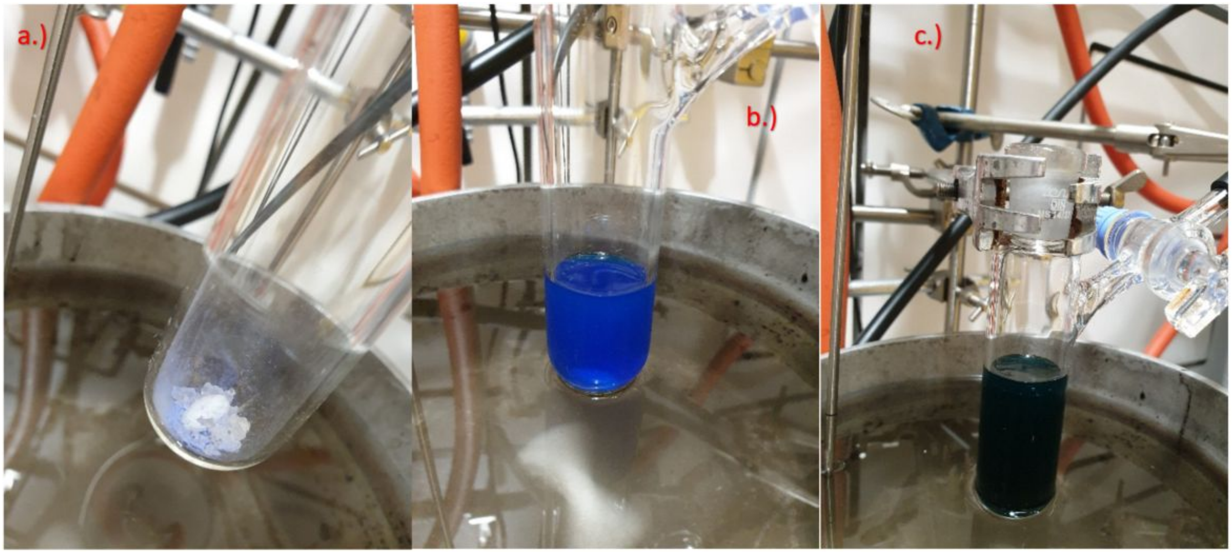

Remark on the general method. If the acid is solid, it was added simultaneously with the cobalt chloride. If the acid was liquid, it was added after the cobalt catalyst was dissoleved and had stirred for 10 minutes.

Table S1: **Solvent effect**[a,b]

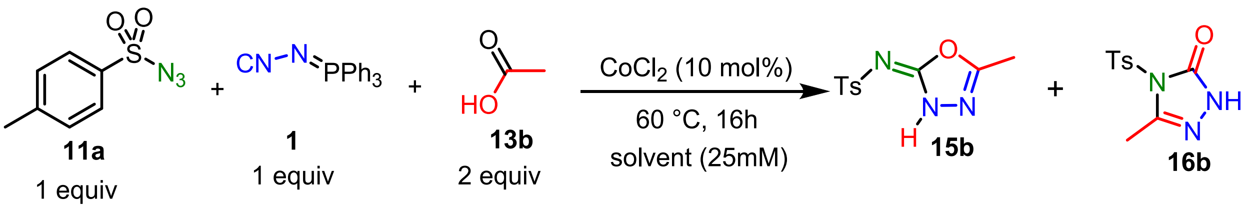

Entry	Solvent	Yield (%) **15b**	Yield (%) **16b**
**1**	**MeCN**	**50**	**40**
2	THF	-	18
3	DCE	6	6
4	DMF	6	29
5	Toluene	-	-
6	Dioxane	-	-
7	EtOAc	-	-
8	$CHCl_3$	3	3
9	HFIP	-	-
**10**	**Isopropanol**	**43**	**43**
11	Acetone	9	26
12	Pyridine	-	-
13	MeCN:HFIP (8:2)	32	-
14	MeCN:Toulene (1:1)	42	37
15	MeCN:Acetone (1:1)	42	30
16	MeCN:THF (1:1)	25	9
17	MeCN:HFIP (1:1)	14	-
18	MeCN:HFIP (99:1)	44	39

[a]Reaction condition: 0.25 mmol **11a**, 0.25 mmol **1**, 0.5 mmol **13b**, 0.025 mmol $CoCl_2$, 10 mL solvent (25 mM), 60 °C, 16 h . [b]Yields were determined by ^{1}H NMR using 2,5 dimethylfuran as internal standard.

Key observation: Polar aprotic and polar protic solvents (entry 1 and 10) prove to be effective in catalyzing the reaction with excellent mass balance, albeit without selectivity.

Table S2: **Temperature effect** [a,b]

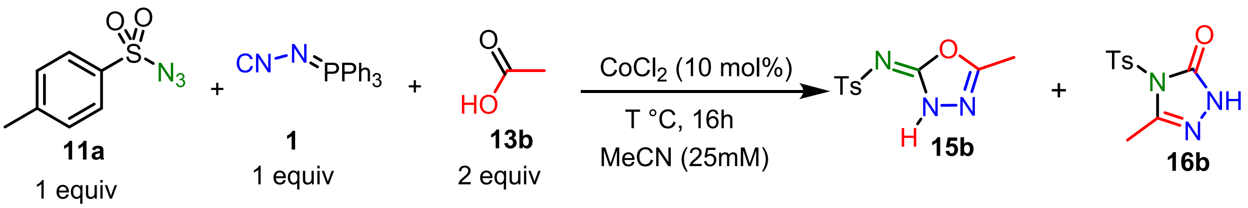

Entry	Temperature (^{o}C)	Yield (%) **15b**	Yield (%) **16b**
1	RT	43	43
2	40	40	47
3	60	50	40
4	80	50	40
5	-18 to RT	36	33
6	-18	27	28

[a]Reaction condition: 0.25 mmol **11a**, 0.25 mmol **1**, 0.5 mmol **13b**, 0.025 mmol $CoCl_2$, 10 mL MeCN (25 mM), T °C, 16 h. [b]Yields were determined by ^{1}H NMR using 2,5 dimethylfuran as internal standard.

Key observation: Decreasing or increasing the temperature did not provide a better selectivity. Interestingly, the reaction proceeds at temperatures as low as -18 °C, however with a lower conversion.

Table S3: **Additive effect** [a,b]

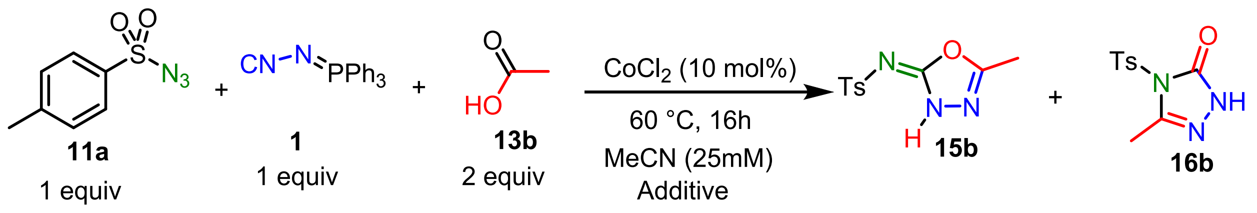

Entry	Additive	Yield (%) **15b**	Yield (%) **16b**
1	TFA.DMAP (2.2 equiv)	21	9
2	DMAP (1.2 equiv)	17	-
3	DMAP (2.2 equiv)	21	-
4	DMAP (4 equiv)	9	-
5	K_3PO_4 (3 equiv)	17	-
6	K_2CO_3 (3 equiv)	16	-
7	$FeCl_3$ (1 equiv)	26	-
8	Ag(OTf) (1 equiv)	-	-
9	LiCl (1 equiv)	8	-
10	$AgNO_3$ (0.1 equiv)	-	-
11	$AgNO_3$ (0.3 equiv)	-	-
12	Sc(III)OTf (1 equiv)	-	-
13	Zn(OTf) (1 equiv)	-	-
14	$B(OH)_3$ (1 equiv)	23	23

[a]Reaction condition: 0.25 mmol **11a**, 0.25 mmol **1**, 0.5 mmol **13b**, 0.025 mmol $CoCl_2$, additive, 10 mL MeCN (25 mM), 60 °C, 16 h. [b]Yields were determined by ^1H NMR using 2,5 dimethylfuran as internal standard.

Key observation: To promote the O, N-acyl transfer various concentrations of DMAP or the TFA salt of DMAP were added. As can be observed from entry 1 to 4, no beneficial results were observed. Other additives such as bases (entry 5 and 6) and lewis acids (7-14) were tried, however, this did not provide positive results.

Table S4: **Stoichiometry effect**[a,b]

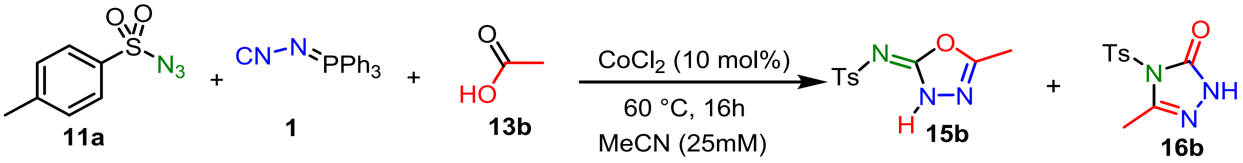

Entry	AcOH **13b**	NIITP **1**	Tosylazide **11a**	Yield (%) **15b**	Yield (%) **16b**
**1**	**2 equiv**	**1.2 equiv**	**1 equiv**	**50**	**40**
2	2 equiv	1.5 equiv	1 equiv	47	45
3	2 equiv	2 equiv	1 equiv	25	23
4	2 equiv	1 equiv	1.2 equiv	46	39
5	2 equiv	1 equiv	1.5 equiv	45	45
6	2 equiv	1 equiv	2 equiv	47	48
7	1 equiv	1 equiv	1 equiv	41	41
8	1.2 equiv	1 equiv	1 equiv	42	40
9	1.5 equiv	1 equiv	1 equiv	39	42
10	3 equiv	1 equiv	1 equiv	44	35

[a]Reaction condition: X mmol **11a**, Y mmol **1**, Z mmol **13b**, 0.025 mmol $CoCl_2$, MeCN (10 mL), 60 °C, 16 h. [b]Yields were determined by ^{1}H NMR using 2,5 dimethylfuran as internal standard.

Key observation: From the stoichiometry evaluation it became clear that 2 equiv. AcOH (**13b**), 1.2 equiv. NIITP (**1**) and 1 equiv. tosylazide (**11a**) proved optimal, in achieving the highest yield of **15b**.

Table S5: **Catalyst screening** [a,b]

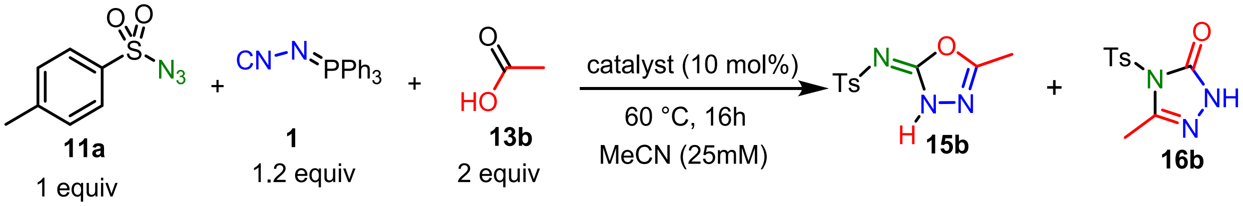

Entry	Catalyst	Ligand (no or 20 mol%)	Yield (%) **15b**	Yield (%) **16b**
1	$CoCl_2$		50	40
2	CoF_2		-	-
3	$CoBr_2$		42	36
4	CoI_2		30	21
5	$Co(OTf)_2$		39	26
6	$Co(acac)_3$		-	-
7	$Co(C_2O_4)_2$		-	-
8	$Co(OAc)_2$		23	37
9	$Pd(PPh_3)_4$		-	-
10	PdI_2		-	-
11	$Pd(OAc)_2$		-	-
12	$PdCl_2$		-	-
13	$Pd(PPh_3)_4$		-	-
14	PdI_2		-	-
15	$Pd(OAc)_2$		-	-
16	$PdCl_2$		-	-
17	$Pd(OAc)_2$	Xantphos	-	-
18	$CoCl_2$	Pivalic acid	43	43
19	$Fe(CO)_3(NO)TBA$	-	-	-
20	$CoCl_2$	dppp	-	-
21	$CoBr_2$	dppp	-	-
22	$CoCl_2$	4,4-di-tert-butyl-2-2-bipyridine	-	-

[a]Reaction condition: 0.25 mmol **11a**, 0.3 mmol **1**, 0.5 mmol **13b**, 0.025 mmol catalyst, MeCN (10 mL), 60 °C, 16 h.
[b]Yields were determined by ^1H NMR using 2,5 dimethylfuran as internal standard.

Key observation: Entry 1 shows the standard conditions with cobalt (II) chloride. from which improvement was sought. Different cobalt (II) and cobalt (III) catalysts were screened (entry 2-8). Cobalt (II) bromide and cobalt (II) iodide (entry 3 and 4), and cobalt (II) triflate and cobalt (II) acetate (entry 5 and 8) show reactivity in the transformation, albeit without improvement compared to entry 1. Cobalt (III) acetylacetonate (entry 6) and cobalt (II) oxalate (entry 7) show no performance in the reaction. Different palladium sources were tried in the reaction (entry 9 to 12). However, both palladium (0) and palladium (II) species showed no reactivity in neither acetonitrile nor THF (entry 12 to 15) as solvent. Even with xantphos as ligand (entry 17) no reactivity was observed. Entry 18 shows pivalic acid as ligand in the

standard conditions however, no clear selectivity was achieved. Other phosphor and nitrogen based ligands were used, however without success (entry 20-22).

Table S6: **Catalyst loading effect**[a,b]

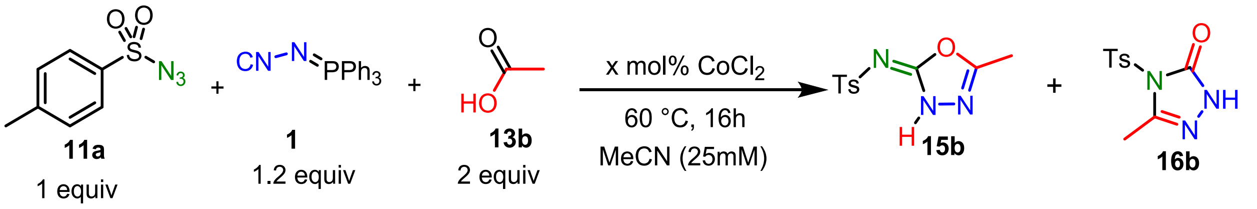

Entry	$CoCl_2$ (mol%)	Yield (%) **15b**	Yield (%) **16b**	Selectivity **15b** : **16b**
1	0	-	-	-
2	5	50	40	~ 1:1
3	10	50	40	~ 1:1
4	30	62	28	~ 3:1
5	50	81	17	~ 4:1
6	70	90	10	~ 9:1
7	100	98	0	> 99:1

[a]Reaction condition: 0.25 mmol **11a**, 0.3 mmol **1**, 0.5 mmol **13b**, x mol% $CoCl_2$ MeCN (10 mL), 60 °C, 16 h. [b]Yields were determined by ^{1}H NMR using 2,5 dimethylfuran as internal standard.

Key observation: To our surprise the increase of $CoCl_2$ loading increased the selectivity of the reaction. The yield achieved by the transformation was approximately equimolar for **15b** and **16b** under standard reaction conditions (entry 3). When 1 equiv. (100 mol%) catalyst cobalt chloride was used, only product **15b** was observed in 98% yield. The yield varied linear with the $CoCl_2$ loading between 10 mol% and 100 mol %.

Figure S2: Catalyst loading effect

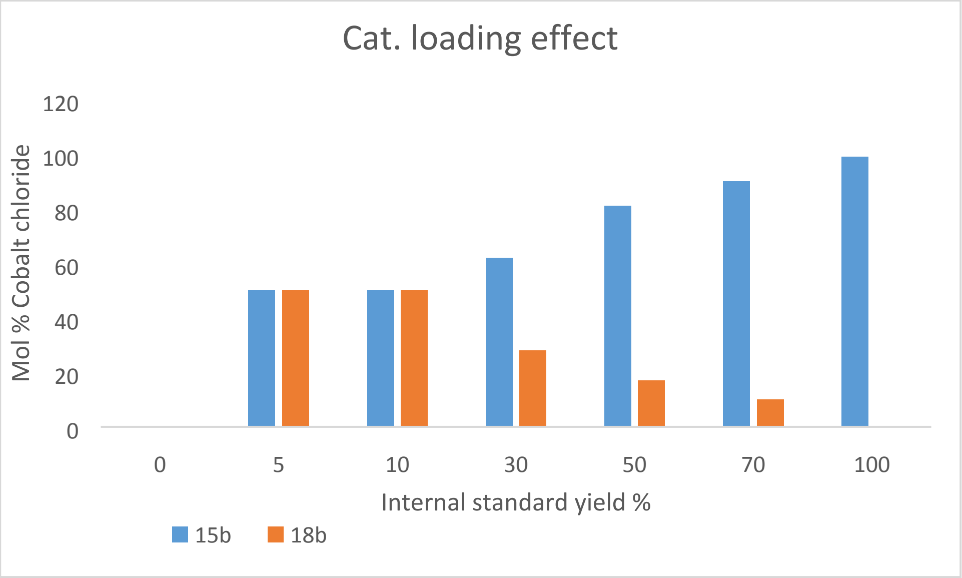

2.2 Effect of Electronics and Sterics of Carboxylic Acid on the Selectivity.

We started the investigation by employing different substituted carboxylic acids. A clear trend can be observed when increasing the steric congestion on the alpha carbon of the carboxylic acid. Looking at **15c**, **15e**, **15f**, and **15i**, we see an increase in selectivity towards the desired product **15** versus the corresponding compounds **16**. Both **15f** and **15i** can even be achieved selectively with a catalytic amount of $CoCl_2$, proving that steric hindrance is the key factor to control selectivity. When no sterically hindered carboxylic acid is used, 100% $CoCl_2$ provides the required steric hindrance via ligation to the carbodiimide intermediate **12** in a one-to-one ratio. Interestingly, as a sole exception, **15h** is the minor compound and **16h** the major compound. This steric effect is in compliance with the proposed mechanism of the side product formation, preventing the N,O-acyl transfer. Furthermore, our results also prove that the nitrene transfer to isocyanide can be achieved catalytically, however, to achieve selectivity towards the desired product **15**, a stoichiometric amount of cobalt needs to be used when acids do not provide the necessary steric hinderance. Lastly, **15y** and **15z** did not perform in the reaction, indicating that when an acid with a pKa < 3 is used, the nitrene transfer is inhibited. In literature isocyanides are also known to be prone to deactivation when strong acidic conditions are used.

Scheme S1: Steric and electronic effect of carboxylic acids on selectivity [a,b]

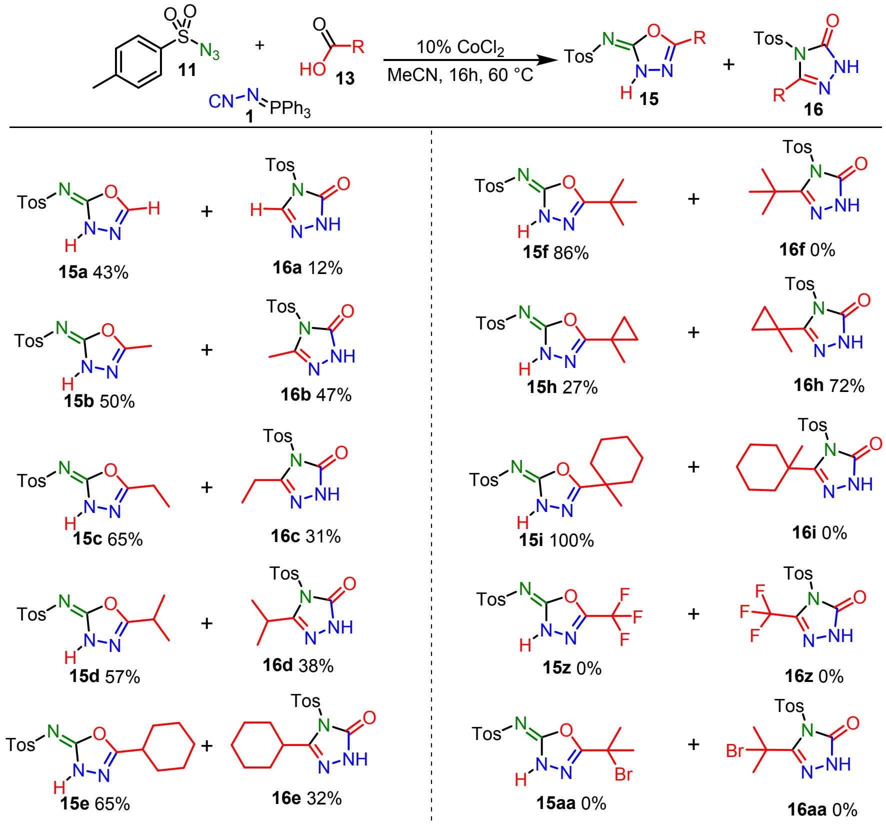

[a]Reaction conditions: **11** (0,25 mmol), **1** (0,3 mmol), **13** (0.5 mmol), MeCN (10 mL, 25 mM), 60 °C, 16 h. [b]Yields were determined by ^{1}H NMR using 2,5 dimethylfuran as internal standard

2.3 Unsuccessful results

In this scheme we present the carboxylic acids/azides that did not undergo the desired transformation under our optimized conditions.

Scheme S2: Unsuccessful results[a]

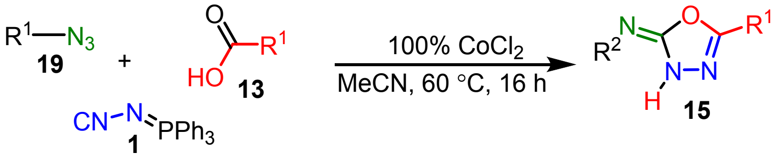

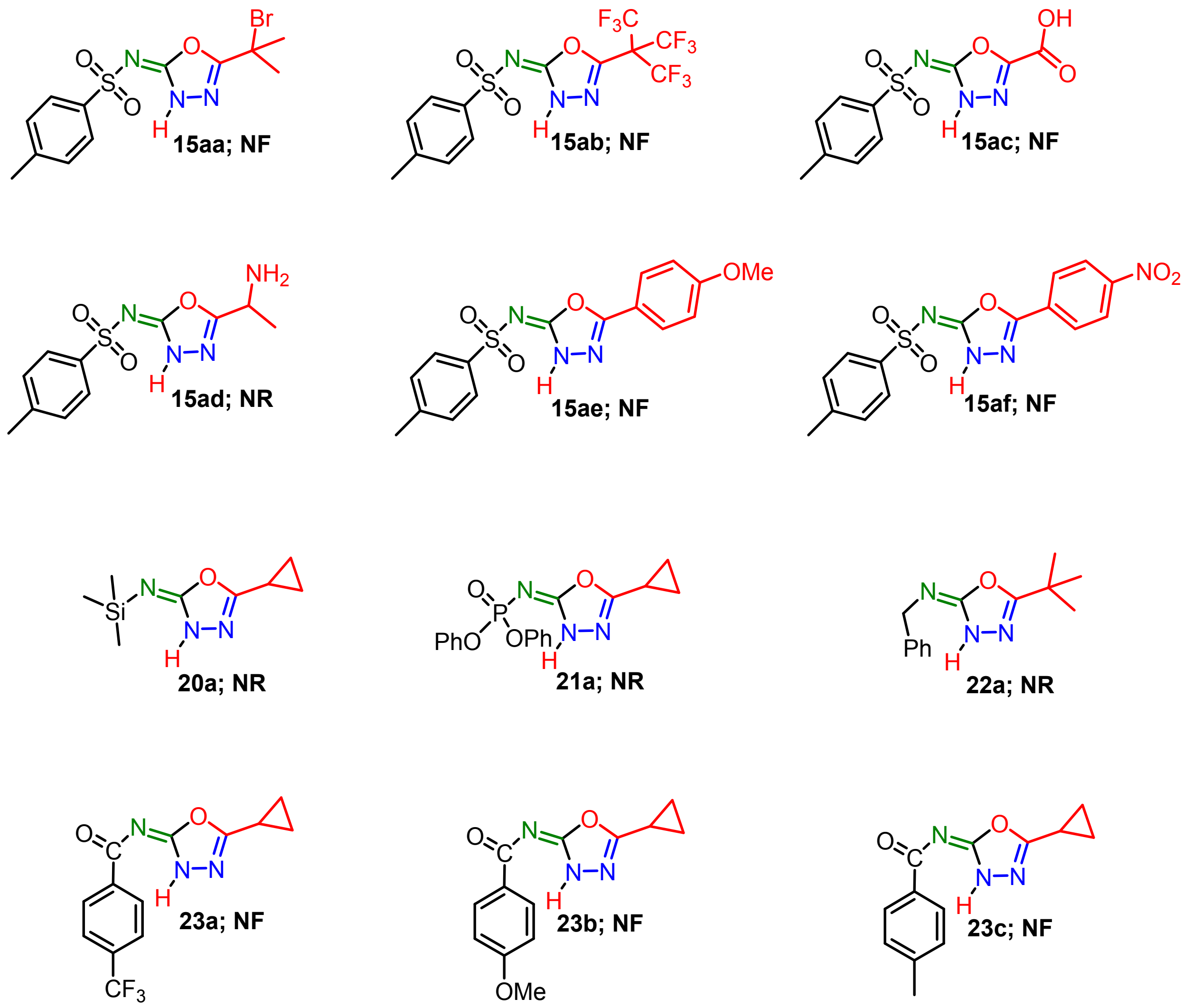

[a]Reaction conditions: **19**, (0.5 mmol), **1** (0.6 mmol), **13** (1.0 mmol), MeCN (20 mL, 25 mM), 60 °C, 16 h, under nitrogen atmosphere. NF = Not formed, but consumption of isocyanide and azide observerd. NR = No reaction.

2.4 Methods

Synthesis of sulfonyl azides (GP-A)[1]:

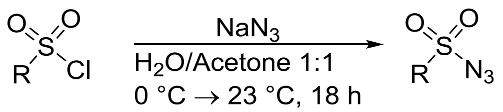

To a pre-cooled (0 °C), stirred, solution of sulfonyl chloride (5 mmol, 1 eq.), in acetone (5 mL, 1 M), NaN_3 (6 mmol, 1.2 eq.) dissolved in 5 mL water was added dropwise. After the addition of NaN_3 the ice bath was removed and the reaction was allowed to warm up to room temperature and the reaction was stirred overnight. The acetone was removed *in vacuo*, the water was diluted with ethyl acetate, and the water layer was extracted 3 times with ethyl acetate. The combined organic layers were washed with brine, filtered over Na_2SO_4, and concentrated *in vacuo* at 40 °C. The products were used without further purification.

Synthesis of acyl azides (GP-B)[2]:

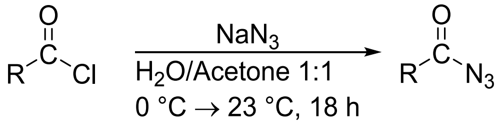

To a pre-cooled (0 °C), stirred, solution of sulfonyl chloride (5 mmol, 1 eq.), in acetone (5 mL, 1 M), NaN_3 (6 mmol, 1.2 eq.) dissolved in 5 mL water was added dropwise. After the addition of NaN_3 the ice bath was removed and the reaction was allowed to warm up to room temperature and the reaction was stirred overnight. the mixture was diluted with diethyl ether, and the water layer was extracted 3 times with diethyl ether. The combined organic layers were washed with brine, filtered over Na_2SO_4, and concentrated at atmospheric pressure at room temperature. The products were used without further purification.

General procedure synthesis of oxadiazoles 100% Co(II) loading (GP-C):

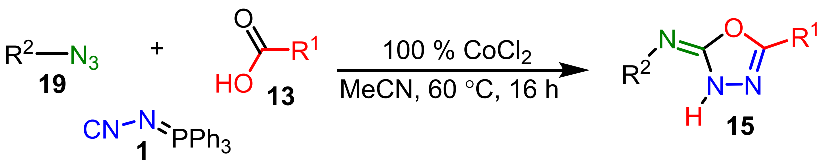

A flame-dried Schlenk was charged with a stirring bar and cobalt chloride (64.9 mg; 0.5 mmol; 1 equiv. and acid (1 mmol; 2 equiv. carboxylic acid **if solid**). The Schlenk was evacuated for 5 minutes, and then dried MeCN (10 mL, 50 mM) under a continuous nitrogen flow was added. After the catalyst was completely dissolved (dark blue solution), acid (1 mmol; 2 equiv. carboxylic acid **if liquid**) was added and the mixture was stirred for 10 minutes (stir for 30 minutes on larger scale). Then, *N*-

isocyanoiminotriphenylphosphorane (172 mg; 0.6 mmol; 1.2 eq) was added in one portion and the flask was rinsed with dried MeCN (10 mL, 25 mM) under continuous nitrogen flow. The mixture was stirred until the isocyanide was completely dissolved (brown solution). Azide (0.5 mmol; 1 eq) was added to the solution under continuous nitrogen flow. The mixture was heated to 60 °C in an oil bath and stirred for 16 h (Green solution). The reaction was concentrated *in vacuo* and re-dissolved in DCM. The organic layer was washed with HCl (1 M), subsequently the organic layer was extracted three times with NaOH (1 M) and the organic layer was discarded. The aqueous layer was acidified with HCl (12 M) until pH 1 and was extracted three times with DCM. The combined organic layers were dried over Na_2SO_4, filtered, and concentrated *in vacuo* at 40 °C. The compound is isolated as a white solid through chromatography with a solvent system range of 20% EtOAc to 50% EtOAc in heptane with 1% AcOH.

General procedure synthesis of oxadiazoles 10% Co(II) loading (GP-D):

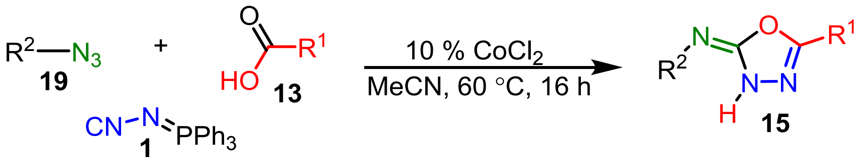

A flame-dried schlenk was charged with cobalt chloride (6.49 mg; 0.05 mmol; 0.1 eq) and acid (1 mmol; 2 eq. carboxylic acid **if solid**), evacuated for 5 minutes, and then dried MeCN (10 mL, 50 mM) under a continuous nitrogen flow was added. After the catalyst was completely dissolved (light blue solution), acid (1 mmol; 2 eq. carboxylic acid **if liquid**) was added and the mixture was stirred for 10 minutes (stir for 30 minutes on larger scale, dark blue solution). Then, *N*-isocyanoiminotriphenylphosphorane (172 mg; 0.6 mmol; 1 eq) was added in one portion and the flask was rinsed with dried MeCN (10 mL, 25 mM) under continuous nitrogen flow. The mixture was stirred until the isocyanide was completely dissolved (brown solution). *p*-toluenesulfonyl azide (0.5 mmol; 1 eq) was added to the solution under continuous nitrogen flow. Then the mixture was heated to 60 °C in an oil bath and stirred for 16 h (dark green solution). The reaction was concentrated *in vacuo* at 40 °C and re-dissolved in DCM. The organic layer was washed with HCl (1 M), subsequently the organic layer was extracted three times with NaOH (1 M) and the organic layer is discarded. The aqueous layer was acidified with HCl (12 M) until pH 1 and was extracted three times with DCM. The combined organic layers were dried over $MgSO_4$, filtered, and concentrated *in vacuo*. The compound is isolated as a white solid through chromatography with a solvent system range of 20% EtOAc to 50% EtOAc in heptane with 1% AcOH.

Detailed procedure for 1 mmol scale:

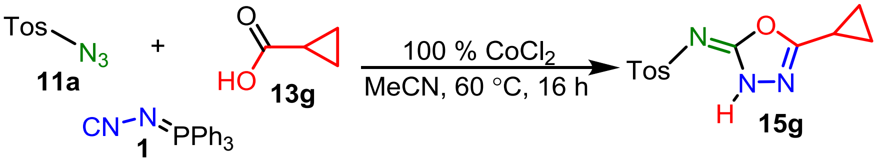

A flame-dried Schlenk was charged with a stirring bar and cobalt chloride (129.84 mg; 1,0 mmol; 1 equiv). The Schlenk was evacuated for 5 minutes, and then dried MeCN (20 mL, 50 mM) under a continuous nitrogen flow was added. After the catalyst was completely dissolved (dark blue solution), cyclopropanecarboxylic acid (172.18 mg, 159.28 µL, 2 mmol; 2 equiv) was added and the mixture was stirred for 30 minutes. Then, *N*-isocyanoiminotriphenylphosphorane (362.784 mg; 1.2 mmol; 1.2 equiv) was added in one portion and the flask was rinsed with dried MeCN (20 mL, 25 mM) under continuous nitrogen flow. The mixture was stirred until the isocyanide was completely dissolved (brown solution). *p*-toluenesulfonyl azide (197.21 mg, 1 mmol; 1 equiv) was added to the solution under continuous nitrogen flow. The mixture was heated to 60 °C in an oil bath and stirred for 16 h (Green solution). The reaction was concentrated *in vacuo* and re-dissolved in DCM. The organic layer was washed with HCl (1 M), subsequently the organic layer was extracted three times with NaOH (1 M) and the organic layer was discarded. The aqueous layer was acidified with HCl (12 M) until pH 1 and was extracted three times with DCM. The combined organic layers were dried over Na_2SO_4, filtered, and concentrated *in vacuo* at 40 °C. The compound is isolated as a white solid through column chromatography with a solvent system of 20% EtOAc in heptane with 1% AcOH. The product **15g** was isolated as a white solid (223 mg, 0.8 mmol, 80% yield).

General procedure for detosylation, generation of <u>free amine</u> (GP-E)[3]:

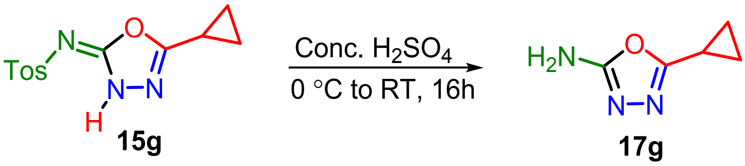

15g **17g**

Under air, a flask was charged with a stirring bar and 500 µL of H_2SO_4. The reaction mixture was cooled to 0 °C and 0.1 mmol of **15g** was added batch wise. After the addition of **15g**, the reaction was allowed to warm up to RT. After 12 h the reaction was quenched with saturated solution of $NaHCO_3$. The basic aqueous layer was extracted 3 times with DCM and the combined organic layers were dried over Na_2SO_4, filtered, and concentrated *in vacuo*.

Special remarks regarding methodology:

The acids of the reactions **15m**, **15n**, **15p** were not subjected to the described work-up due to their functionality. They were directly loaded onto the column. It is advised to dry-load them, however liquid loading also works. To synthesize **15o,** the TMS-protected alkyne **13o** was used and is deprotected during the work-up. The sterically congested acids, like **15d**, **15e**, **15f**, **15i**, **15j**, and **15k** can be problematic in the provided work-up, the foam that forms after adding the 1M NaOH takes up to one hour to dissipate. Loading them directly on the column also works and provides the same yield. When performing the column, absolutely use a gradient with increments of 5% due to the co-elution of the triphenylphosphine oxide and the desired product **15**. When the reaction has turned dark yellow/brown, the system contained oxygen or the acid did not provide the desired reaction. All products are difficult to re-dissolve in the following available solvents: DCM, $CHCl_3$, EtOAc, Acetone, Et_2O, DMF, and DMSO, however, upon heating or adding drops of acetic acid they should dissolve.

2.5 Characterization side product 16

To characterize product **16** confirmation of the ring structure was required. We started our NMR analysis with compound **16b**. Compound **16b** provided the following information. From the ^{15}N-^{1}H-HMBC of product **16b**, we could observe the correlation of nitrogen 4 and 1 with proton 6. However, we could not observe the correlation between proton 6 and nitrogen 3 (^{15}N-^{1}H-HMBC) or carbonyl carbon 2 (^{13}C-^{1}H-HMBC). To gather more evidence on the ring structure, we also analyzed product **16a** possessing a proton directly bound to C5. Here in the ^{13}C-^{1}H-HMBC spectrum a correlation between proton 5 and carbonyl carbon 2 was observed. Additionally, the ^{15}N-^{1}H-HMBC reveiled the correlation between all nitrogen atoms, N1, N4, N3, and proton 5, supporting the ring structure proposed for **16**.

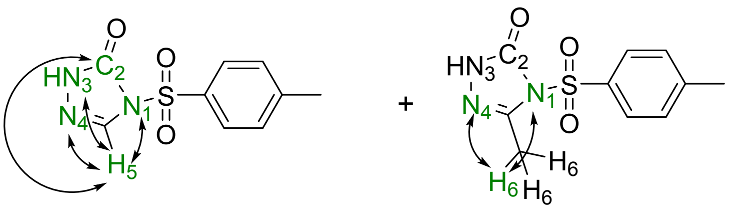

16a **16b**

NMR analysis of product **16a:**

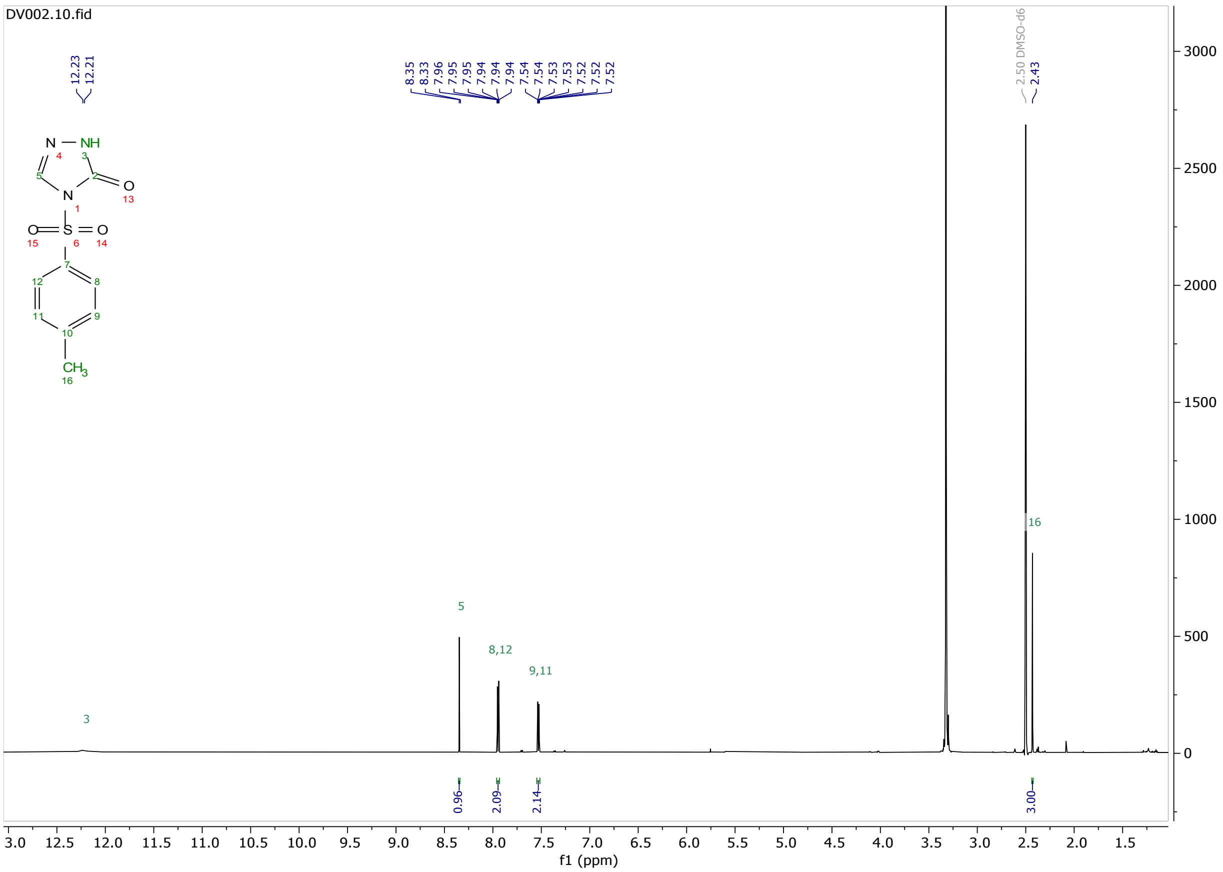

^{1}H spectrum **16a**

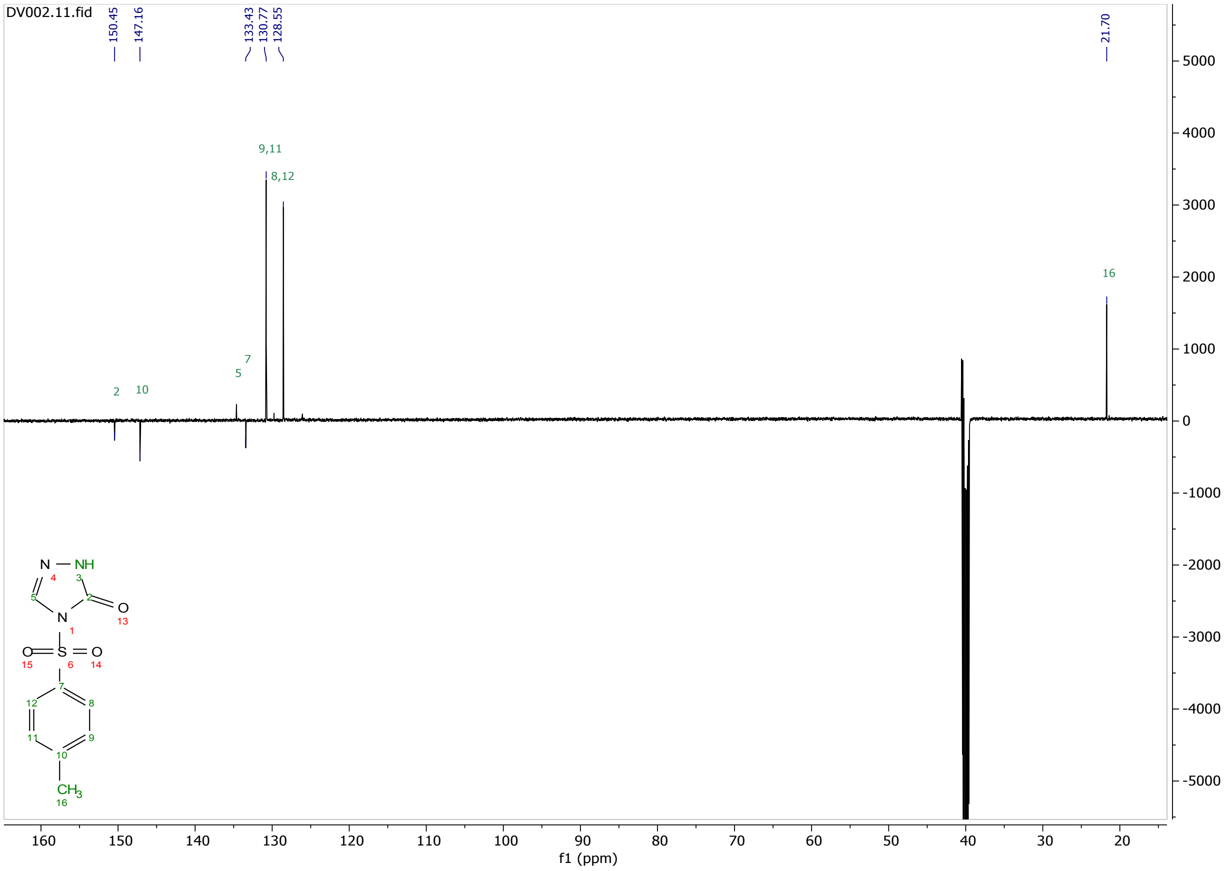

DEPTQ spectrum **16a**

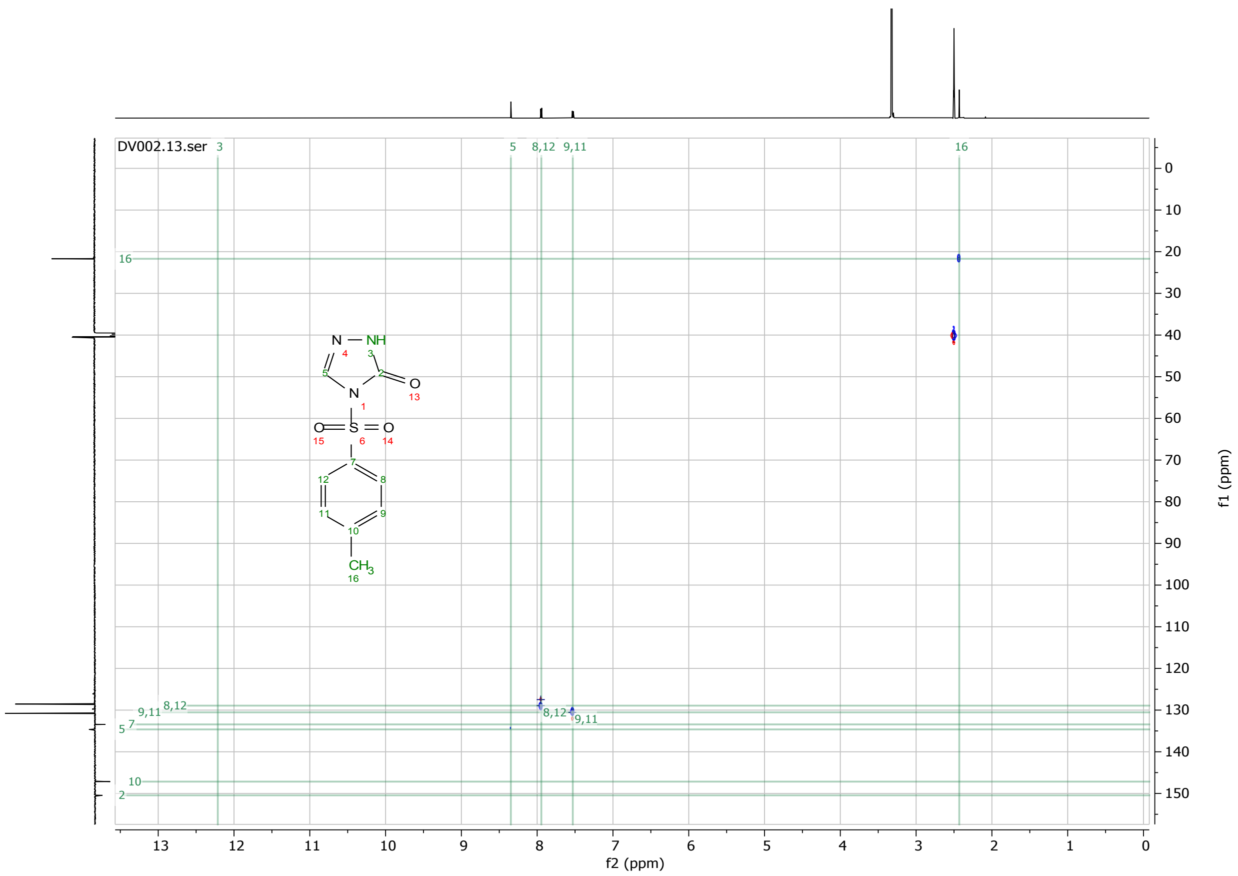

HSQC spectrum **16a**

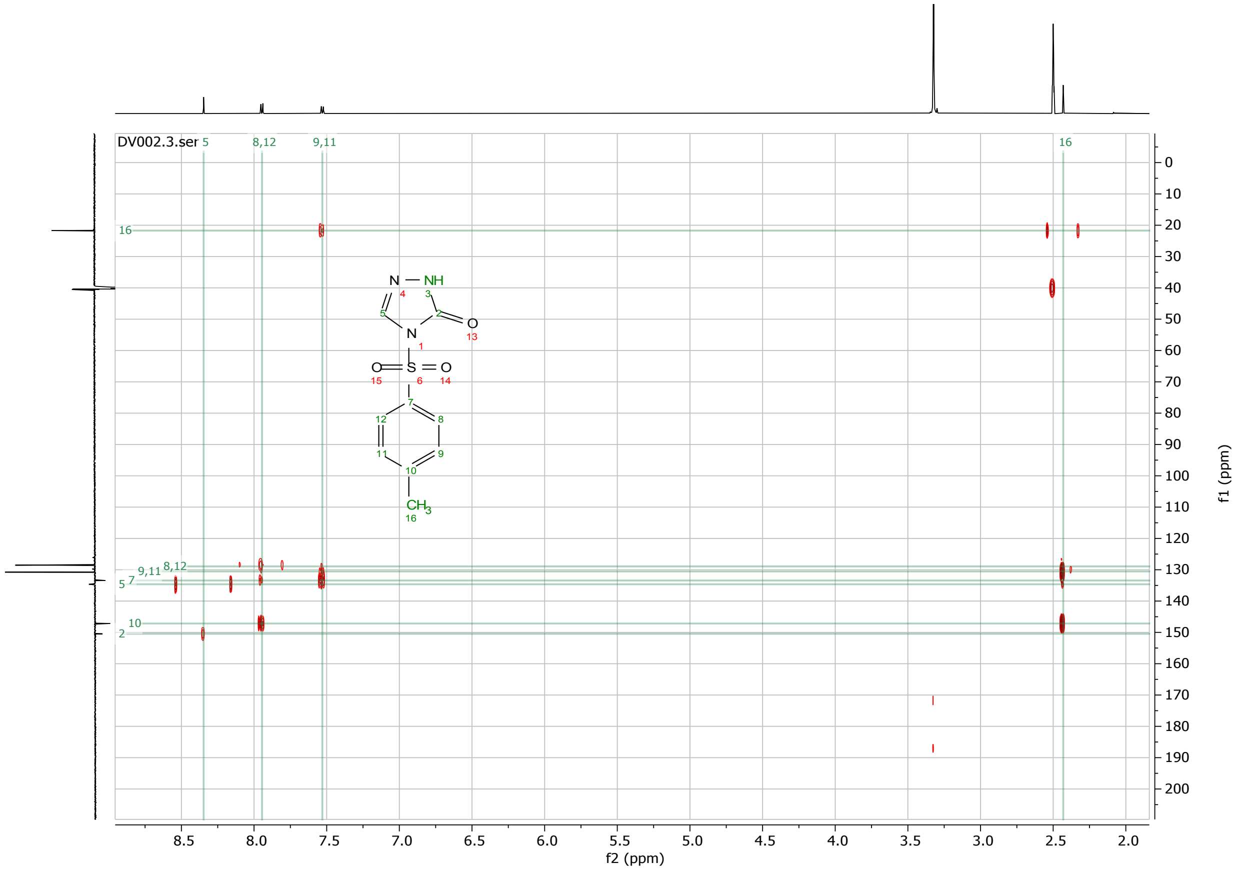

^{1}H-^{13}C HMBC spectrum **16a**

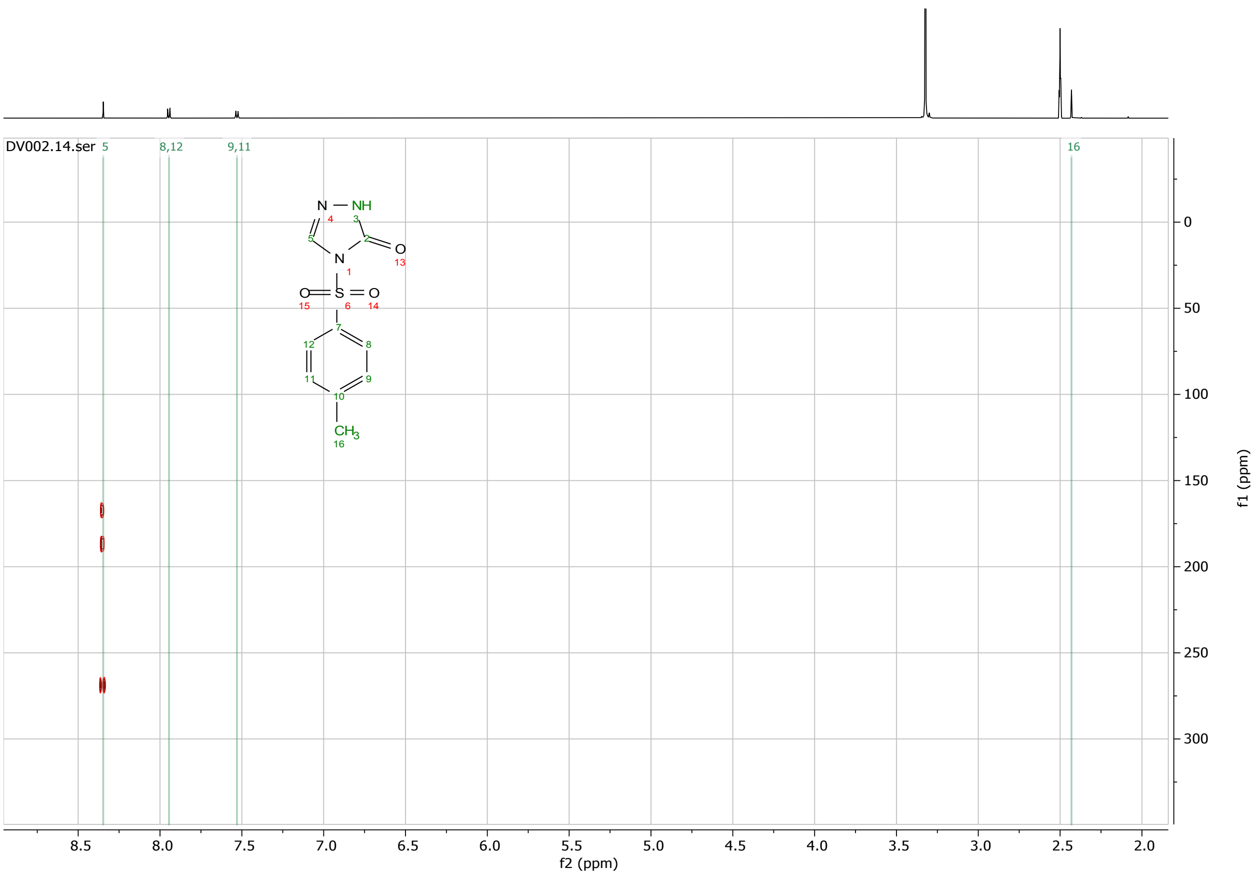

^{1}H-^{15}N HMBC spectrum **16a**

NMR analysis of product **16b**:

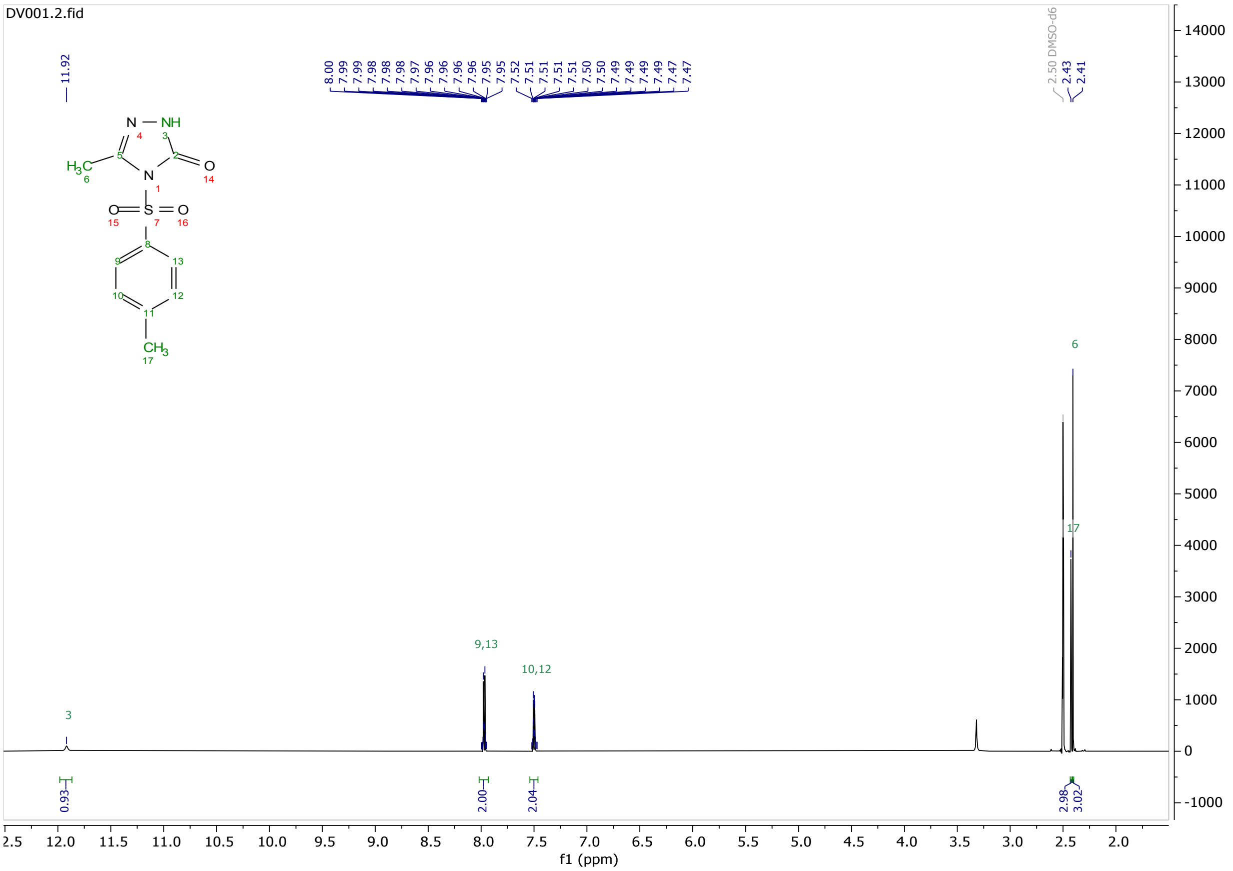

^{1}H spectrum **16b**

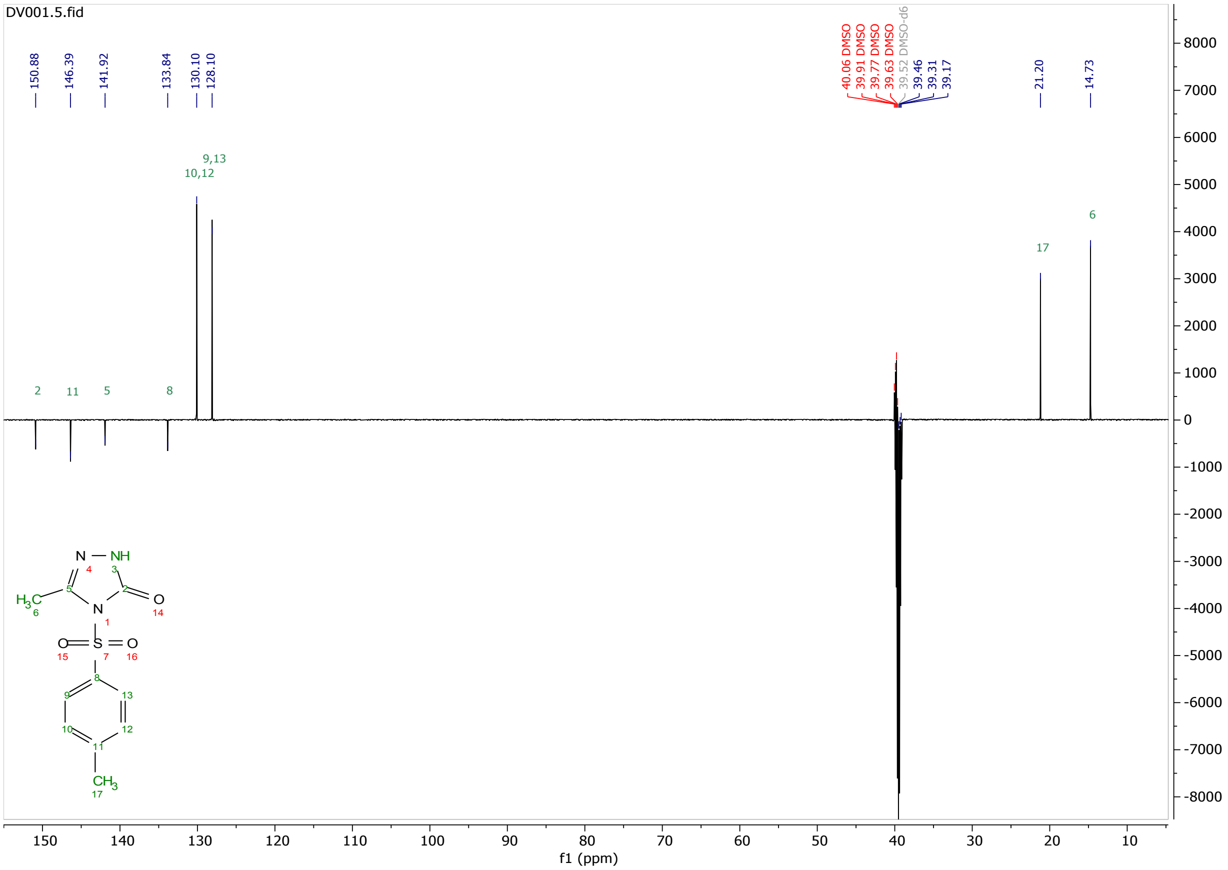

DEPTQ spectrum **16b**

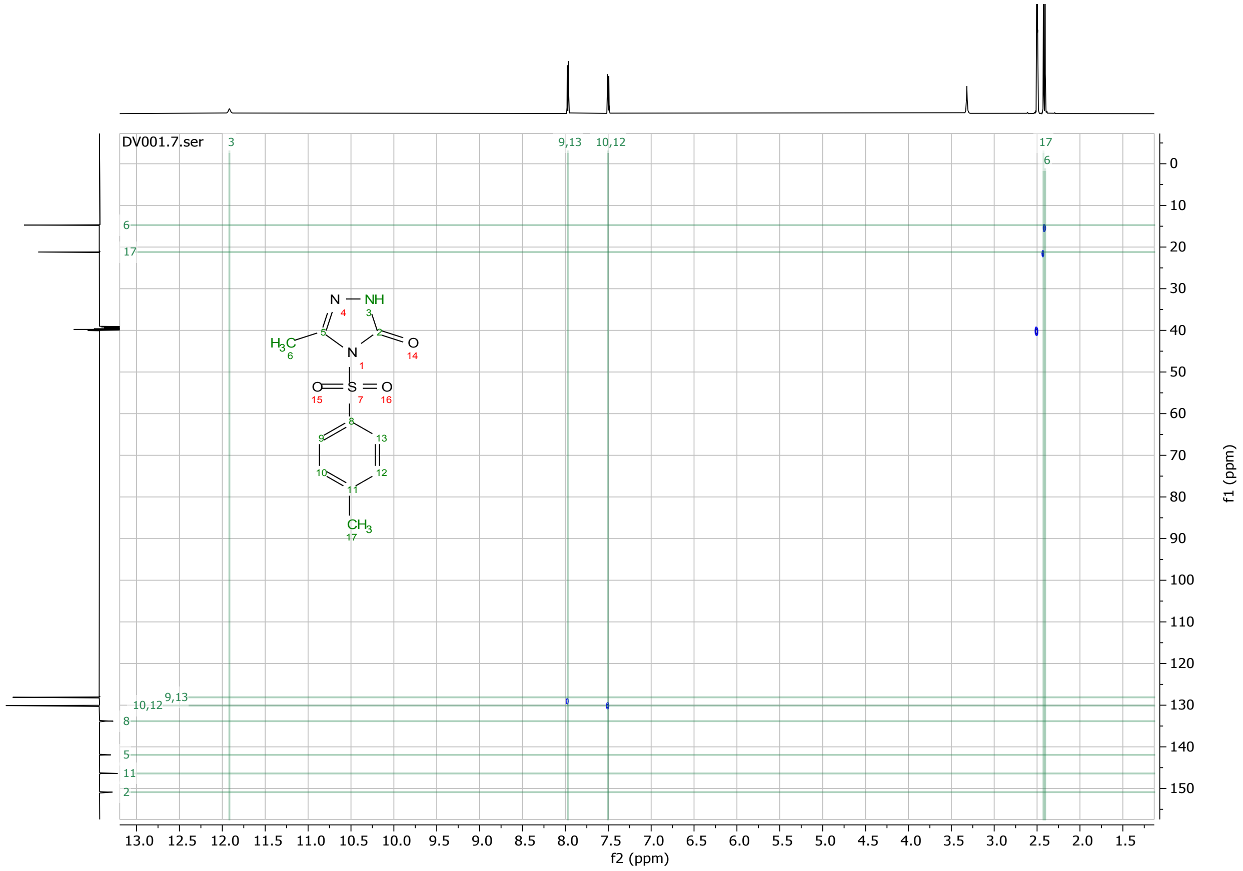

HSQC spectrum **16b**

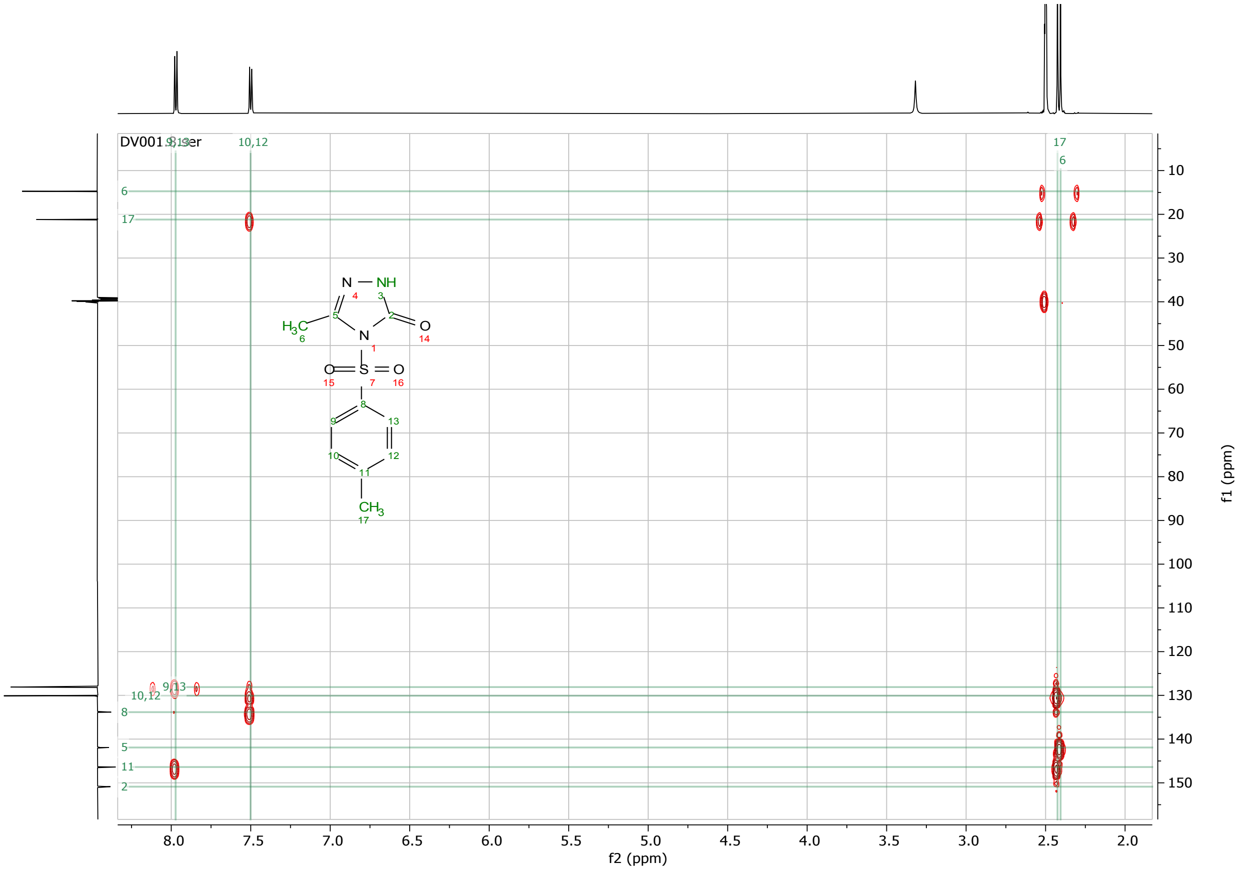

^{1}H-^{13}C HMBC spectrum **16b**

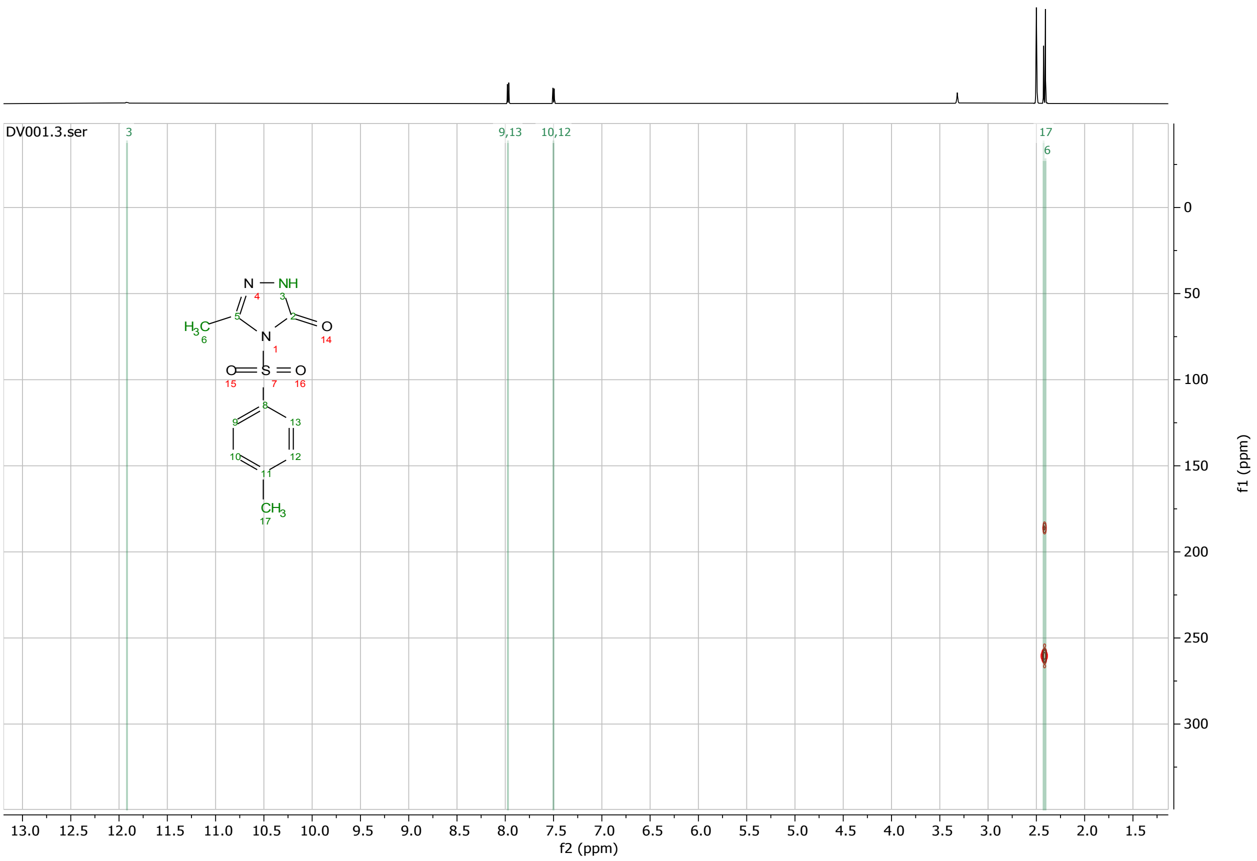

^1H-^{15}N HMBC spectrum **16b**

2.6 X-ray analysis

The main interactions in the crystal are an intramolecular hydrogen bond motif, where the oxadiazole hydrogen hydrogen H3 bonds to a sulfamide oxygen (N-H: 1.02(2)Å, N-O 2.805(2) Å, N-H…O 107.0(15)° and an intermolecular $\pi-\pi$ interactions between the faces of the toluene rings and oxadiazole rings.

CCDC 2225280 contains the supplementary crystallographic data for this paper. These data are provided free of charge by The Cambridge Crystallographic Data Centre.

Crystal data: $C_{10}H_{11}N_3O_3S$; M_r 253.28; Triclinic, P-1; T(K) 293, cell parameters (Å, °) 6.9975 (5), 8.7725 (11), 10.2995 (8) 80.501 (9), 82.193 (6), 70.376 (7); V (Å3) 585.15 (10) Z=2 μ (mm^{-1}) 2.50 Crystal size (mm) 0.2 × 0.2 × 0.2 Tmin, Tmax 0.887, 1.107 No. of measured, independent and observed [$I > 2\sigma(I)$] reflections 7763, 2100, 1960 R_{int} 0.039 ($(\sin \theta/\lambda)_{max}$ ($Å^{-1}$) 0.617; $R[F^2 > 2\sigma(F^2)]$, $wR(F^2)$, S 0.031, 0.084, 1.10 No. of reflections: 2100; No. of parameters: 254; H-atom treatment: All H-atom parameters refined; $\Delta\rho_{max}$, $\Delta\rho_{min}$ (e $Å^{-3}$) 0.16, -0.14

Figure S3: Anisotropic displacement plot of the asymmetric unit of the structure of **15b**. Ellipsoids are drawn at the 50% probability level.

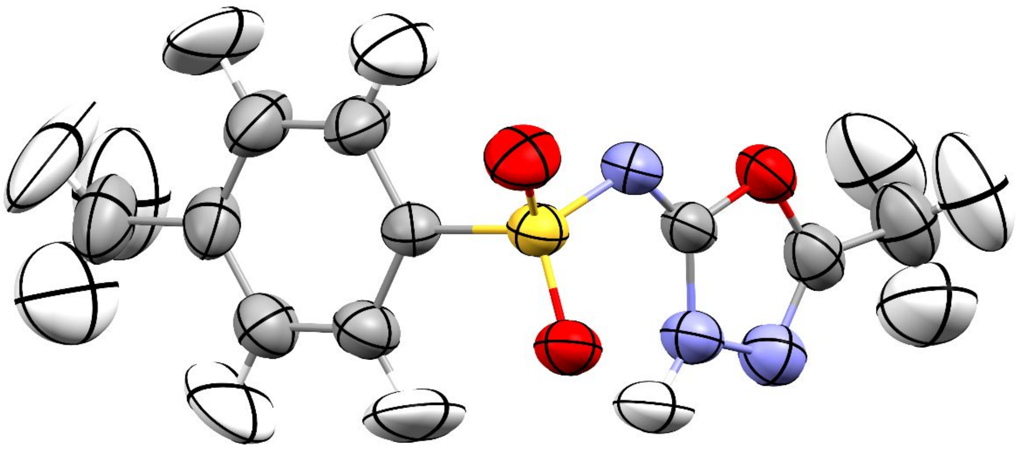

2.7 References

Crystallography references:

1.) Rigaku, CrystalClear SM-Expert v2.1 b32, April 12, 2014.
2.) Minor, W.; Cymborowski, M.; Otwinowski, Z.; Chruszcz, M. HKL-3000: the integration of data reduction and structure solution--from diffraction images to an initial model in minutes. *Acta Crystallographica Section D Structural Biology*, **2006**, *62*, 859-66.
3.) Sheldrick, G., *Acta Crystallogr. Sect. C* **2015**, *71*, 3-8.
4.) Sheldrick, G., *Acta Crystallogr. Sect. A* **2015**, *71*, 3-8.
5.) Hansen, N. K. ; Coppens, P. *Acta Cryst.* **1978**. *A34*, 909-921.
6.) Chodkiewicz, M. L. ; Migacz, S. ; Rudnicki, W. ; Makal, A. ; Kalinowski, J.A. ; Moriarty, N.W. ; Grosse - Kunstleve, R.W. ; Afonine, P.V. ; Adams, P.D. ; Dominiak, P.M. DiSCaMB : a software library for aspherical atom model X - ray scattering factor calculations with CPUs and GPUs. *J.Appl.Cryst.* **2018**, *51*, 193 - 199.
7.) Kumar, P.; Gruza, B.; Bojarowski, A.; Dominiak, P.M. Extension of the transferable aspherical pseudoatom data bank for the comparison of molecular electrostatic potentials in structure–activity studies. *Acta Cryst.A,* **2019**, 75, 398 - 408.
8.) Jha et., al., *Acta Cryst.B*, **2020**, *76*, 296 – 306.
9.) Kleemiss, F. ; Grabowsky, S. ; Dolomanov, O. V.; Puschman ***Chem. Sci.***, 2021,**12**, 1675-1692.
10.) Bourhis, L. J. ; Dolomanov, O. V. ; Gildea R. J.; Howard, J. A. K.; Puschmann, H. *Acta Cryst. A.* **2015,** *71*, 59-75.
11.) Dolomanov, O. V. ; Bourhis, L. J.; Gildea, R. J.; Howard, J. A. K.; Puschmann, H. *J. Appl. Cryst.* **2009,** *42*, 339-341.
12.) Gildea, R. J. ; Bourhis, L. J. ; Dolomanov, O. V. ; Grosse - Kunstleve, R. W. ; Puschmann, H.; Adams, P. D.; Howard J. A. K. a comprehensive CIF toolbox. *J.Appl.Cryst.* **2011**, *44*, 1259 - 1263.

Synthesis references:

13.) Gu, Z.-Y.; Liu, Y.; Wang, F.; Bao, X.; Wang, S.-J.; Ji, S-J *ACS Catal.* **2017**, *7*, *6*, 3893–3899.
14.) Shin, K.; Ryu, J.; Chang, S. *Org. Lett.* **2014**, *16*, *7*, 2022–2025.
15.) Li, H.; Deng, H. *Synthesis*, **2017,** *49*, 2711–2720.
16.) Meo, P.; Khan, N. Antibiotic Compounds. Patent WO 2018037223A1, March 1, 2018.

3.1 Spectral data

(15a):

(*E*)-4-methyl-*N*-(1,3,4-oxadiazol-2(3H)-ylidene)benzenesulfonamide:

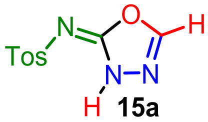

15a

Product **15a** was synthesized according to **GP-C** and was isolated, via column chromatography using the reported TLC system with a gradient from heptane increasing in 5% steps until the reported ethyl acetate concentration, as a white solid (48 mg, 0.2 mmol, 40% yield).

TLC system: 30% EtOAc in heptane; 1% AcOH.

^{1}H spectrum : (300 MHz, $CDCl_3$) δ 7.91 – 7.80 (m, 3H), 7.28 (d, *J* = 7.9 Hz, 2H), 2.41 (s, 3H).

^{13}C spectrum : (75 MHz, $CDCl_3$) δ 155.9, 145.6, 142.9, 138.9, 129.2, 126.4, 21.3.

HRMS (ESI): *m/z* calculated for: $C_9H_9N_3O_3S^+$ $[M+Na]^+$ 262.0257, found 262.0281.

(15b):

(*E*)-4-methyl-*N*-(5-methyl-1,3,4-oxadiazol-2(3H)-ylidene)benzenesulfonamide:

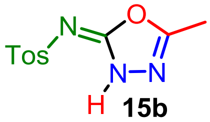

15b

Product **15b** was synthesized according to **GP-C** and was isolated, via column chromatography using the reported TLC system with a gradient from heptane increasing in 5% steps until the reported ethyl acetate concentration, as a white solid (63 mg, 0.25 mmol, 50% yield).

TLC system: 50% EtOAc in heptane; 1% AcOH.

^{1}H spectrum : (300 MHz, $CDCl_3$) δ 7.83 (d, *J* = 8.3 Hz, 2H), 7.29 (d, *J* = 8.0 Hz, 2H), 2.41 (s, 3H), 2.36 (s, 3H).

^{13}C spectrum : (75 MHz, $CDCl_3$) δ 157.3, 155.8, 143.5, 138.7, 129.5, 126.4, 21.5, 11.2.

HRMS (ESI): *m/z* calculated for: $C_{10}H_{11}N_3O_3S^+$ $[M+H]^+$ 254.0594, found 254.0582.

(15c):

(*E*)-*N*-(5-ethyl-1,3,4-oxadiazol-2(3H)-ylidene)-4-methylbenzenesulfonamide:

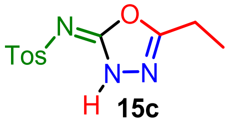

15c

Product 15c was synthesized according to GP-C and was isolated, via column chromatography using the reported TLC system with a gradient from heptane increasing in 5% steps until the reported ethyl acetate concentration, as a white solid (85 mg, 0.32 mmol, 64% yield).

TLC system: 20% EtOAc in heptane; 1% AcOH.

^{1}H spectrum : (300 MHz, $CDCl_3$) δ 7.83 (d, J = 8.3 Hz, 2H), 7.29 (d, J = 7.9 Hz, 2H), 2.68 (q, J = 7.6 Hz, 2H), 2.41 (s, 3H), 1.30 (t, J = 7.6 Hz, 3H).

^{13}C spectrum : (75 MHz, $CDCl_3$) δ 159.9, 157.3, 143.6, 138.8, 129.6, 126.5, 21.7, 19.3, 9.8.

HRMS (ESI): *m/z* calculated for: $C_{11}H_{13}N_3O_3S^+$ $[M+H]^+$ 268.0750, found 268.0740.

(15d):

(*E*)-*N*-(5-isopropyl-1,3,4-oxadiazol-2(3H)-ylidene)-4-methylbenzenesulfonamide:

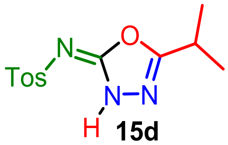

15d

Product 15d was synthesized according to GP-C and was isolated, via column chromatography using the reported TLC system with a gradient from heptane increasing in 5% steps until the reported ethyl acetate concentration, as a white solid (64 mg, 0.23 mmol, 46%).

TLC system: 20% EtOAc in heptane; 1% AcOH.

^{1}H spectrum : (300 MHz, $CDCl_3$) δ 7.85 (d, J = 8.3 Hz, 2H), 7.28 (d, J = 8.2 Hz, 2H), 2.95 (p, J = 7.0 Hz, 1H), 2.40 (s, 3H), 1.30 (d, J = 7.0 Hz, 6H).

^{13}C spectrum : (75 MHz, $CDCl_3$) δ 162.9, 157.1, 143.5, 143.4, 138.7, 129.7, 129.5, 126.5, 26.4, 21.6, 18.9.

HRMS (ESI): *m/z* calculated for: $C_{12}H_{15}N_3O_3S^+$ $[M+H]^+$ 282.0907, found 282.0906.

(15e):

(*E*)-*N*-(5-cyclohexyl-1,3,4-oxadiazol-2(3H)-ylidene)-4-methylbenzenesulfonamide:

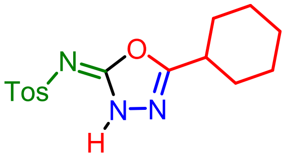

15e

Product **15e** was synthesized according to GP-C and was isolated, via column chromatography using the reported TLC system with gradient from heptane increasing in 5% steps until the reported ethyl acetate concentration, as a white solid (96 mg, 0.3 mmol, 60% yield).

TLC system: 20% EtOAc in heptane; 1% AcOH.

^{1}H spectrum : (300 MHz, $CDCl_3$) δ 7.85 (d, *J* = 8.3 Hz, 2H), 7.26 (d, *J* = 8.1 Hz, 2H), 2.66 (tt, *J* = 11.2, 3.7 Hz, 1H), 2.39 (s, 3H), 1.96 (d, *J* = 12.5 Hz, 2H), 1.77 (dt, *J* = 7.2, 3.6 Hz, 2H), 1.67 (dd, *J* = 10.3, 4.5 Hz, 1H), 1.53 – 1.38 (m, 2H), 1.37 – 1.18 (m, 3H).

^{13}C spectrum : (75 MHz, $CDCl_3$) δ162.0, 156.9, 143.3, 138.7, 129.5, 126.5, 34.9, 29.0, 25.4, 25.0, 21.5.

HRMS (ESI): *m/z* calculated for: $C_{15}H_{19}N_3O_3S^+$ $[M+H]^+$ 322.1220, found 322.1231.

(15f):

(*E*)-*N*-(5-(tert-butyl)-1,3,4-oxadiazol-2(3H)-ylidene)-4-methylbenzenesulfonamide:

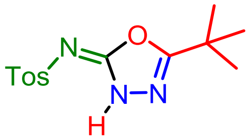

15f

Product **15f** was synthesized according to GP-D and was isolated, via column chromatography using the reported TLC system with a gradient from heptane increasing in 5% steps until the reported ethyl acetate concentration, as a white solid in (106 mg, 0.36 mmol, 71% yield).

TLC system: 30% EtOAc in heptane; 1% AcOH.

^{1}H spectrum : (300 MHz, $CDCl_3$) δ 7.85 (d, *J* = 8.2 Hz, 2H), 7.30 (d, *J* = 8.0 Hz, 2H), 2.42 (s, 3H), 1.33 (s, 9H).

^{13}C spectrum : (75 MHz, $CDCl_3$) δ 165.2, 157.4, 143.6, 138.8, 129.7, 126.6, 32.7, 27.3, 21.7.

HRMS (ESI): *m/z* calculated for: $C_{13}H_{17}N_3O_3S^+$ $[M+H]^+$ 296.1063, found 296.1061.

(15g):

(*E*)-*N*-(5-cyclopropyl-1,3,4-oxadiazol-2(3H)-ylidene)-4-methylbenzenesulfonamide:

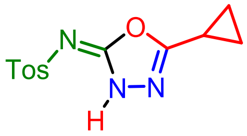

15g

Product **15g** was synthesized according to GP-C and was isolated, via column chromatography using the reported TLC system with a gradient from heptane increasing in 5% steps until the reported ethyl acetate concentration, as a white solid (111 mg, 0.4 mmol 80% yield).

TLC system: 30% EtOAc in heptane; 1% AcOH.

^{1}H spectrum : (300 MHz, $CDCl_3$) δ 7.84 (d, *J* = 8.3 Hz, 2H), 7.28 (d, *J* = 9.0 Hz, 2H), 2.41 (s, 2H), 2.02 – 1.87 (m, 2H), 1.20 – 1.01 (m, 4H).

^{13}C spectrum : (75 MHz, $CDCl_3$) δ 160.3, 156.8, 143.5, 138.7, 129.5, 126.7, 21.6, 7.7, 6.4.

HRMS (ESI): *m/z* calculated for: $C_{12}H_{13}N_3O_3S^+$ $[M+H]^+$ 280.0750, found 280.0753.

(15h):

(*E*)-4-methyl-*N*-(5-(1-methylcyclopropyl)-1,3,4-oxadiazol-2(3H)-ylidene)benzenesulfonamide:

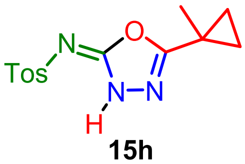

15h

Product **15h** was synthesized according to GP-C and was isolated, via column chromatography using the reported TLC system with a gradient from heptane increasing in 5% steps until the reported ethyl acetate concentration, as a white solid (73 mg, 0.25 mmol, 50% yield).

TLC System: 30% EtOAc in heptane; 1% AcOH.

^{1}H spectrum : (300 MHz, $CDCl_3$) δ 7.84 (d, *J* = 8.1 Hz, 2H), 7.28 (d, *J* = 8.0 Hz, 2H), 2.41 (s, 3H), 1.41 (s, 3H), 1.19 (m, 4H).

^{13}C spectrum : (75 MHz, $CDCl_3$) δ 162.4, 157.1, 143.6, 138.8, 129.6, 126.5, 21.7, 19.3, 15.8, 12.9.

HRMS (ESI): *m/z* calculated for: $C_{13}H_{15}N_3O_3S^+$ $[M+Na]^+$ 316.0726, found 316.0727.

(15i):

(*E*)-4-methyl-*N*-(5-(1-methylcyclohexyl)-1,3,4-oxadiazol-2(3H)-ylidene)benzenesulfonamide:

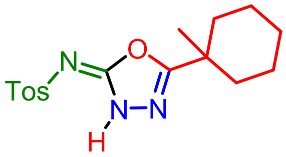

15i

Product **15i** was synthesized according to procedure GP-D and isolated, via column chromatography using the reported TLC system with a gradient from heptane increasing in 5% steps until the reported ethyl acetate concentration, as a white solid (70 mg, 0.24 mmol, 48% yield).

TLC System: 30% EtOAc in heptane; 1% AcOH.

^{1}H spectrum : (300 MHz, $CDCl_3$) δ 7.86 (d, *J* = 8.3 Hz, 2H), 7.29 (d, *J* = 8.2 Hz, 2H), 2.41 (s, 3H), 2.06 – 1.91 (m, 2H), 1.62 – 1.31 (m, 8H), 1.26 (s, 3H).

^{13}C spectrum : (75 MHz, $CDCl_3$) δ 164.3, 157.0, 143.4, 138.7, 129.5, 126.5, 36.5, 35.1, 26.2, 25.3, 22.1, 21.6.

HRMS (ESI): *m/z* calculated for: $C_{16}H_{21}N_3O_3S^+$ $[M+H]^+$ 336.1376, found 336.1382

(15j):

***N*-((*E*)-5-(adamantan-1-yl)-1,3,4-oxadiazol-2(3H)-ylidene)-4-methylbenzenesulfonamide:**

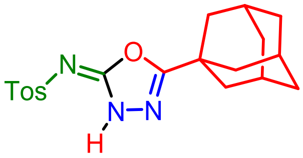

15j

Product **15j** was synthesized according to procedure GP-D and isolated, via column chromatography using the reported TLC system with a gradient from heptane increasing in 5% steps until the reported ethyl acetate concentration, as a white solid (41 mg, 0.11 mmol, 22% yield).

TLC System: 20% EtOAc in heptane; 1% AcOH

^{1}H spectrum : (300 MHz, $CDCl_3$) δ 7.85 (d, *J* = 8.3 Hz, 2H), 7.29 (d, *J* = 8.1 Hz, 2H), 2.41 (s, 3H), 2.14 – 2.03 (m, 3H), 1.93 (d, *J* = 2.9 Hz, 6H), 1.84 – 1.65 (m, 6H).

^{13}C spectrum : (75 MHz, $CDCl_3$) δ 164.6, 157.2, 143.5, 129.6, 126.5, 38.9, 36.1, 34.4, 27.4, 21.6.

HRMS (ESI): m/z calculated for: $C_{19}H_{23}N_3O_3S^+$ $[M+H]^+$ 374.1533, found 374.1562

(15k):

(*E*)-4-methyl-*N*-(5-(1,1,1-trifluoro-2-methylpropan-2-yl)-1,3,4-oxadiazol-2(3H)-ylidene)benzenesulfonamide:

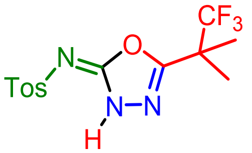

15k

Product **15k** was synthesized according to GP-C and isolated, via column chromatography using the reported TLC system with a gradient from heptane increasing in 5% steps until the reported ethyl acetate concentration, as a white solid (61 mg, 0.175 mmol, 35% yield).

TLC System: 40% EtOAc in heptane; 1% AcOH.

^{1}H spectrum : (300 MHz, $CDCl_3$; DMSO) δ 7.67 (d, *J* = 8.3 Hz, 2H), 7.10 (d, *J* = 6.9 Hz, 2H), 2.22 (s, 3H), 1.35 (s, 6H).

^{19}F spectrum : (282 MHz, $CDCl_3$) δ -71.13.

^{13}C spectrum : (75 MHz, Acetone) δ 157.72 (m), 144.01, 140.80, 130.30, 127.39, 127.08 (q, J = 289.3 Hz), 127.04, 43.29 (q, J = 28.0 Hz), 21.48, 19.51 (q, J = 2.0 Hz).

HRMS (ESI): *m/z* calculated for: $C_{13}H_{15}N_3O_3S^+$ [M+H]$^+$ 350.0781, found 350.0771.

(15m):

tert-butyl (*E*)-(1-(5-(tosylimino)-4,5-dihydro-1,3,4-oxadiazol-2-yl)ethyl)carbamate:

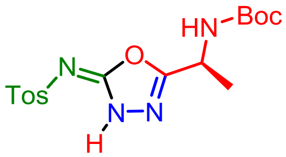

15m

Product **15m** was synthesized according to GP-C and isolated, via column chromatography using the reported TLC system with a gradient from heptane increasing in 5% steps until the reported ethyl acetate concentration, as a white solid (114 mg, 0.3 mmol, 60% yield).

TLC System: 40% EtOAc (gradient) in heptane; 1% AcOH.

^{1}H spectrum : (300 MHz, $CDCl_3$) δ 9.87 (bs, 1H), 8.06 (d, *J* = 8.0 Hz, 2H), 7.36 (d, *J* = 8.1 Hz, 2H), 5.33 (bs, 2H), 2.44 (s, 3H), 1.57 (d, *J* = 6.5 Hz, 3H), 1.43 (s, 9H).

^{13}C spectrum : (75 MHz, $CDCl_3$) δ 154.8, 146.7, 133.5, 129.9, 129.1, 44.8, 28.4, 21.8, 20.7.

HRMS (ESI): m/z calculated for: $C_{16}H_{22}N_4O_5S^+$ $[M+Na]^+$ 405,1203, found 405,1194.

(15n):

(*E*)-*N*-(5-(but-3-en-1-yl)-1,3,4-oxadiazol-2(3H)-ylidene)-4-methylbenzenesulfonamide:

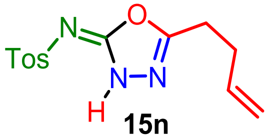

15n

Product **15n** was synthesized according to procedure GP-C and isolated, via column chromatography using the reported TLC system with a gradient from heptane increasing in 5% steps until the reported ethyl acetate concentration, as white solid (120 mg, 0.41 mmol, 82% yield).

TLC System: 40% EtOAc in heptane; 1% AcOH.

^{1}H spectrum : (300 MHz, $CDCl_3$) δ 7.83 (d, *J* = 8.1 Hz, 2H), 7.29 (d, *J* = 8.1 Hz, 2H), 5.77 (ddd, *J* = 16.8, 10.3, 6.7 Hz, 1H), 5.16 – 5.02 (m, 2H), 2.75 (t, *J* = 7.4 Hz, 2H), 2.50 – 2.44 (m, 2H), 2.41 (s, 3H).

^{13}C spectrum : (75 MHz, $CDCl_3$) δ 158.4, 157.2, 143.5, 138.7, 134.8, 129.6, 126.5, 117.3, 29.2, 25.0, 21.6.

HRMS (ESI): *m/z* calculated for: $C_{13}H_{15}N_3O_3S^+$ $[M+H]^+$ 294.0907, found 294.0920.

(15o):

(*E*)-*N*-(5-ethynyl-1,3,4-oxadiazol-2(3H)-ylidene)-4-methylbenzenesulfonamide:

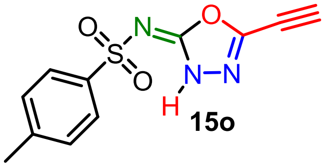

15o

Product **15o** was synthesized according to procedure GP-C and isolated, via column chromatography using the reported TLC system with a gradient from heptane increasing in 5% steps until the reported ethyl acetate concentration, as white solid (43 mg, 0.165 mmol, 33% yield).

TLC System: 40% EtOAc in heptane; 1% AcOH.

^{1}H spectrum : (300 MHz, $CDCl_3$) δ 7.83 (d, *J* = 8.4 Hz, 2H), 7.30 (d, *J* = 8.2 Hz, 2H), 3.50 (s, 1H), 2.42 (s, 3H).

^{13}C spectrum : (75 MHz, $CDCl_3$) δ 155.9, 143.9, 141.0, 138.2, 129.7, 126.7, 86.5, 66.3, 21.7.

HRMS (ESI): m/z calculated for: $C_{11}H_9N_3O_3S^+$ $[M+H]^+$ 264.0437, found 264.0428.

(15p):

(*E*)-4-methyl-*N*-(5-(3-oxobutyl)-1,3,4-oxadiazol-2(3H)-ylidene)benzenesulfonamide:

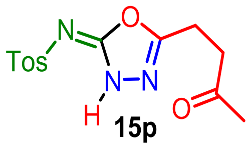

15p

Product **15p** was synthesized according to procedure GP-C and isolated, via column chromatography using the reported TLC system with a gradient from heptane increasing in 5% steps until the reported ethyl acetate concentration, as white solid (100 mg, 0.325 mmol, 65% yield).

TLC System: 40% EtOAc in heptane; 1 % AcOH.

^{1}H spectrum : (300 MHz, $CDCl_3$) δ 7.82 (d, *J* = 8.3 Hz, 2H), 7.29 (d, *J* = 8.2 Hz, 2H), 2.98 – 2.82 (m, 4H), 2.41 (s, 3H), 2.21 (s, 3H).

^{13}C spectrum : (75 MHz, $CDCl_3$) δ 205.0, 158.2, 157.1, 143.6, 138.7, 129.6, 126.5, 37.9, 29.9, 21.7, 19.6.

HRMS (ESI): m/z calculated for: $C_{13}H_{15}N_3O_4S^+$ $[M+Na]^+$ 332.0675, found 332.0668.

(15q):

(*E*)-4-methyl-*N*-(5-(prop-1-yn-1-yl)-1,3,4-oxadiazol-2(3H)-ylidene)benzenesulfonamide:

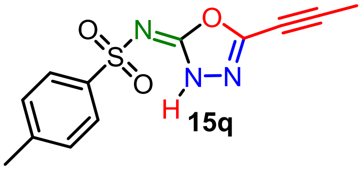

15q

Product **15q** was synthesized according to procedure GP-C and isolated, via column chromatography using the reported TLC system with a gradient from heptane increasing in 5% steps until the reported ethyl acetate concentration, as white solid (97 mg, 0.35 mmol, 70% yield).

TLC System: 30% EtOAc in heptane; 1% AcOH.

^{1}H spectrum : (300 MHz, $CDCl_3$) δ 7.83 (d, *J* = 8.3 Hz, 2H), 7.29 (d, *J* = 8.1 Hz, 2H), 2.41 (s, 3H), 2.13 (s, 3H).

^{13}C spectrum : (75 MHz, $CDCl_3$) δ 156.3, 143.8, 141.9, 138.5, 129.7, 126.6, 97.2, 63.3, 21.7, 4.7.

HRMS (ESI): *m/z* calculated for: $C_{12}H_{11}N_3O_3S^+$ $[M+H]^+$ 278,0594, found 278,0588.

(15r):

(*E*)-*N*-(5-cyclopropyl-1,3,4-oxadiazol-2(3H)-ylidene)-4-methoxybenzenesulfonamide:

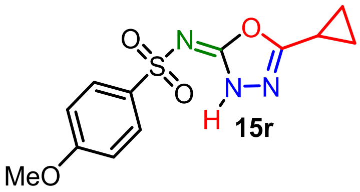

15r

Product **15r** was synthesized according to procedure GP-C and isolated, via column chromatography using the reported TLC system with a gradient from heptane increasing in 5% steps until the reported ethyl acetate concentration, as white solid (74 mg, 0.25 mmol, 50% yield).

TLC System: 30% EtOAc in heptane; 1 % AcOH.

^{1}H spectrum : (300 MHz, $CDCl_3$) δ 7.88 (d, *J* = 8.9 Hz, 2H), 6.94 (d, *J* = 8.9 Hz, 2H), 3.84 (s, 3H), 1.11 (t, *J* = 2.3 Hz, 1H), 1.09 – 1.05 (m, 3H).

^{13}C spectrum : (75 MHz, $CDCl_3$) δ 162.9, 160.2, 156.7, 133.5, 128.6, 114.1, 55.7, 7.7, 6.4.

HRMS (ESI): m/z calculated for: $C_{12}H_{13}N_3O_4S^+$ $[M+H]^+$ 296.0700, found 296.0694.

(15s):

(*E*)-*N*-(5-(tert-butyl)-1,3,4-oxadiazol-2(3H)-ylidene)naphthalene-2-sulfonamide:

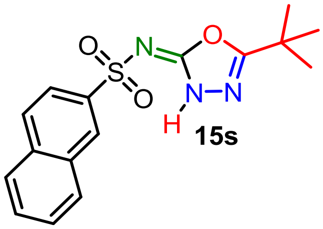

15s

Product **15s** was synthesized according to procedure GP-D and isolated, via column chromatography using the reported TLC system with a gradient from heptane increasing in 5% steps until the reported ethyl acetate concentration, as white solid in (102 mg, 0.31 mmol, 62% yield).

TLC System: 20% EtOAc in heptane; 1% AcOH.

^{1}H spectrum : (300 MHz, $CDCl_3$) δ 8.54 (s, 1H), 8.02 – 7.94 (m, 3H), 7.92 – 7.87 (m, 2H), 7.68 – 7.53 (m, 2H), 1.33 (s, 9H).

^{13}C spectrum : (75 MHz, $CDCl_3$) δ 165.3, 157.3, 138.4, 134.9, 132.1, 129.3, 129.3, 128.7, 127.9, 127.4, 127.3, 122.2, 32.5, 27.2.

HRMS (ESI): *m/z* calculated for: $C_{16}H_{17}N_3O_3S^+$ $[M+H]^+$ 332.1063, found 332.1052.

(15t):

(*E*)-*N*-(5-cyclopropyl-1,3,4-oxadiazol-2(3H)-ylidene)-3-methylbenzenesulfonamide:

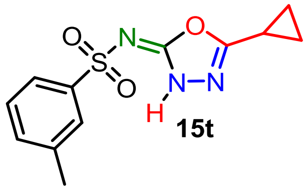

15t

Product **15t** was synthesized according to procedure GP-C and isolated, via column chromatography using the reported TLC system with a gradient from heptane increasing in 5% steps until the reported ethyl acetate concentration, as white solid (64 mg, 0.23 mmol, 46% yield).

TLC System: 30% EtOAc in heptane; 1% AcOH.

^{1}H spectrum : (300 MHz, $CDCl_3$) δ 7.81 – 7.69 (m, 2H), 7.39 – 7.34 (m, 2H), 2.40 (s, 3H), 2.06 – 1.84 (m, 1H), 1.19 – 1.00 (m, 4H).

^{13}C spectrum : (75 MHz, $CDCl_3$) δ 160.2, 156.7, 141.3, 139.1, 133.4, 128.8, 126.8, 123.5, 21.3, 7.7, 6.3.

HRMS (ESI): *m/z* calculated for: $C_{12}H_{13}O_3N_3S$+ [M+H]+ 280.0750, found 280.0750.

(15u):

(*E*)-3-bromo-*N*-(5-cyclopropyl-1,3,4-oxadiazol-2(3H)-ylidene)benzenesulfonamide:

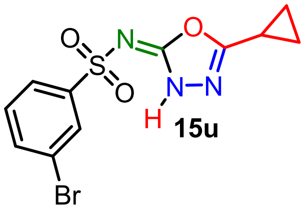

15u

Product **15u** was synthesized according to procedure GP-C and isolated, via column chromatography using the reported TLC system with a gradient from heptane increasing in 5% steps until the reported ethyl acetate concentration, as white solid (110 mg, 0.32 mmol, 64% yield).

TLC System: 30% EtOAc in heptane; 1% AcOH.

^{1}H spectrum : (300 MHz, $CDCl_3$) δ 8.00 (s, 1H), 7.78 (dd, *J* = 7.9, 0.9 Hz, 1H), 7.54 (dd, *J* = 8.0, 1.0 Hz, 1H), 7.24 (t, *J* = 7.9 Hz, 1H), 1.88 – 1.73 (m, 1H), 1.04 – 0.87 (m, 4H).

^{13}C spectrum : (75 MHz, $CDCl_3$) δ 160.3, 156.2, 143.9, 134.9, 130.2, 129.4, 124.9, 122.4, 7.5, 6.1.

HRMS (ESI): *m/z* calculated for: $C_{11}H_{10}BrN_3O_3S^+$ [M+H]$^+$ 343.9699, found 343.9685.

(15v):

(*E*)-4-bromo-*N*-(5-(tert-butyl)-1,3,4-oxadiazol-2(3H)-ylidene)benzenesulfonamide:

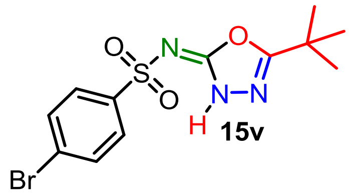

Product **15v** was synthesized according to procedure GP-D and isolated, via column chromatography using the reported TLC system with a gradient from heptane increasing in 5% steps until the reported ethyl acetate concentration, as white solid (126 mg, 0.35 mmol, 70% yield).

TLC system: 40% EtOAc in heptane; 1% AcOH.

^{1}H spectrum : (300 MHz, $CDCl_3$) δ 7.83 (d, *J* = 8.6 Hz, 2H), 7.65 – 7.56 (m, 2H), 1.33 (s, 9H).

^{13}C spectrum : (75 MHz, $CDCl_3$) δ 165.8, 140.8, 132.2, 128.2, 127.5, 66.0, 32.6, 27.4.

HRMS (ESI): *m/z* calculated for: $C_{12}H_{14}BrN_3O_3S^+$ $[M+H]^+$ 360.0012, found 360.0025.

(15w):

(*E*)-*N*-(5-(but-3-en-1-yl)-1,3,4-oxadiazol-2(3H)-ylidene)propane-1-sulfonamide:

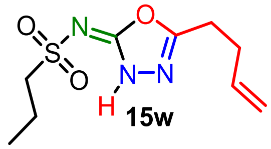

Product **15w** was synthesized according to procedure GP-C and isolated, via column chromatography using the reported TLC system with a gradient from heptane increasing in 5% steps until the reported ethyl acetate concentration, as a clear sticky oil, that solidifies in the freezer, (37 mg, 0.15 mmol, 30% yield).

TLC System: 30% EtOAc in heptane; 1% AcOH.

^{1}H spectrum : (300 MHz, $CDCl_3$) δ 5.81 (ddt, *J* = 16.9, 10.3, 6.5 Hz, 1H), 5.21 – 5.02 (m, 2H), 3.19 – 3.05 (m, 2H), 2.78 (t, *J* = 7.4 Hz, 2H), 2.49 (q, *J* = 7.1 Hz, 2H), 1.90 (tdd, *J* = 7.7, 6.7, 2.8 Hz, 2H), 1.07 (td, *J* = 7.5, 5.6 Hz, 3H).

^{13}C spectrum : (75 MHz, $CDCl_3$) δ 134.9, 117.3, 57.1, 56.4, 29.3, 25.1, 17.8, 17.4, 13.0.

HRMS (ESI): *m/z* calculated for: $C_9H_{15}N_3O_3S^+$ $[M+Z]^+$ 246.0907, found 246.0908.

(15x):

(*E*)-*N*-(5-cyclopropyl-1,3,4-oxadiazol-2(3H)-ylidene)-1-phenylmethanesulfonamide:

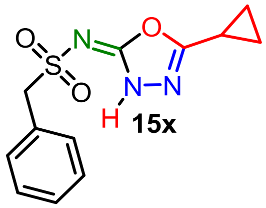

15x

Product **15x** was synthesized according to procedure GP-C and isolated, via column chromatography using the reported TLC system with a gradient from heptane increasing in 5% steps until the reported ethyl acetate concentration, as white solid (100 mg, 0.36 mmol, 72% yield).

TLC System: 40% EtOAc in heptane; 1% AcOH.

^{1}H spectrum : (300 MHz, $CDCl_3$) δ 9.92 (s, 1H), 7.42 (dd, *J* = 6.7, 3.0 Hz, 2H), 7.38 – 7.23 (m, 3H), 4.34 (s, 2H), 1.95 – 1.80 (m, 1H), 1.17 – 0.97 (m, 4H).

^{13}C spectrum : (75 MHz, $CDCl_3$) δ 159.8, 157.7, 130.9, 129.2, 128.7, 128.5, 60.4, 7.7, 6.2.

HRMS (ESI): *m/z* calculated for: $C_{12}H_{13}N_3O_3S^+$ $[M+Na]^+$ 302.0569 found 302.0565.

(15y):

***N*-((*E*)-5-((R)-3-((5R,8R,9S,10S,13R,14S,17R)-10,13-dimethyl-3-oxohexadecahydro-1H-cyclopenta[a]phenanthren-17-yl)butyl)-1,3,4-oxadiazol-2(3H)-ylidene)-4-methylbenzenesulfonamide:**

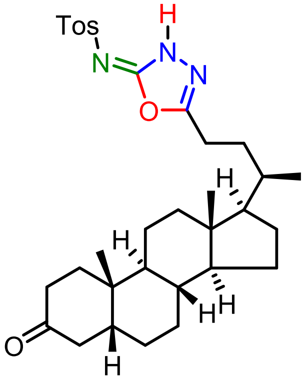

15y

Product **15y** was synthesized according to procedure GP-C and isolated, via column chromatography using the reported TLC system with a gradient from heptane increasing in 5% steps until the reported ethyl acetate concentration, as white solid (62 mg, 0.105 mmol, 42% yield).

TLC System: 20% EtOAc in heptane; 1% AcOH.

^{1}**H spectrum :** (300 MHz, $CDCl_3$) δ 7.81 (d, *J* = 8.3 Hz, 2H), 7.28 – 7.23 (d, *J* = 8.3 Hz, 2H), 3.02 – 2.77 (m, 3H), 2.77 – 2.49 (m, 2H), 2.39 (s, 4H), 2.35 – 2.08 (m, 8H), 2.08 – 1.76 (m, 8H), 1.69 – 1.42 (m, 2H), 1.39 (s, 3H), 1.30 – 1.22 (m, 3H), 1.05 (s, 3H), 0.86 (d, *J* = 6.6 Hz, 3H).

^{13}**C spectrum :** (75 MHz, $CDCl_3$) δ 212.2, 209.4, 208.9, 159.6, 143.4, 138.9, 129.6, 126.5, 56.9, 51.9, 49.0, 46.9, 45.6, 45.5, 45.1, 42.9, 38.7, 36.6, 36.1, 35.5, 35.4, 31.2, 27.8, 25.2, 22.8, 21.9, 21.6, 18.6, 11.9.

HRMS (ESI): *m/z* calculated for: $C_{32}H_{41}N_3O_6S$+ [M+H]+ 596.2789, found 596.2813.

(17g):

5-cyclopropyl-1,3,4-oxadiazol-2-amine:

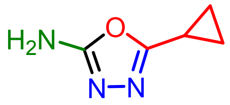

17g

Product **17g** was synthesized according to procedure GP-E and isolated as white solid (9 mg, 0.072 mmol, 72% yield) without further purification techniques required and the spectral data agreed with literature.[4]

TLC System: 100% EtOAc; 1% triethylamine.

^{1}**H spectrum :** (300 MHz, DMSO-d_6) δ 6.79 (s, 2H), 2.04 – 1.92 (m, 1H), 1.01 – 0.92 (m, 2H), 0.86 – 0.78 (m, 2H).

^{1}**H spectrum :** (300 MHz, Chloroform-d) δ 4.82 (s, 2H), 2.04 – 1.93 (m, 1H), 1.10 – 0.96 (m, 4H).

^{1}H spectrum 15a (300 MHz, $CDCl_3$, DMSO)

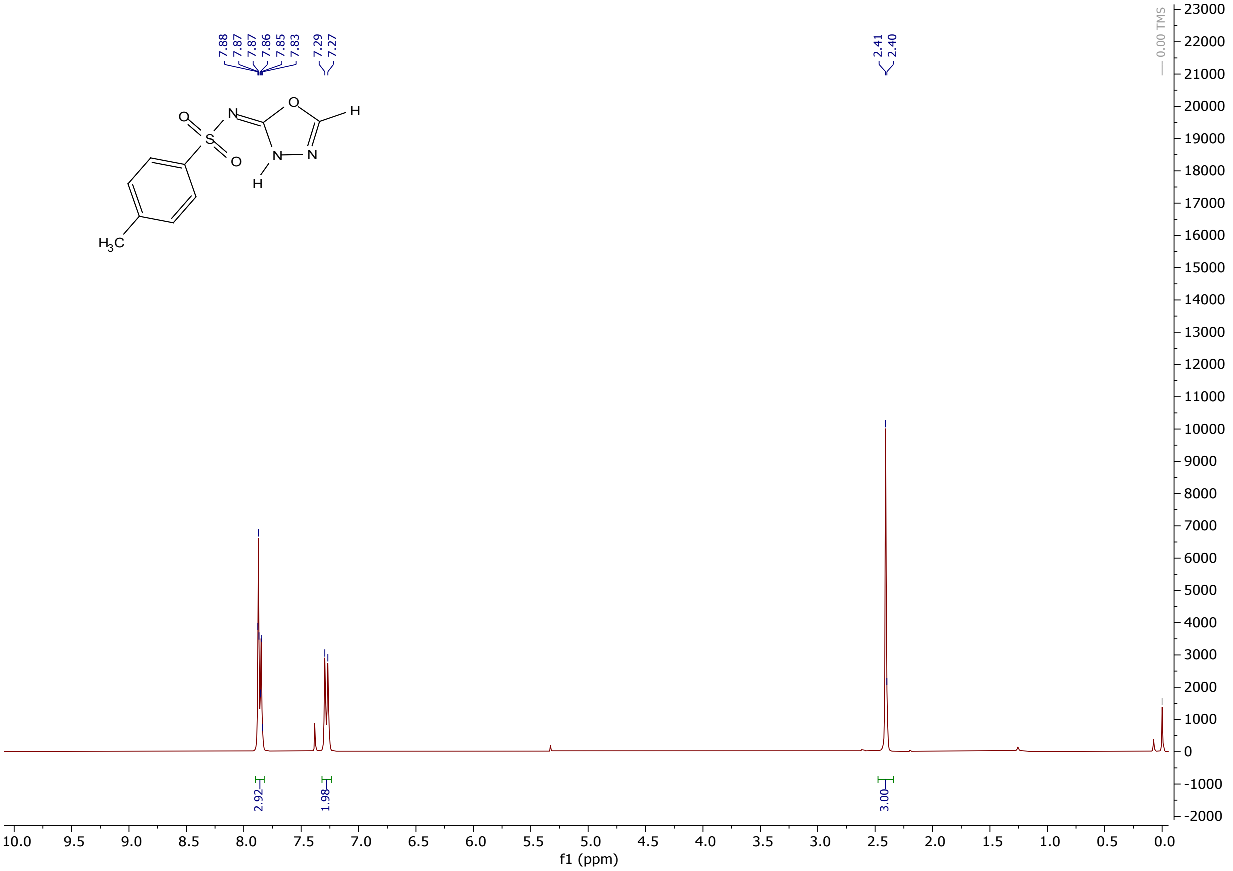

^{13}C spectrum <u>15a</u> (75 MHz, CDCl3, DMSO)

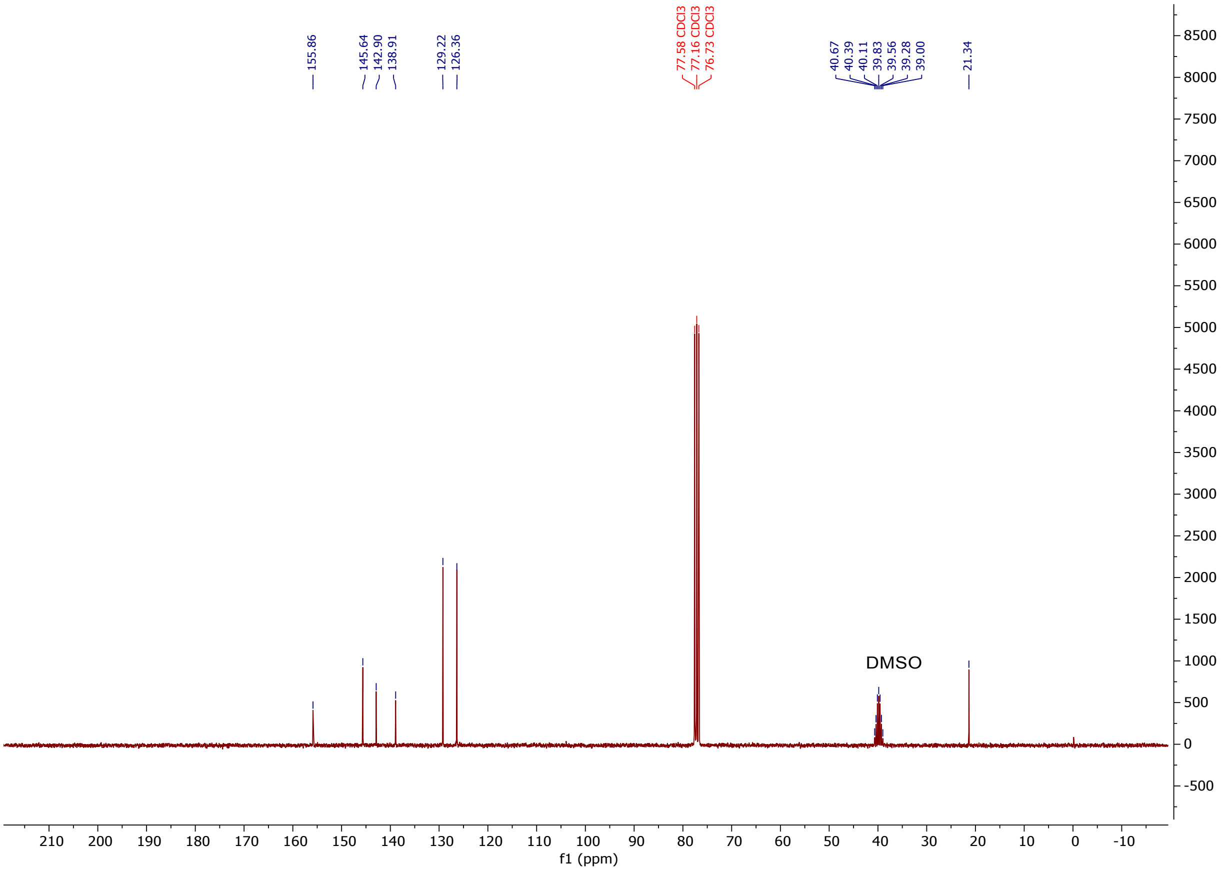

^{1}H spectrum <u>15b</u> (300 MHz, $CDCl_3$)

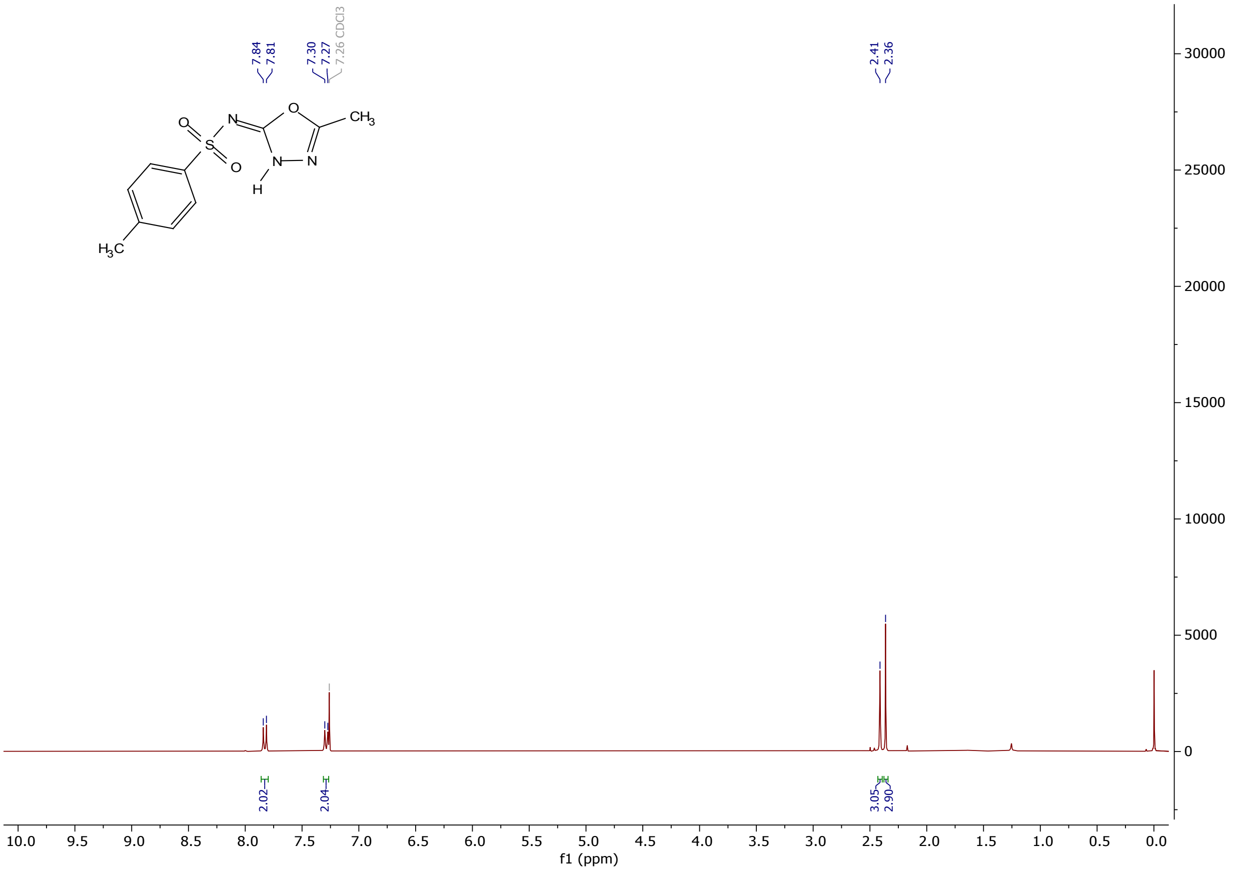

^{13}C spectrum 15b (75 MHz, $CDCl_3$)

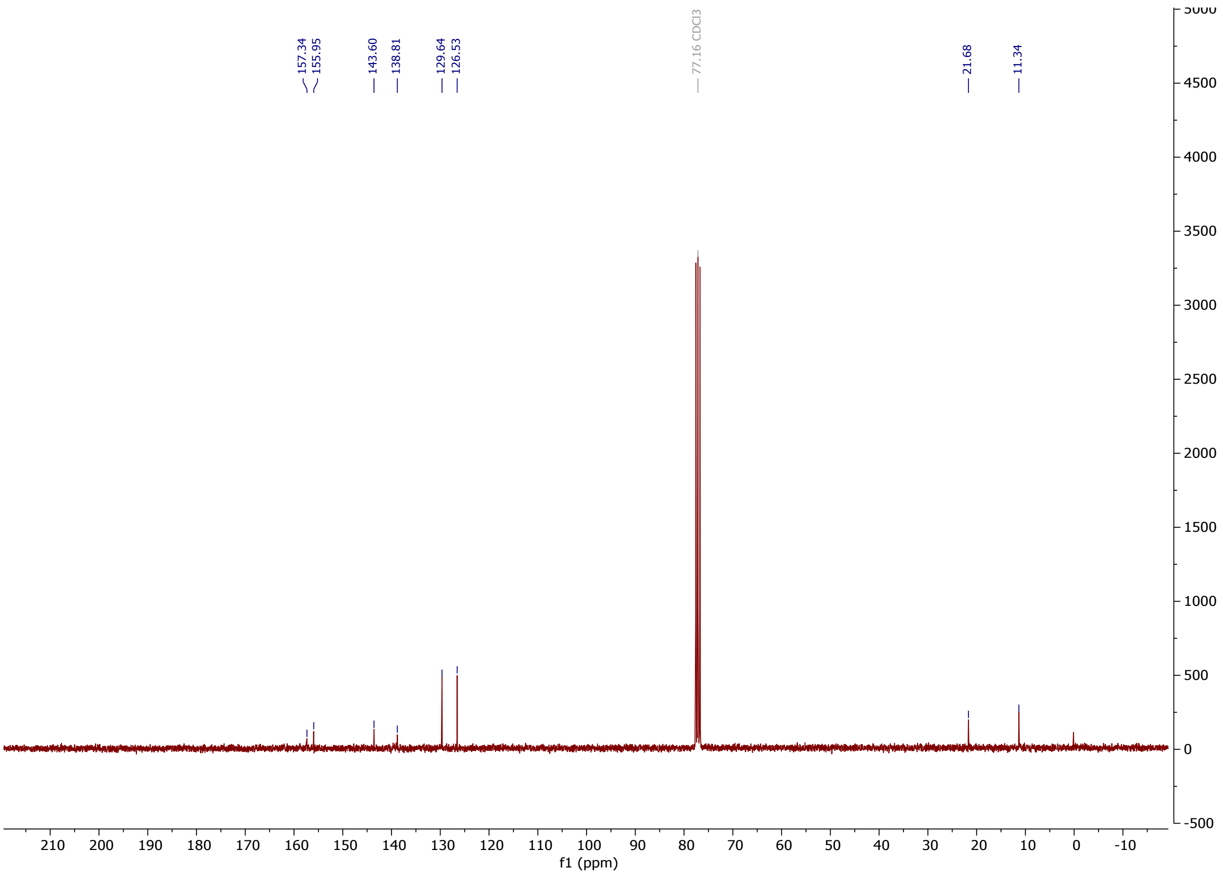

^{1}H spectrum <u>15c</u> (300 MHz, $CDCl_3$)

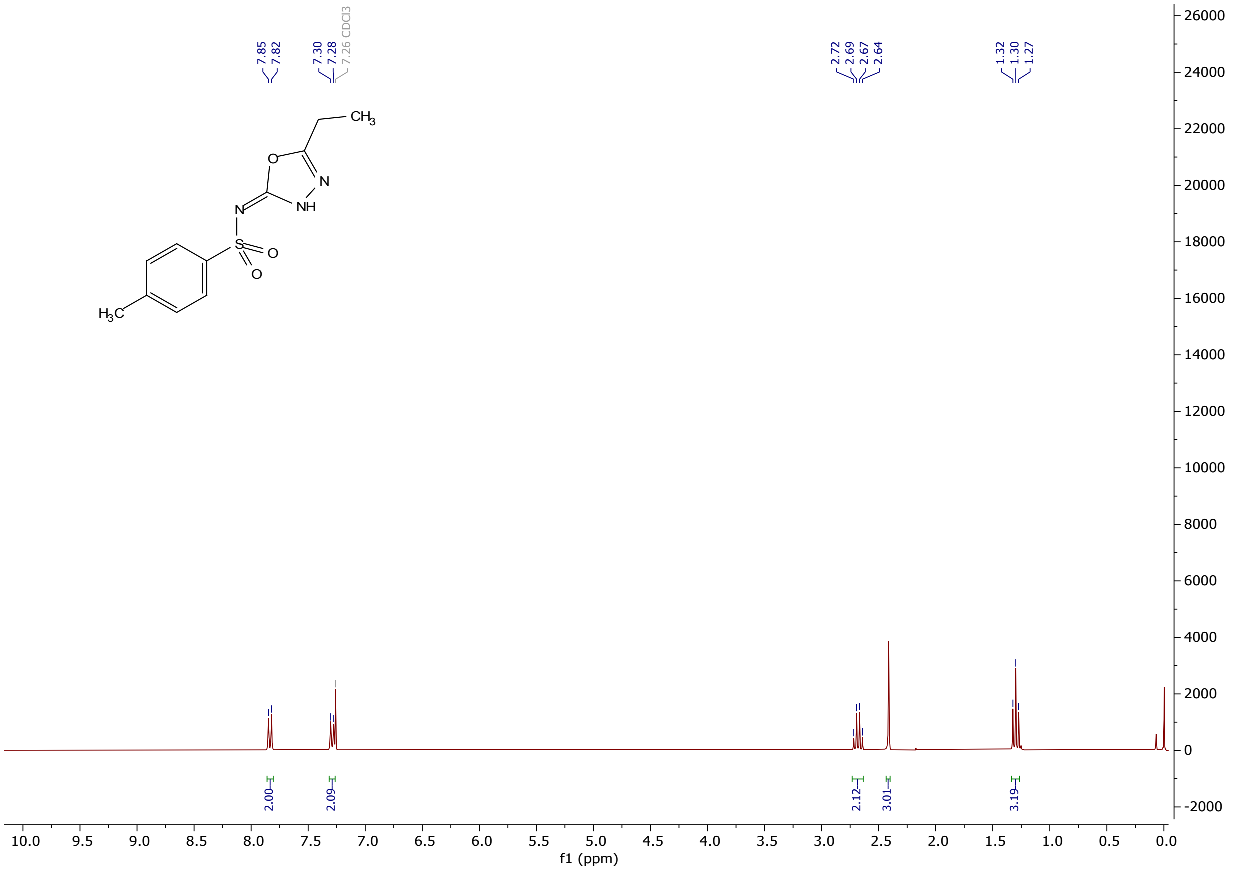

^{13}C spectrum <u>15c</u> (75 MHz, $CDCl_3$)

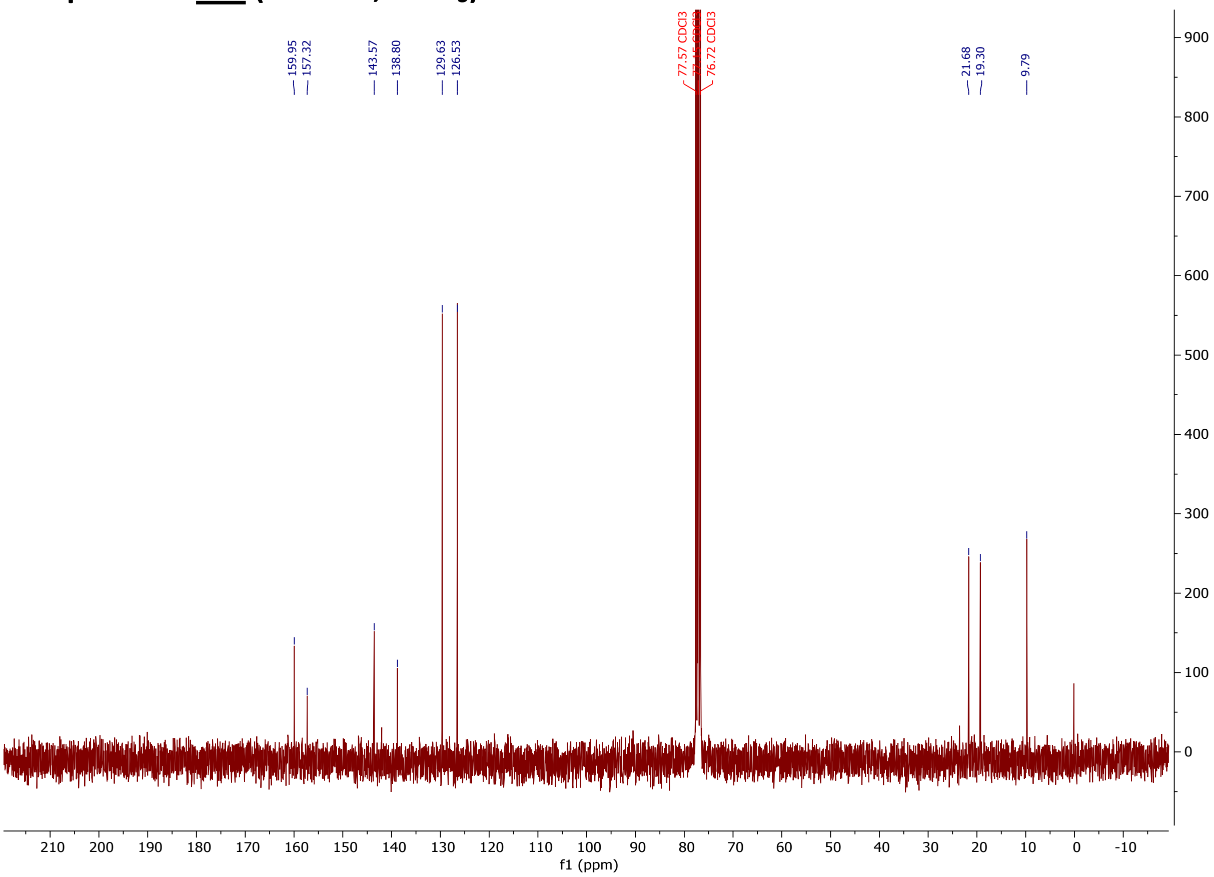

^{1}H spectrum 15d (300 MHz, CDCl3)

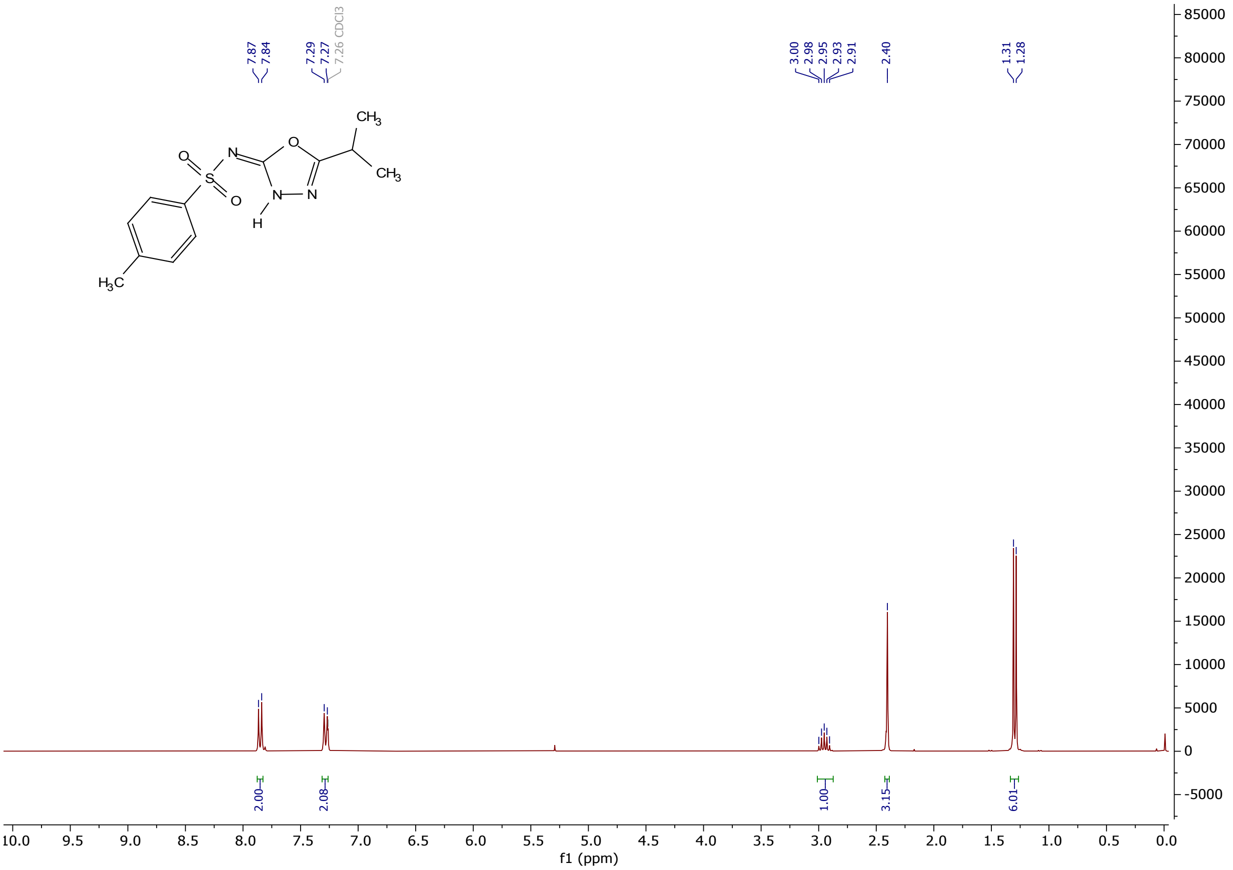

^{13}C spectrum <u>15d</u> (75 MHz, $CDCl_3$)

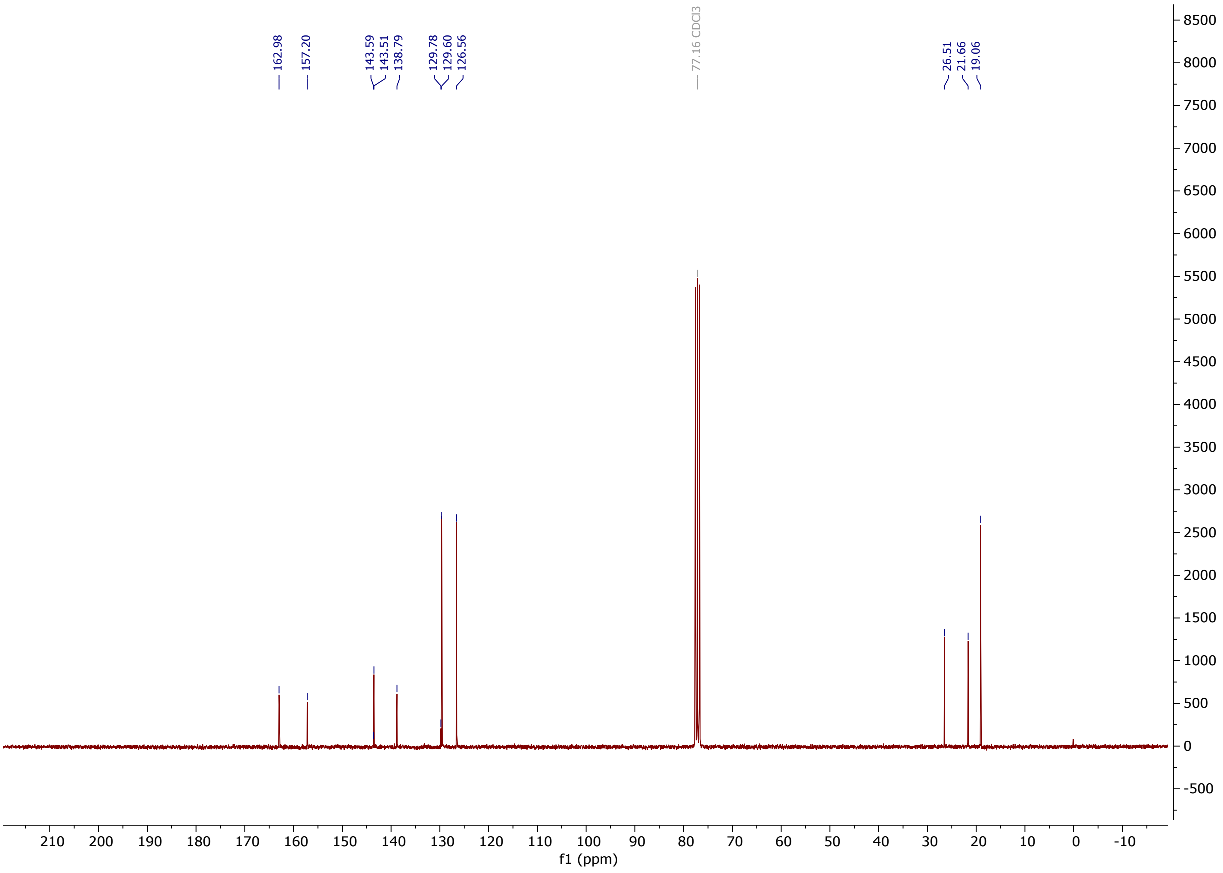

^{1}H spectrum <u>15e</u> (300 MHz, $CDCl_3$)

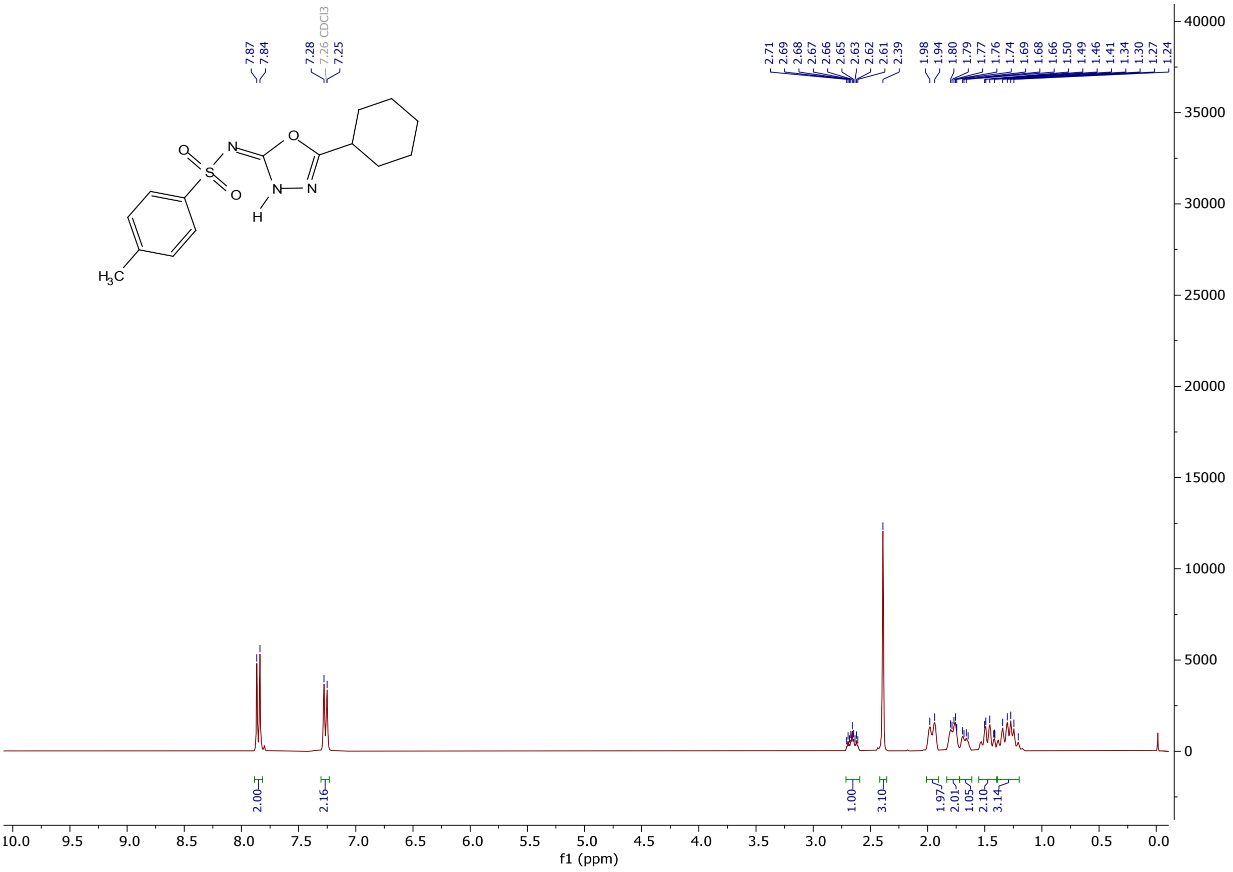

^{13}C spectrum 15e (75 MHz, $CDCl_3$)

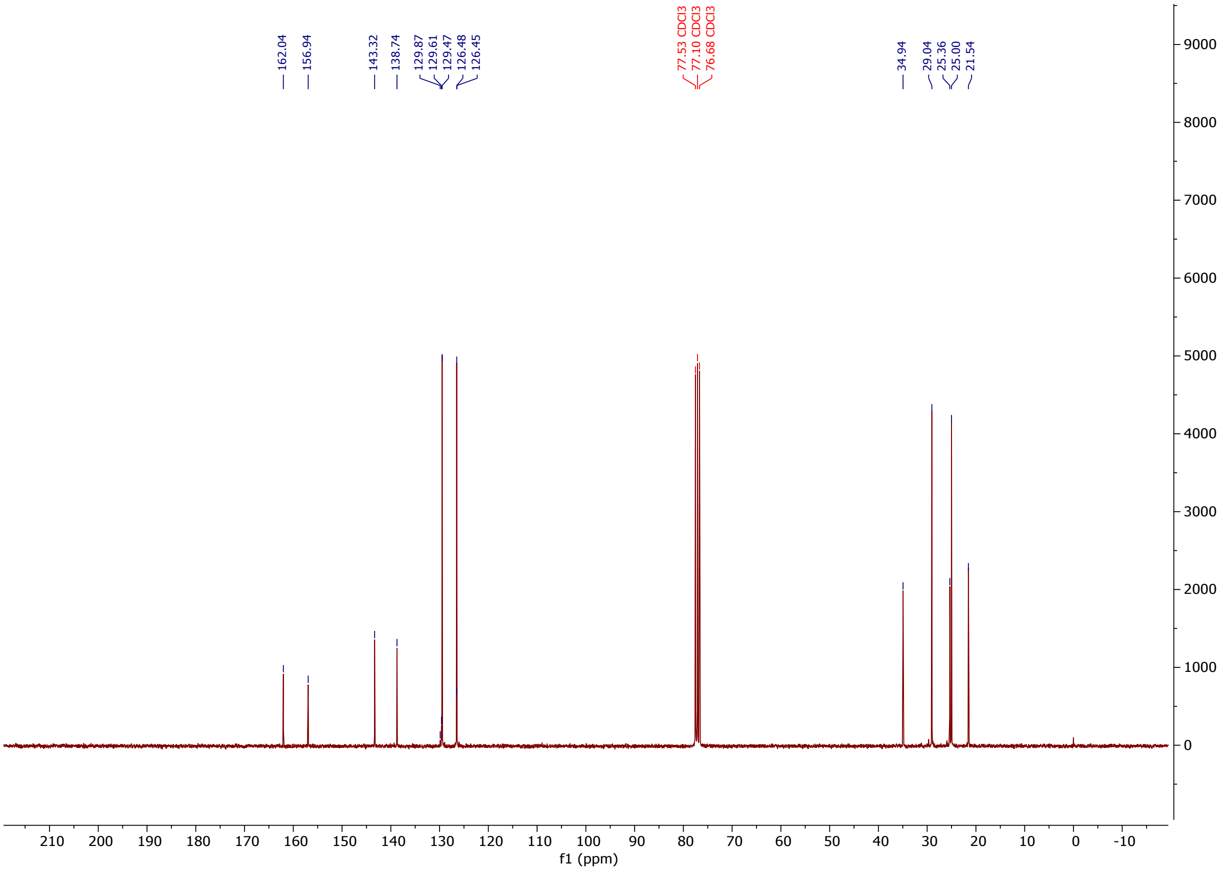

^{1}H spectrum 15f (300 MHz, $CDCl_3$)

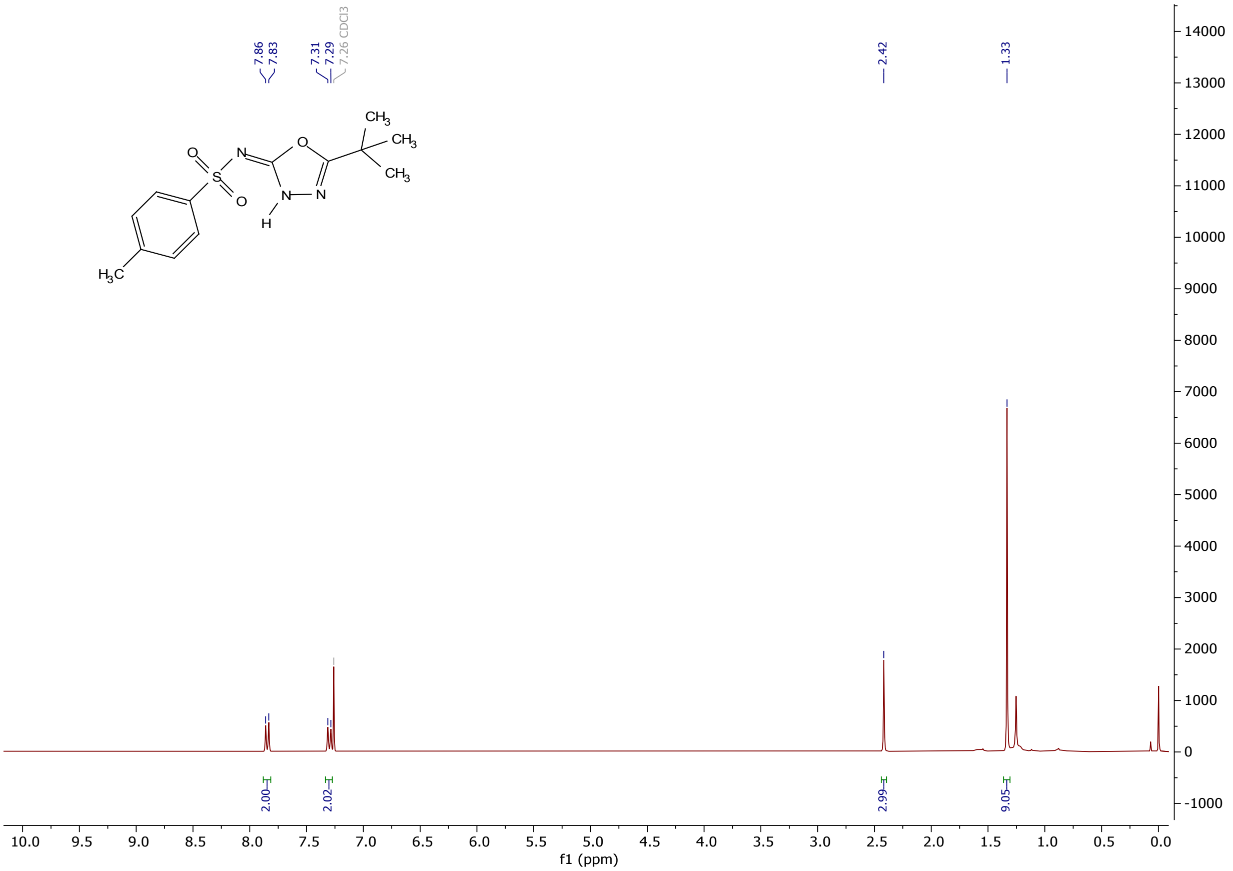

^{13}C spectrum <u>15f</u> (75 MHz, $CDCl_3$)

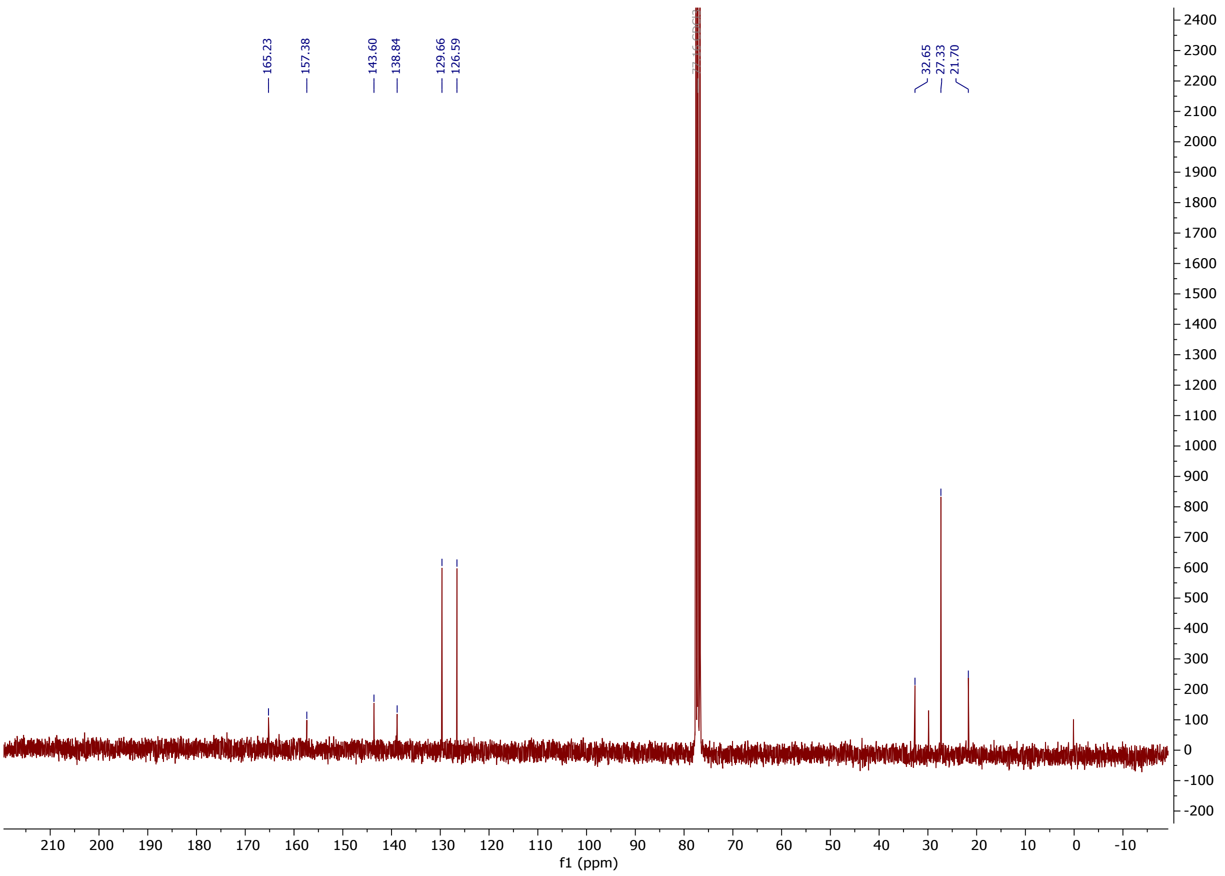

^1H spectrum 15g (300 MHz, $CDCl_3$)

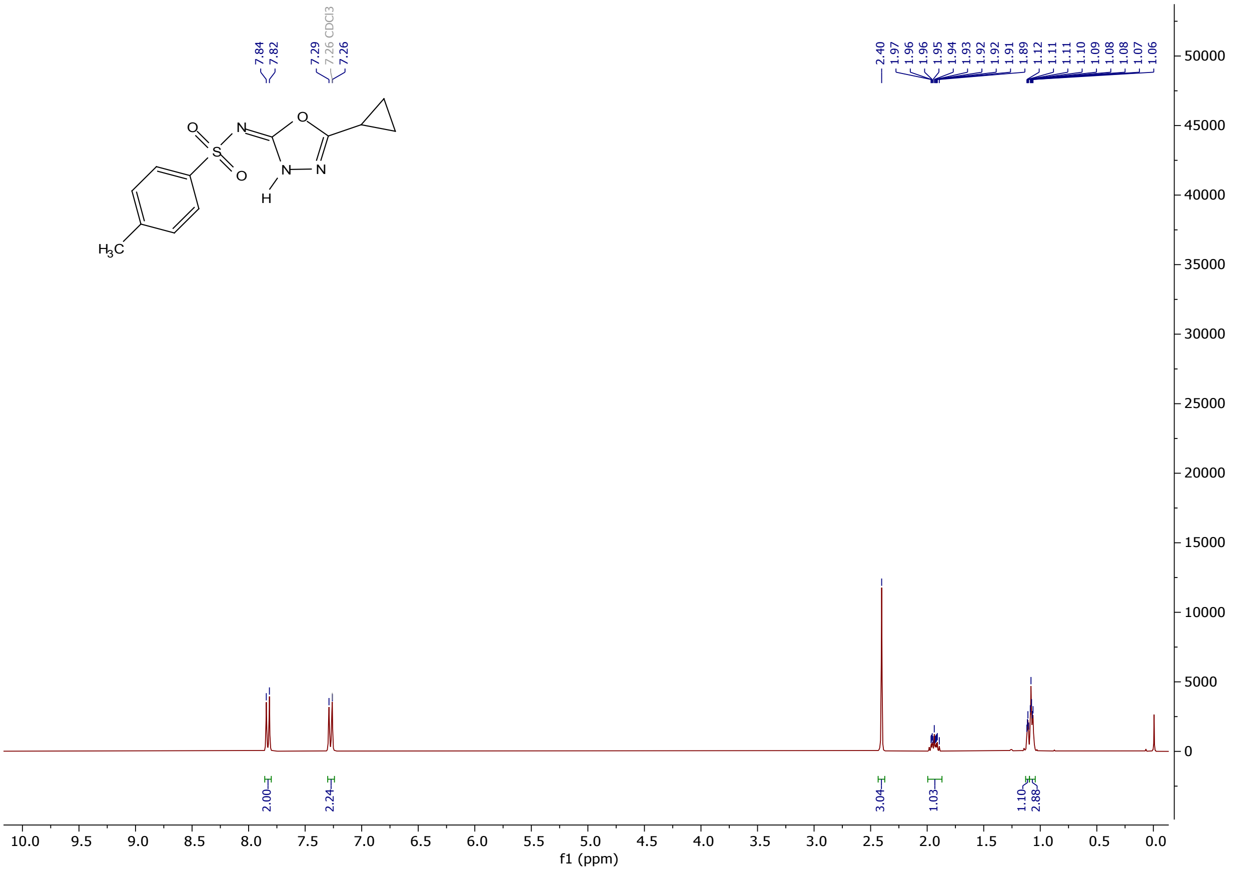

^{13}C spectrum 15g (75 MHz, $CDCl_3$)

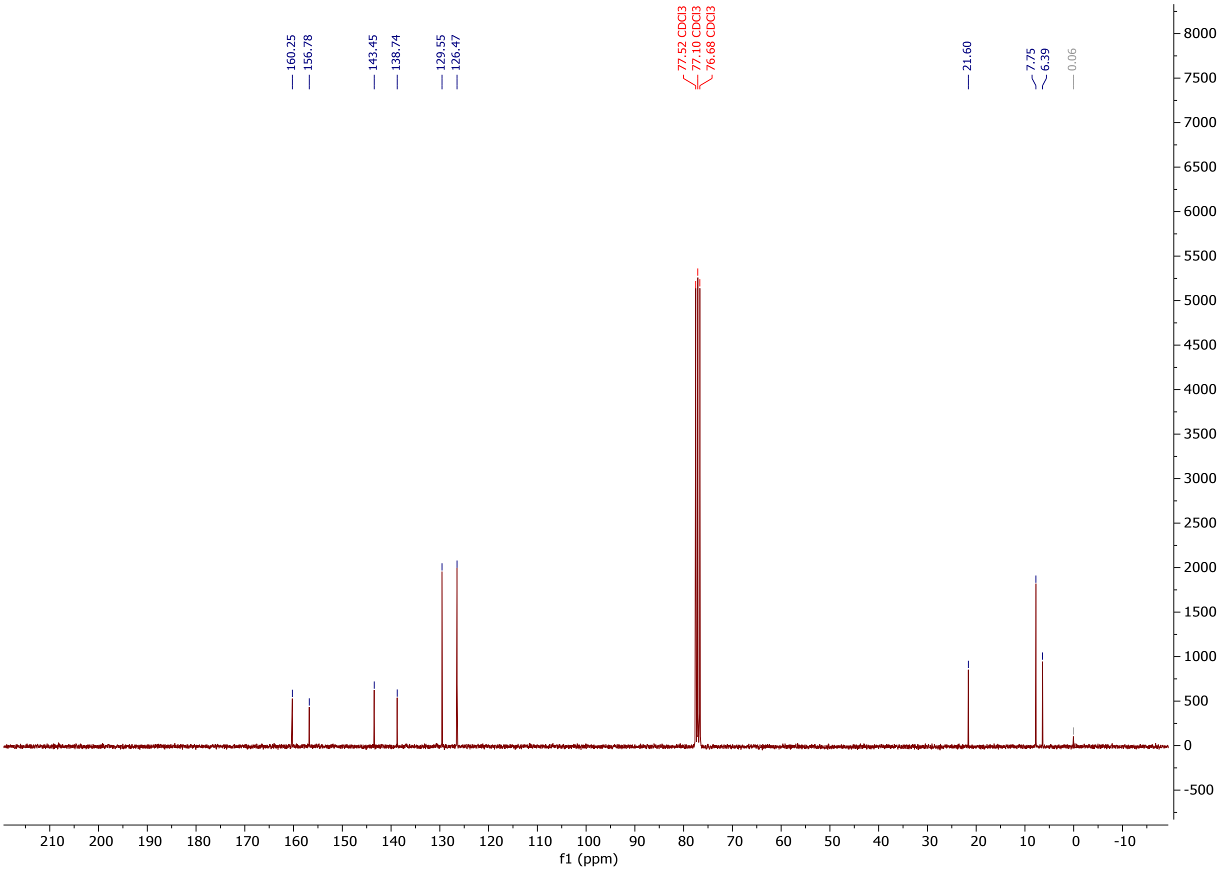

^{1}H spectrum 15h (300 MHz, $CDCl_3$)

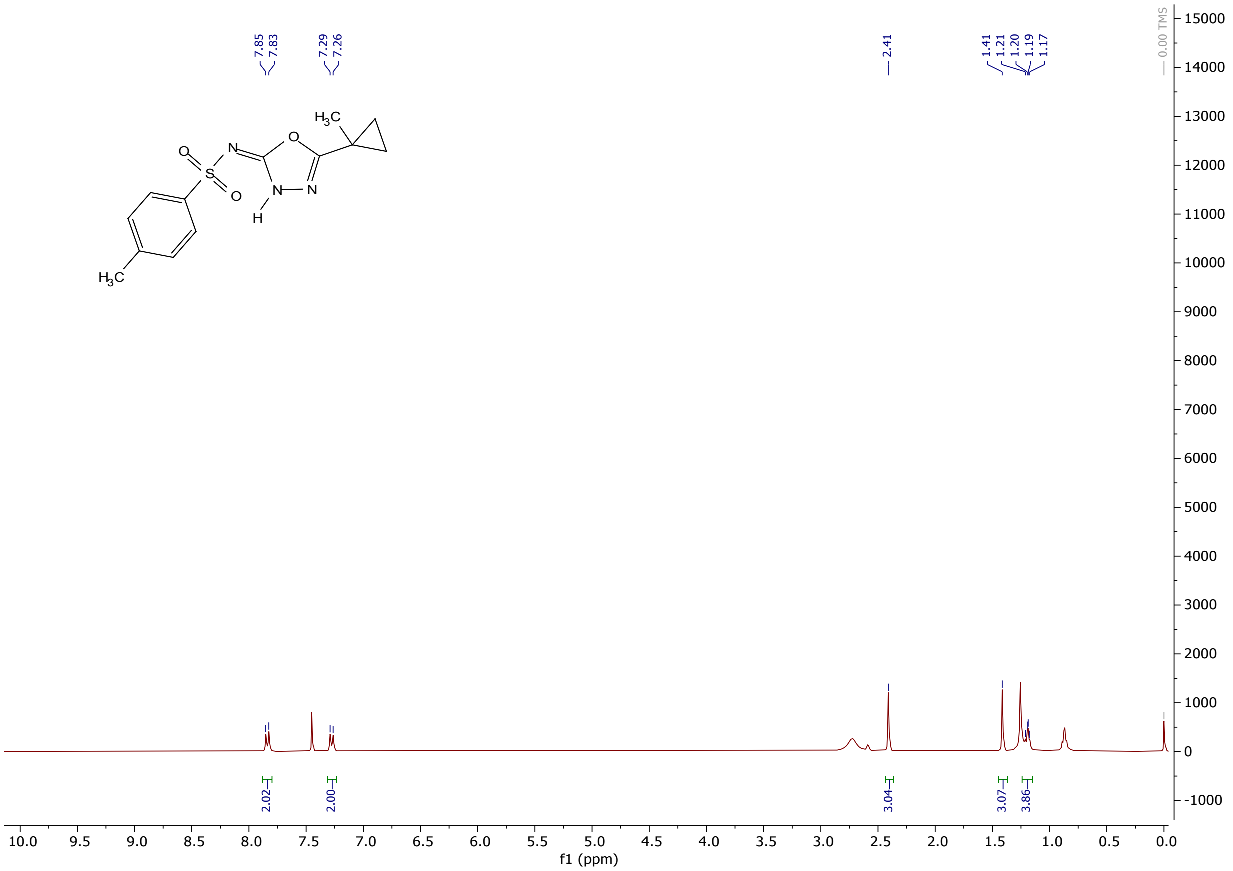

^{13}C spectrum 15h (75 MHz, $CDCl_3$)

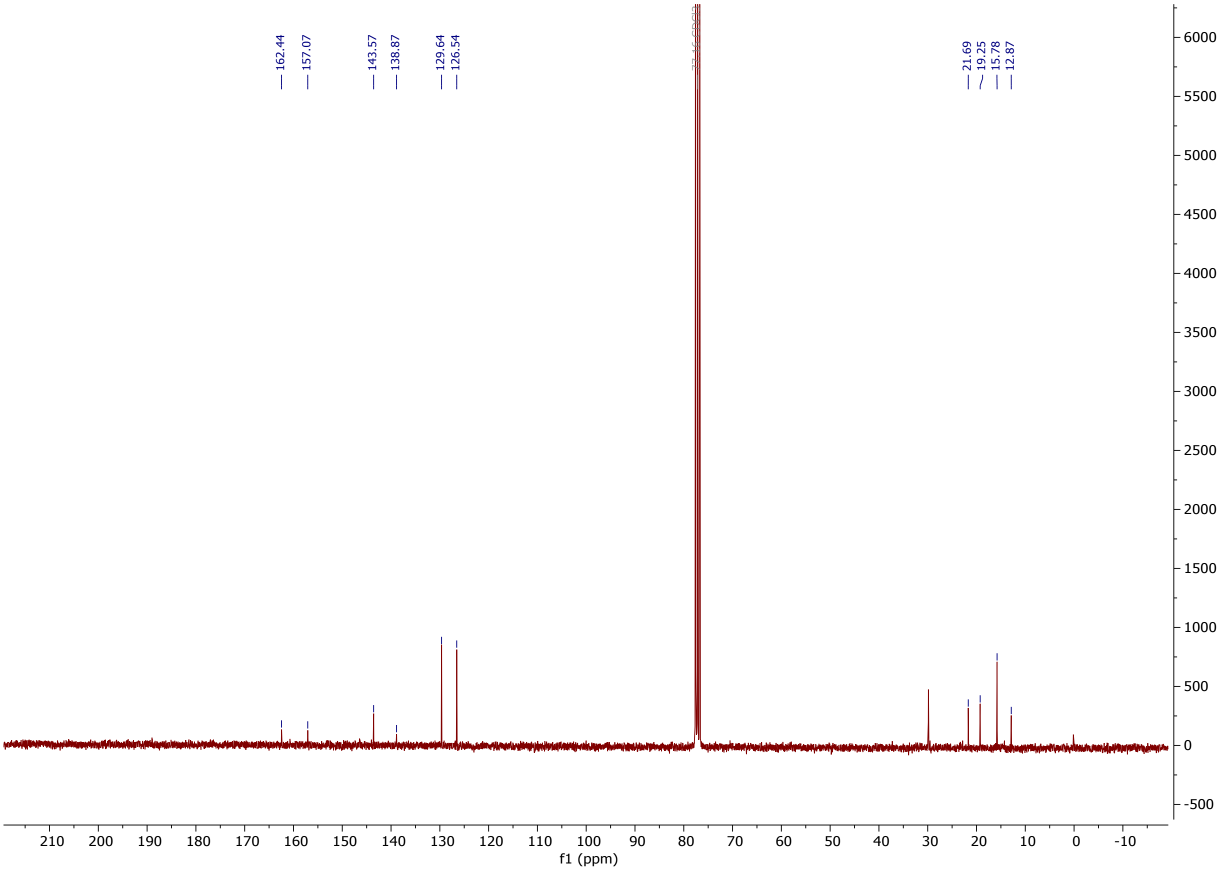

^{1}H spectrum 15i (300 MHz, $CDCl_3$)

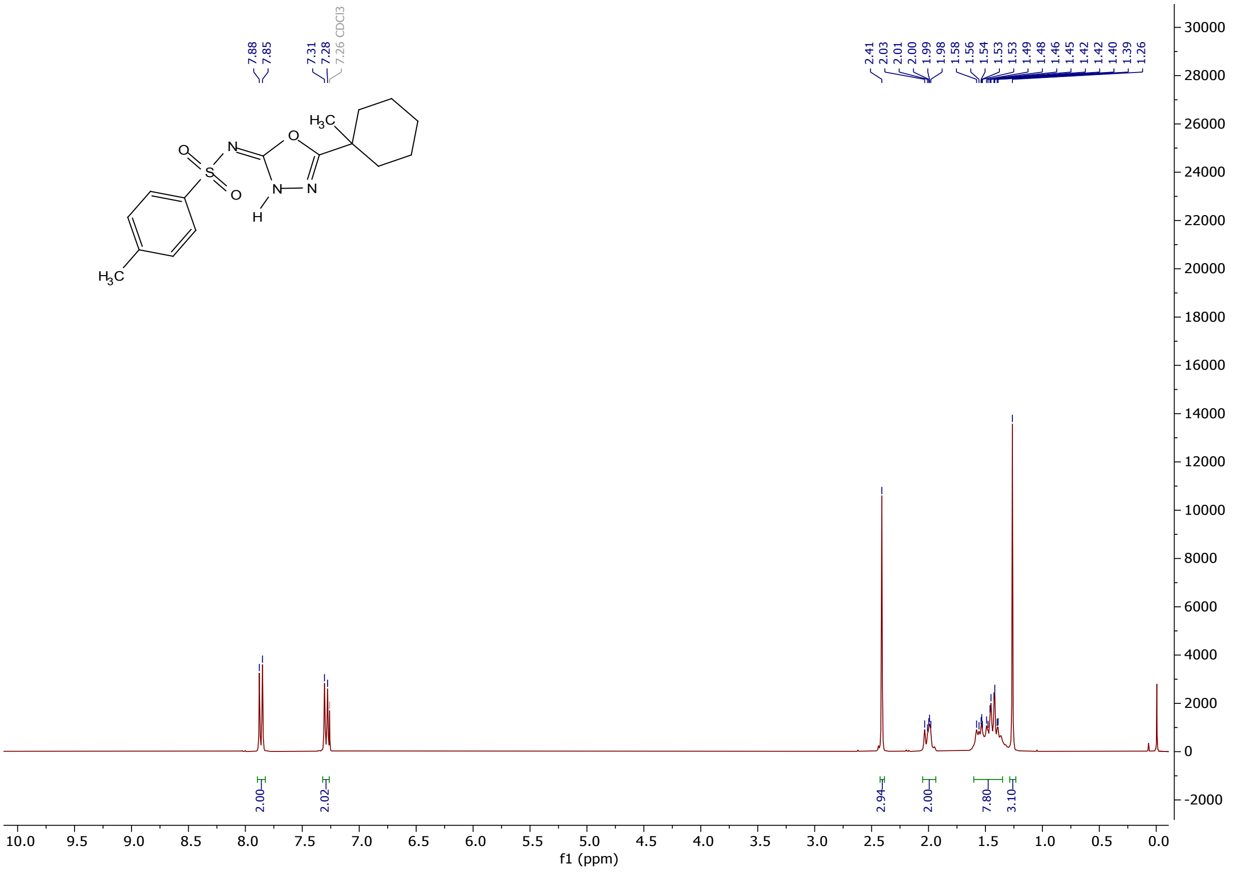

^{13}C spectrum <u>15i</u> (75 MHz, $CDCl_3$)

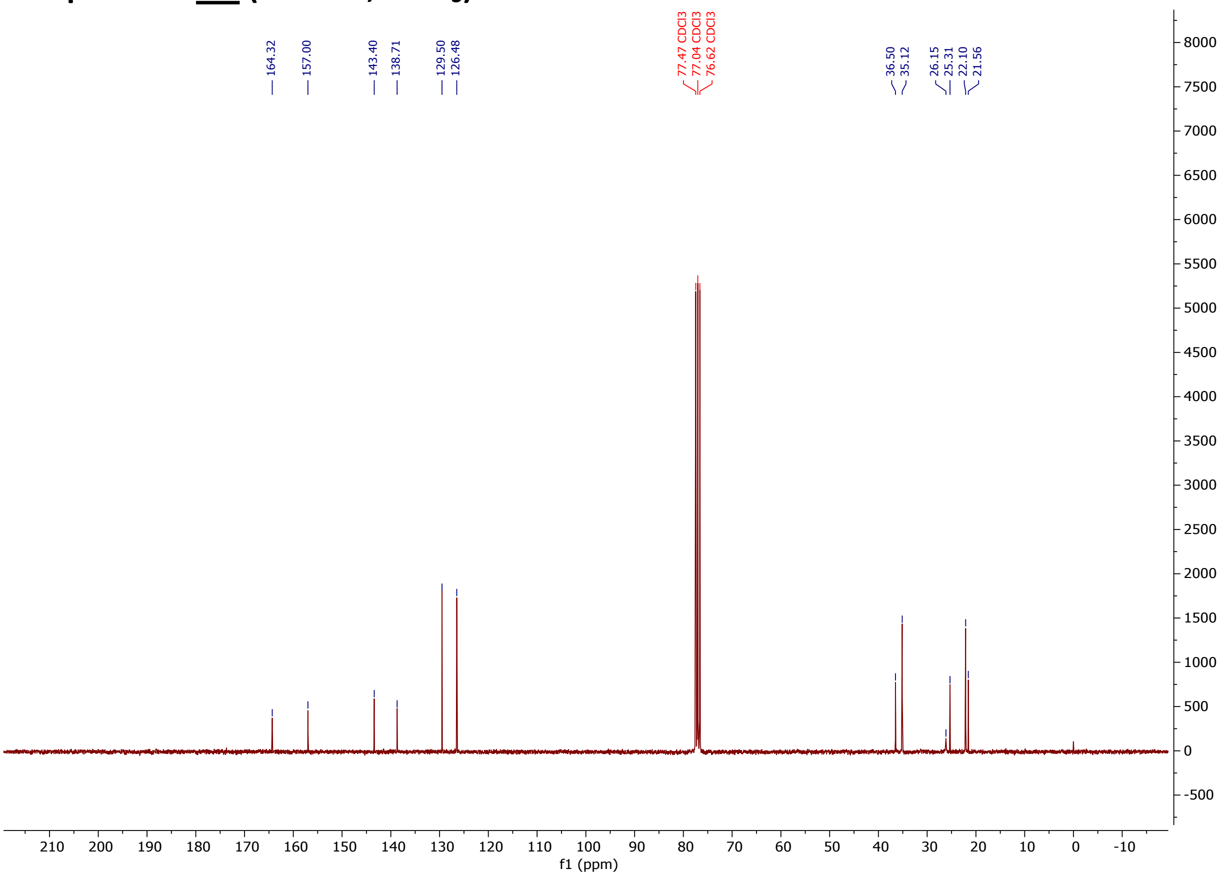

^{1}H spectrum 15j (300 MHz, $CDCl_3$)

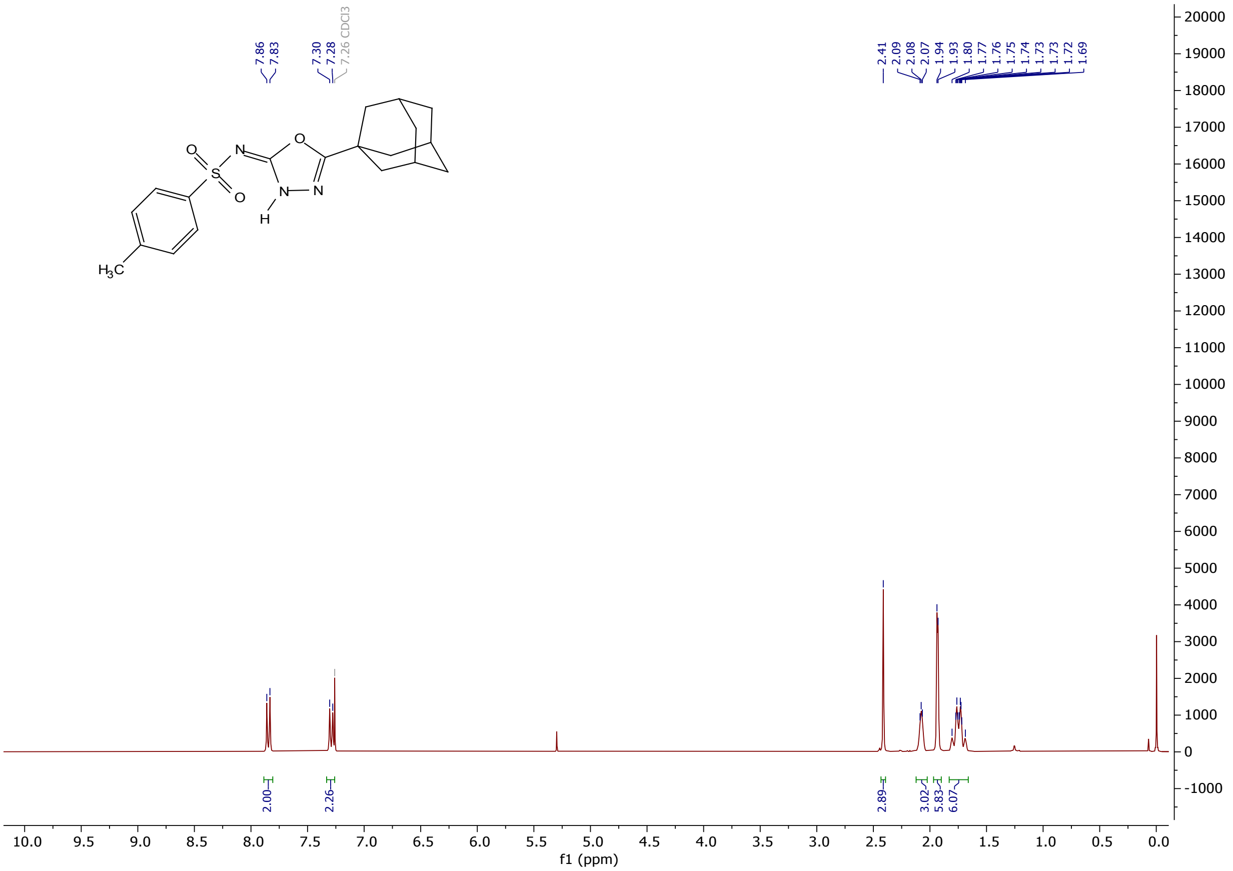

^{13}C spectrum 15j (75 MHz, $CDCl_3$)

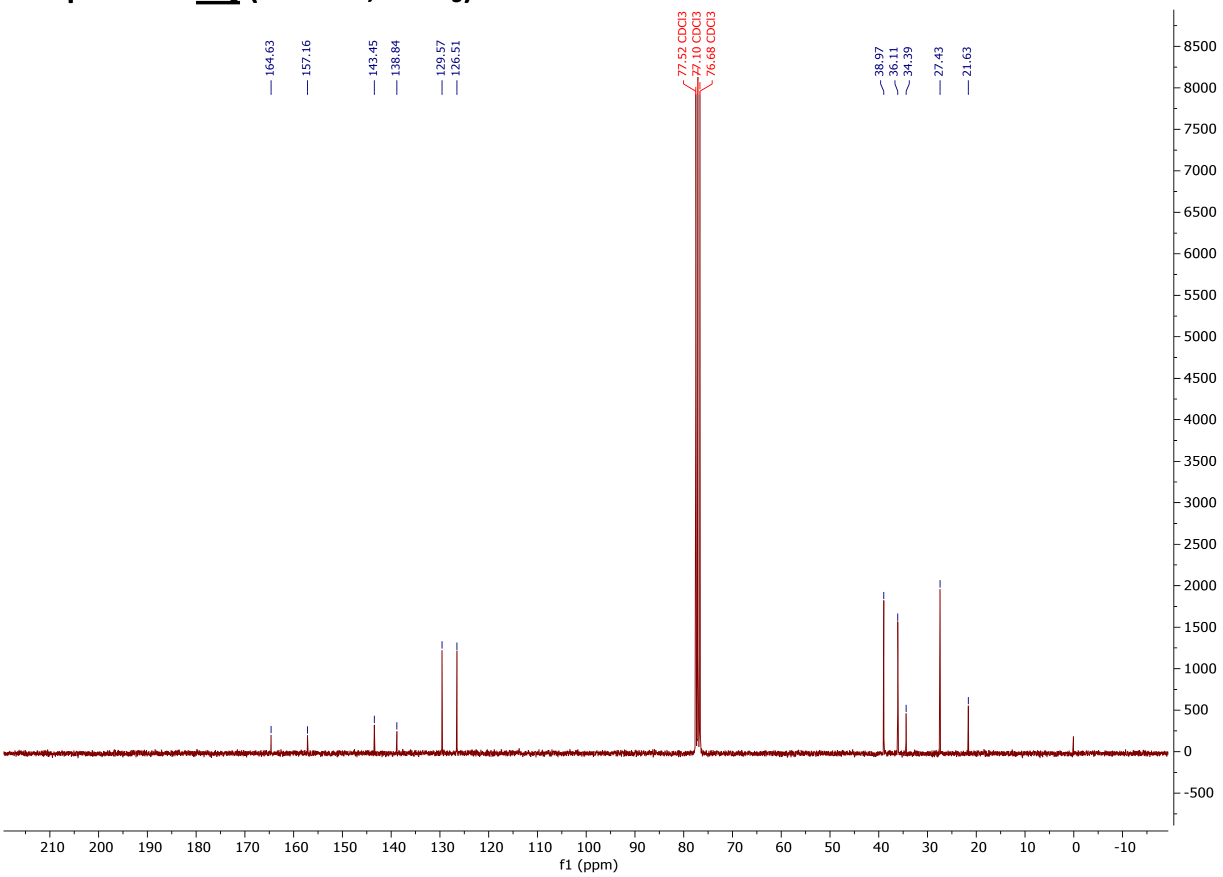

^{1}H spectrum 15k (300 MHz, $CDCl_3$, DMSO)

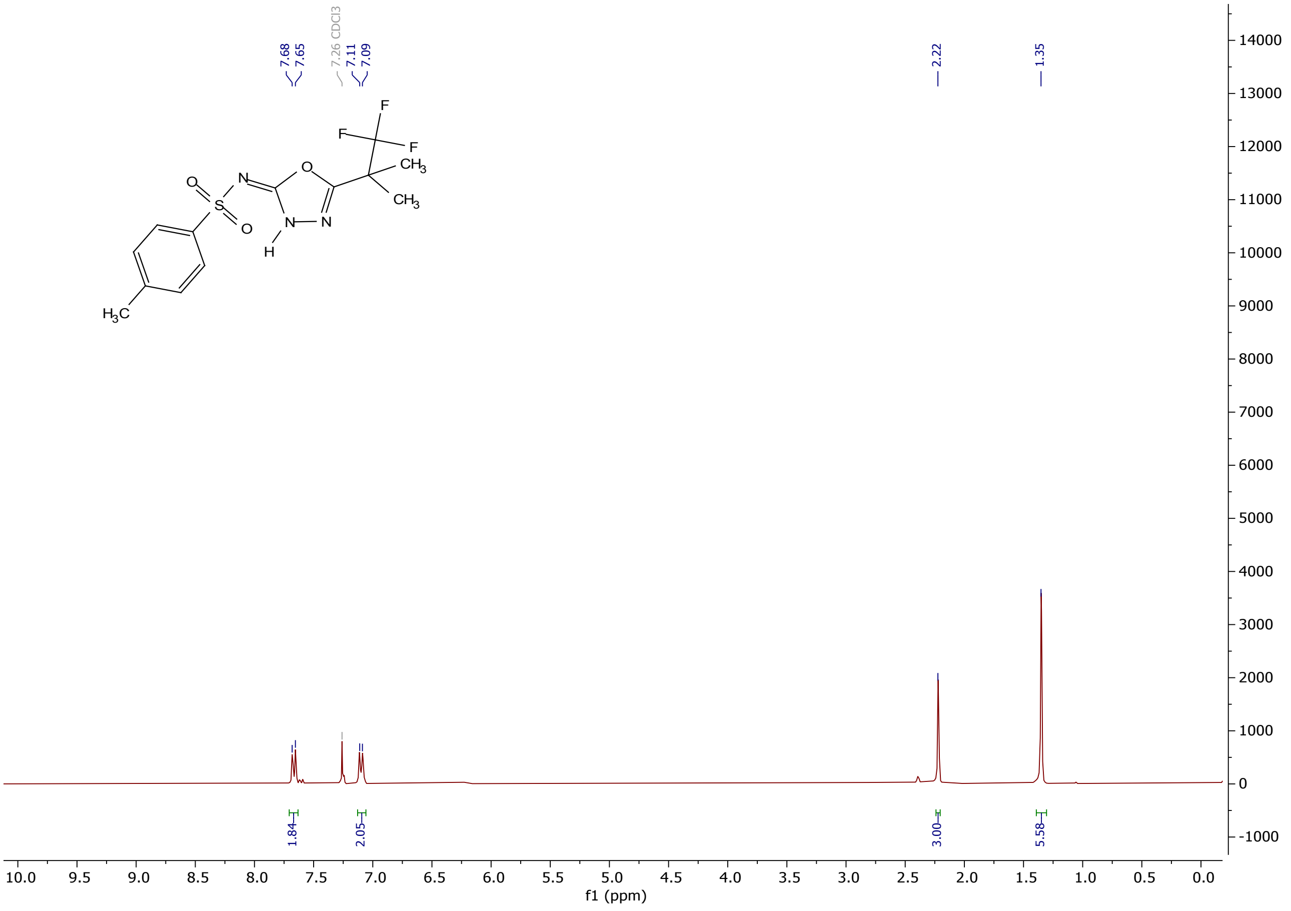

^{19}F spectrum 15k (282 MHz, $CDCl_3$, DMSO)

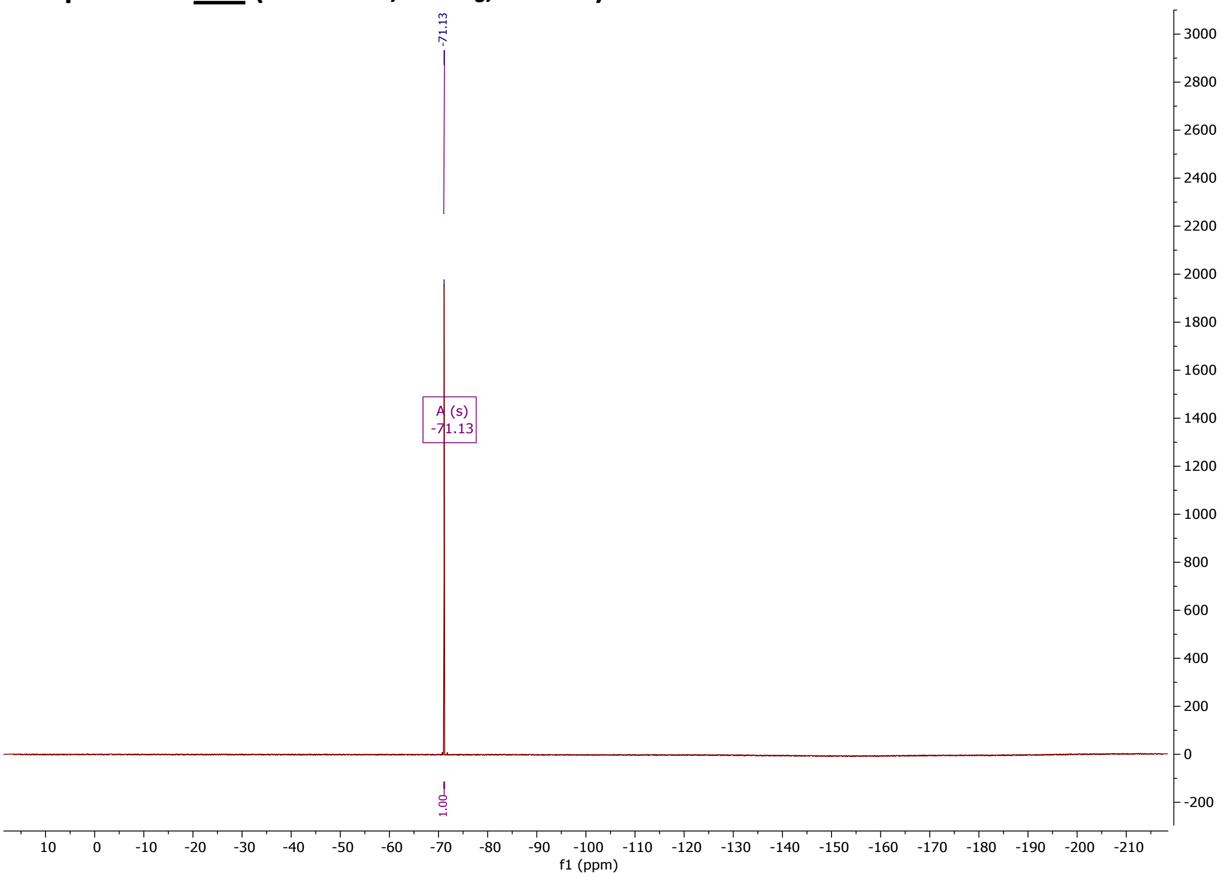

^{13}C spectrum **15k** (75 MHz, Acetone-D$_6$)

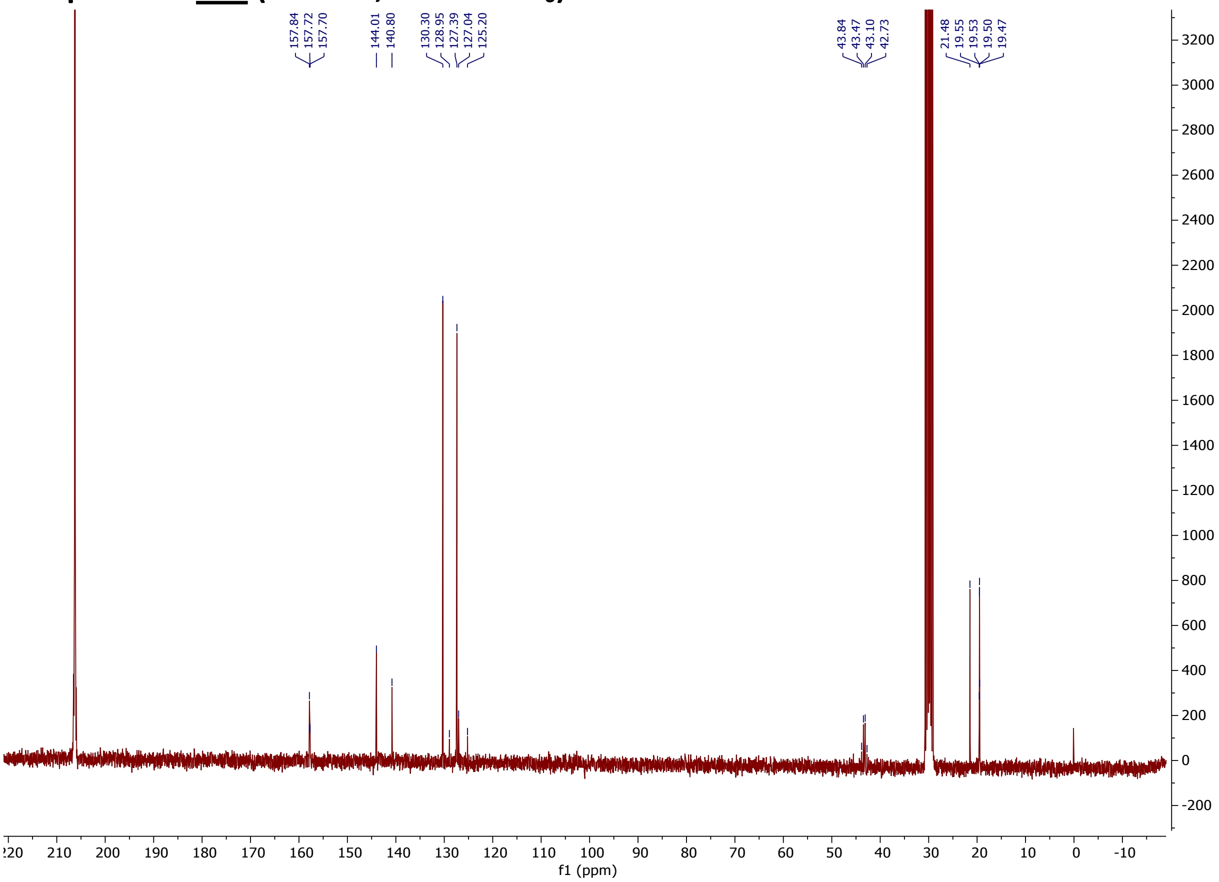

^{1}H spectrum 15m (300 MHz, $CDCl_3$)

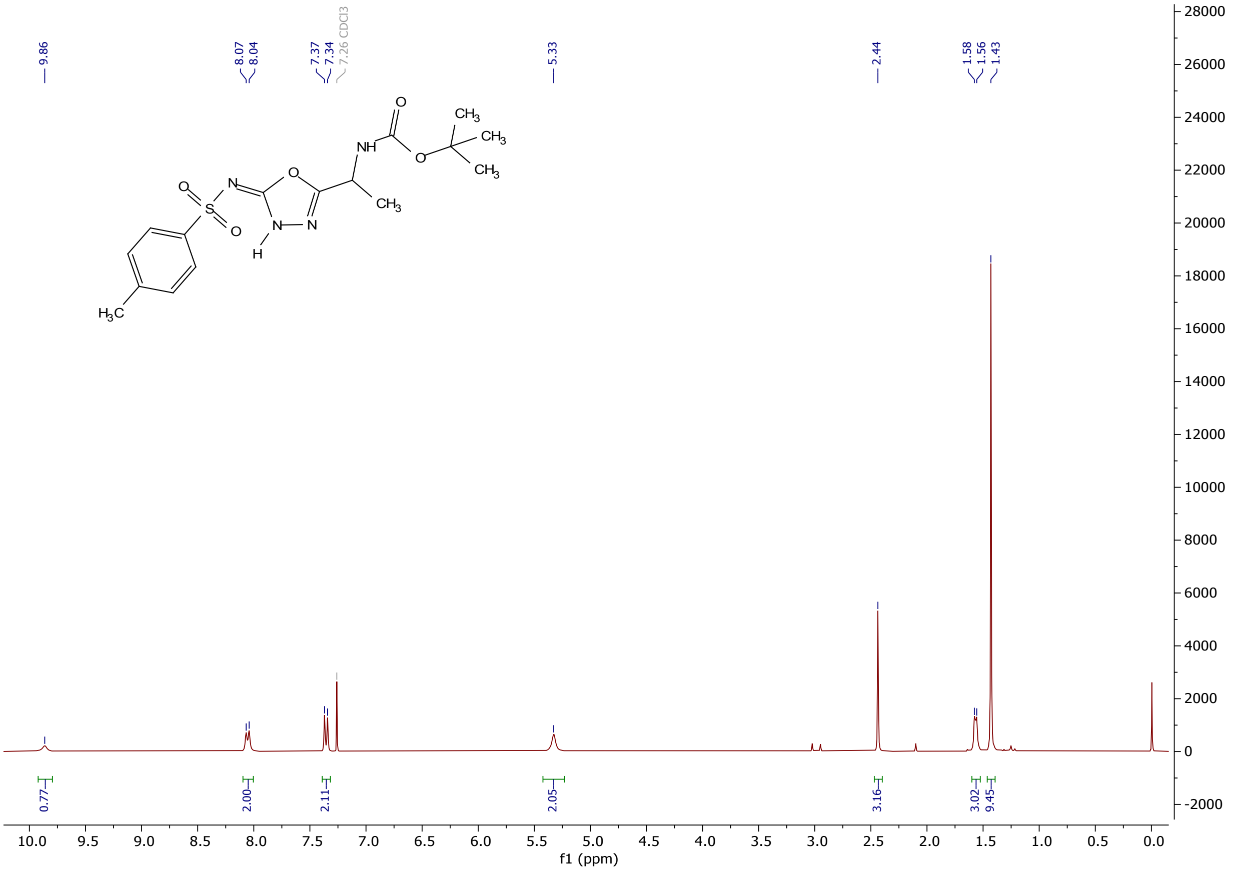

^{13}C spectrum 15m (75 MHz, $CDCl_3$)

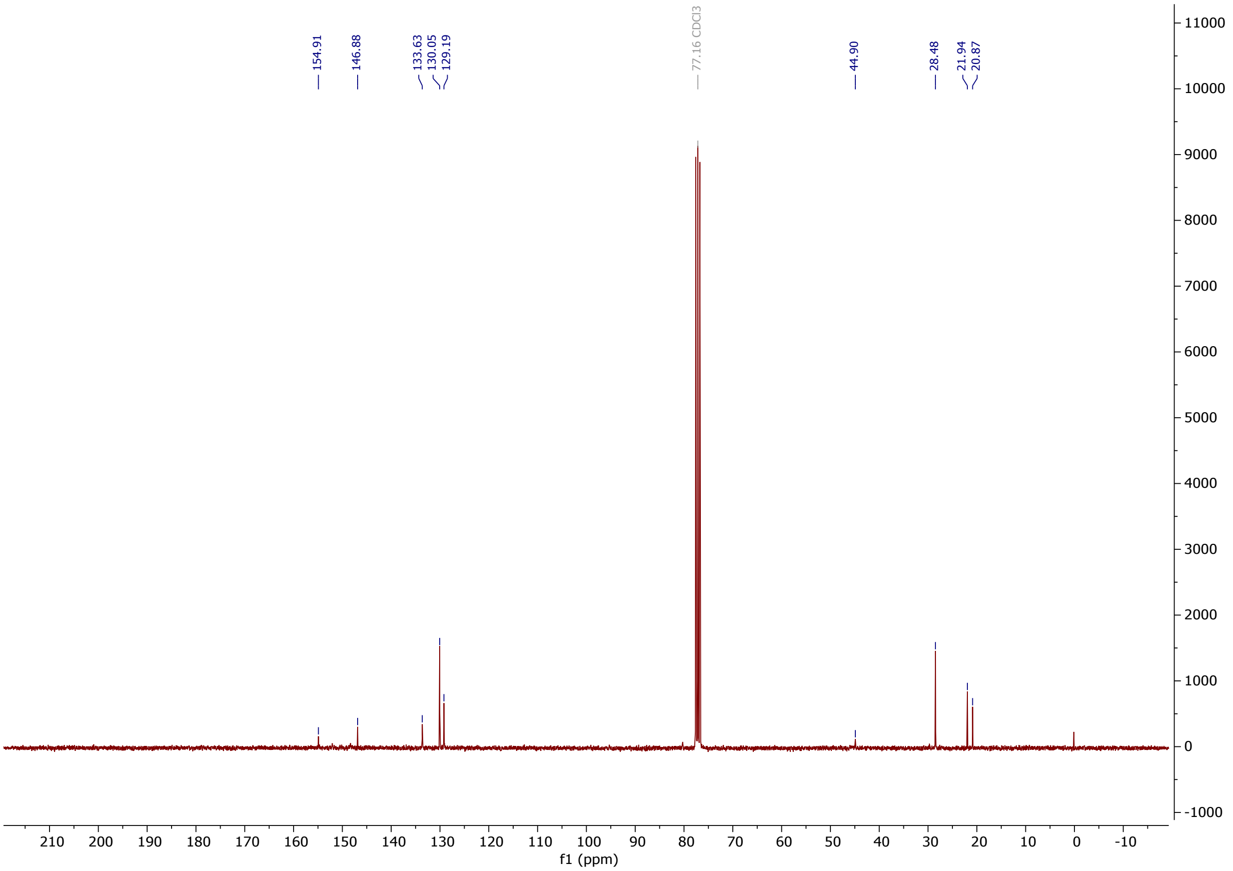

^1H spectrum 15n (300 MHz, $CDCl_3$)

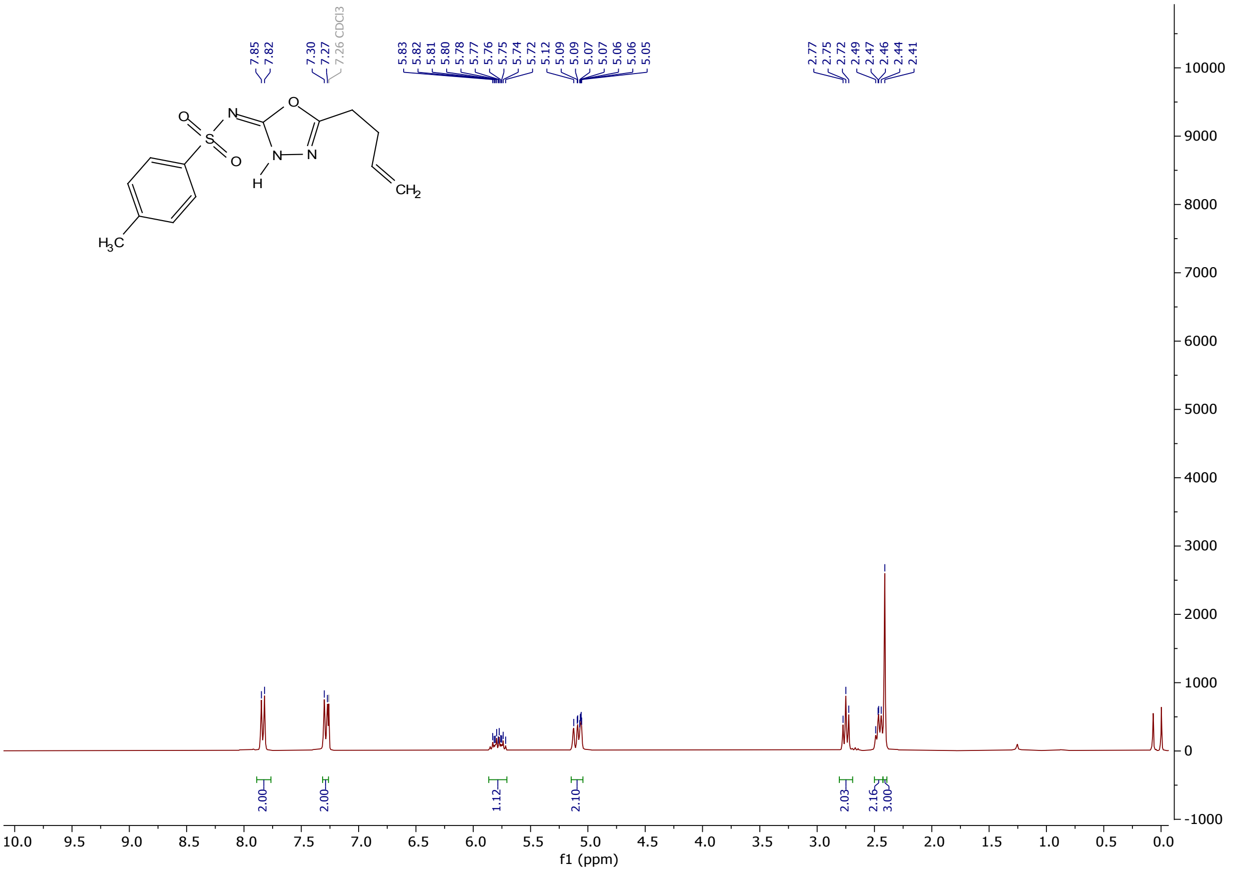

^{13}C spectrum 15n (75 MHz, $CDCl_3$)

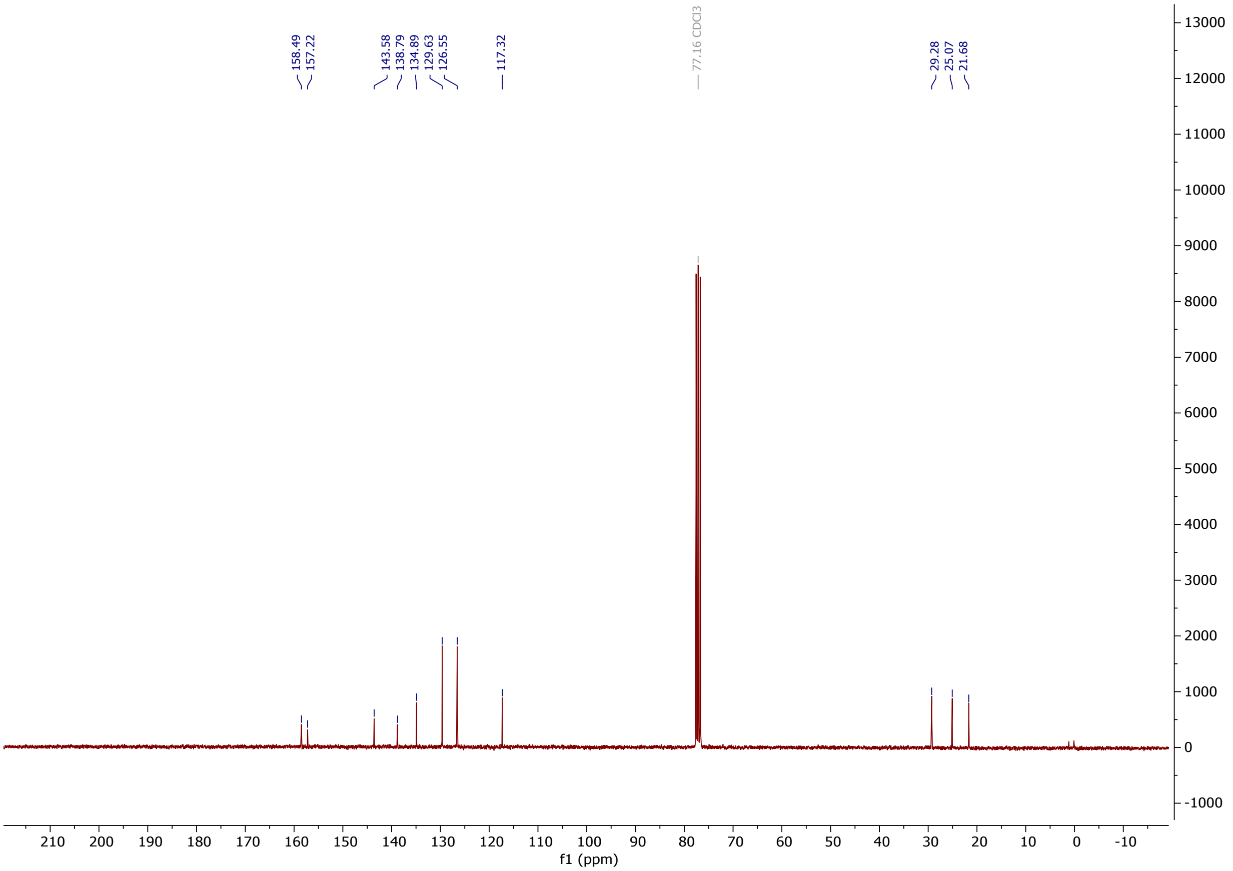

^1H spectrum 15o (300 MHz, $CDCl_3$)

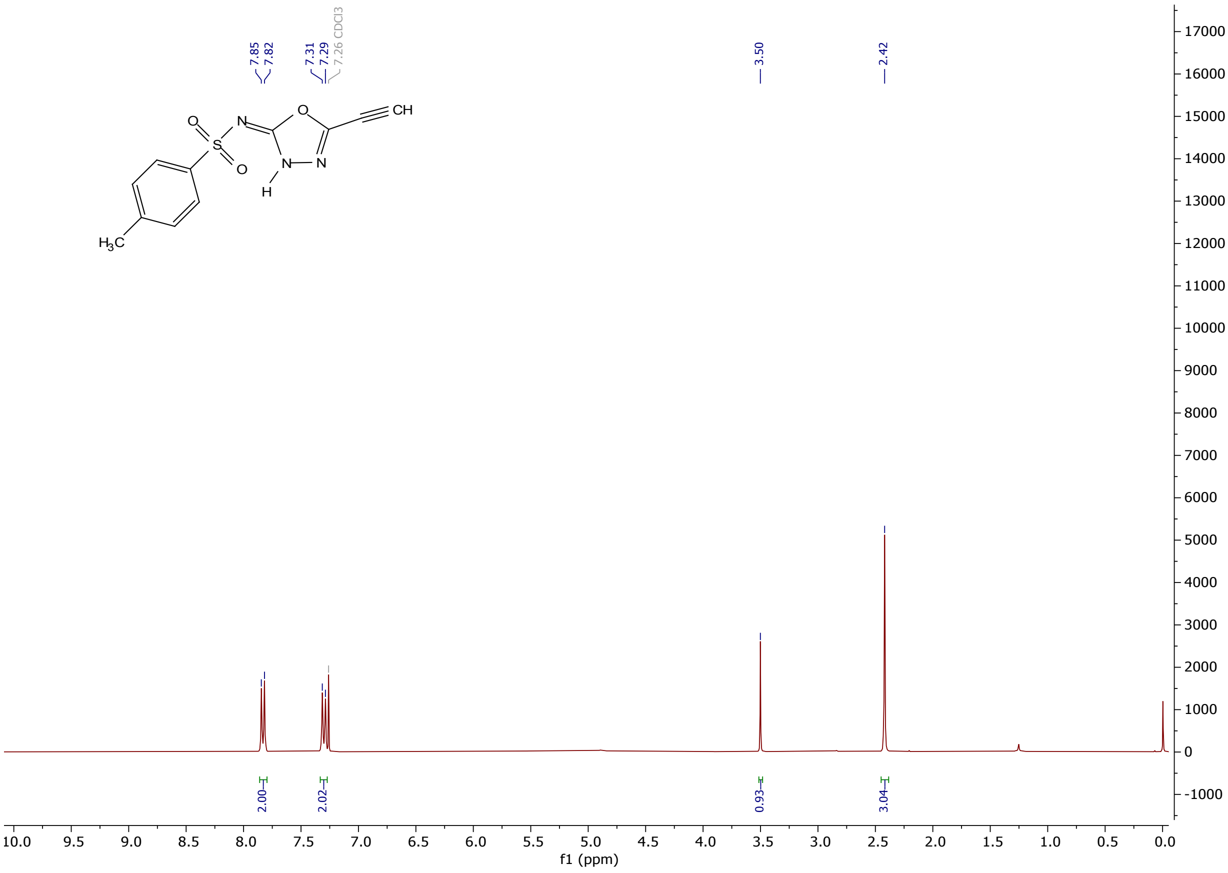

^{13}C spectrum <u>15o</u> (75 MHz, $CDCl_3$)

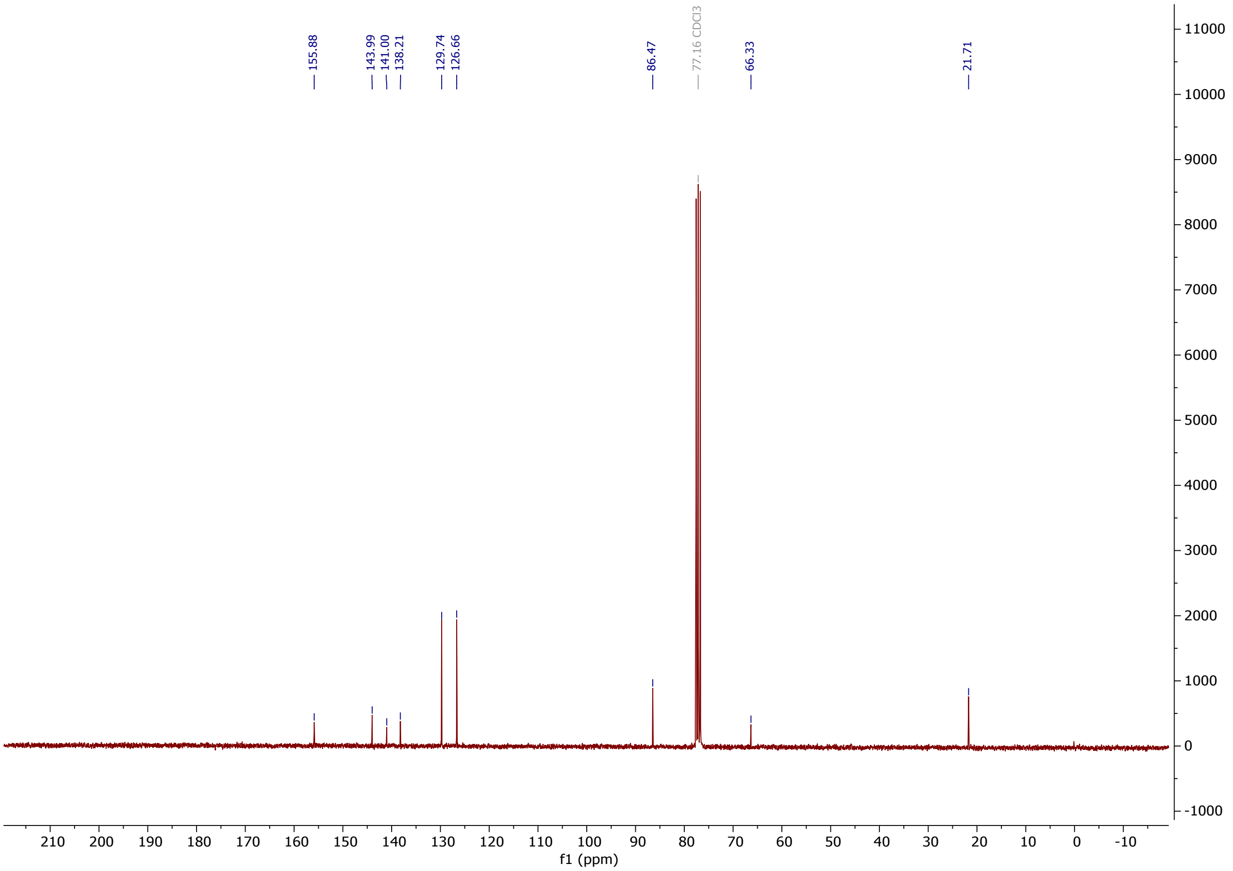

^{1}H spectrum 15p (300 MHz, $CDCl_3$)

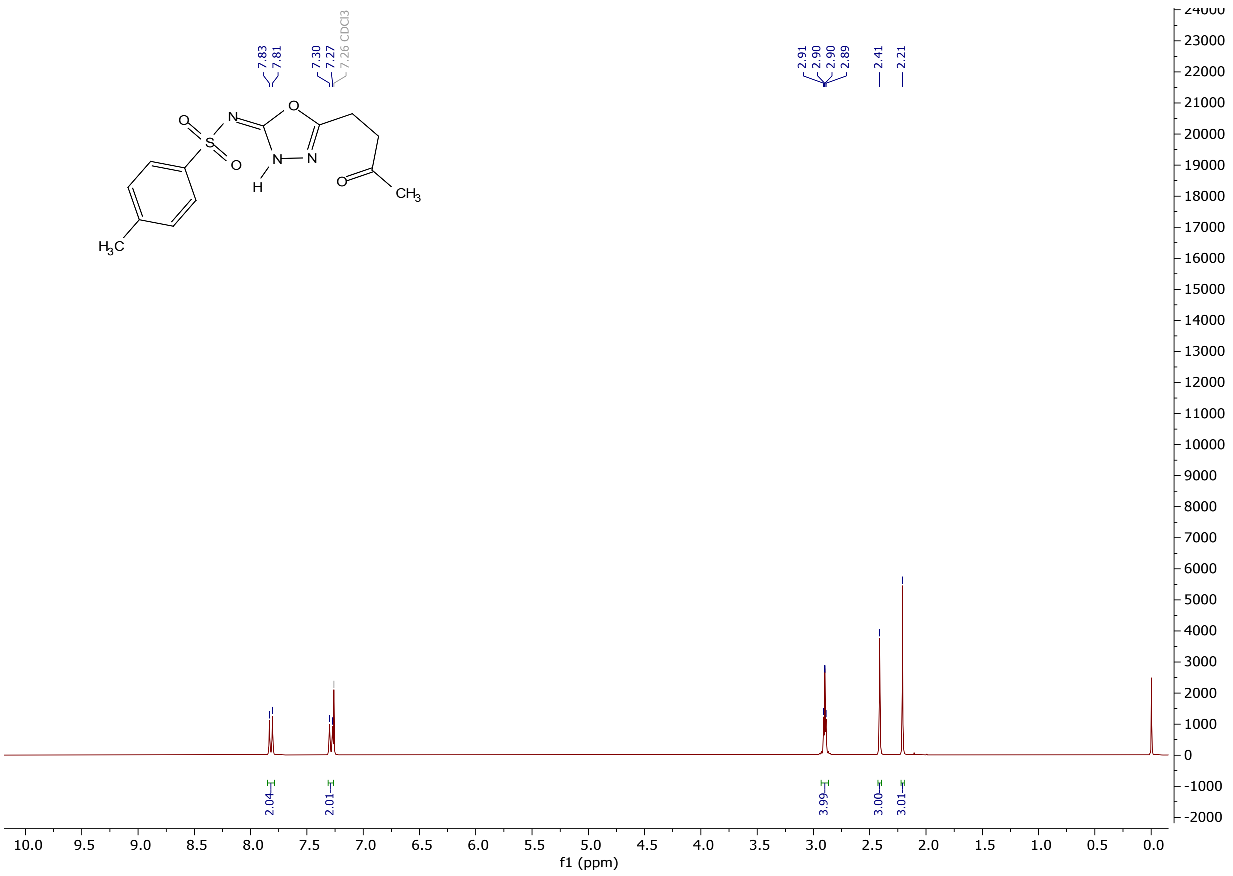

^{13}C spectrum 15p (75 MHz, $CDCl_3$)

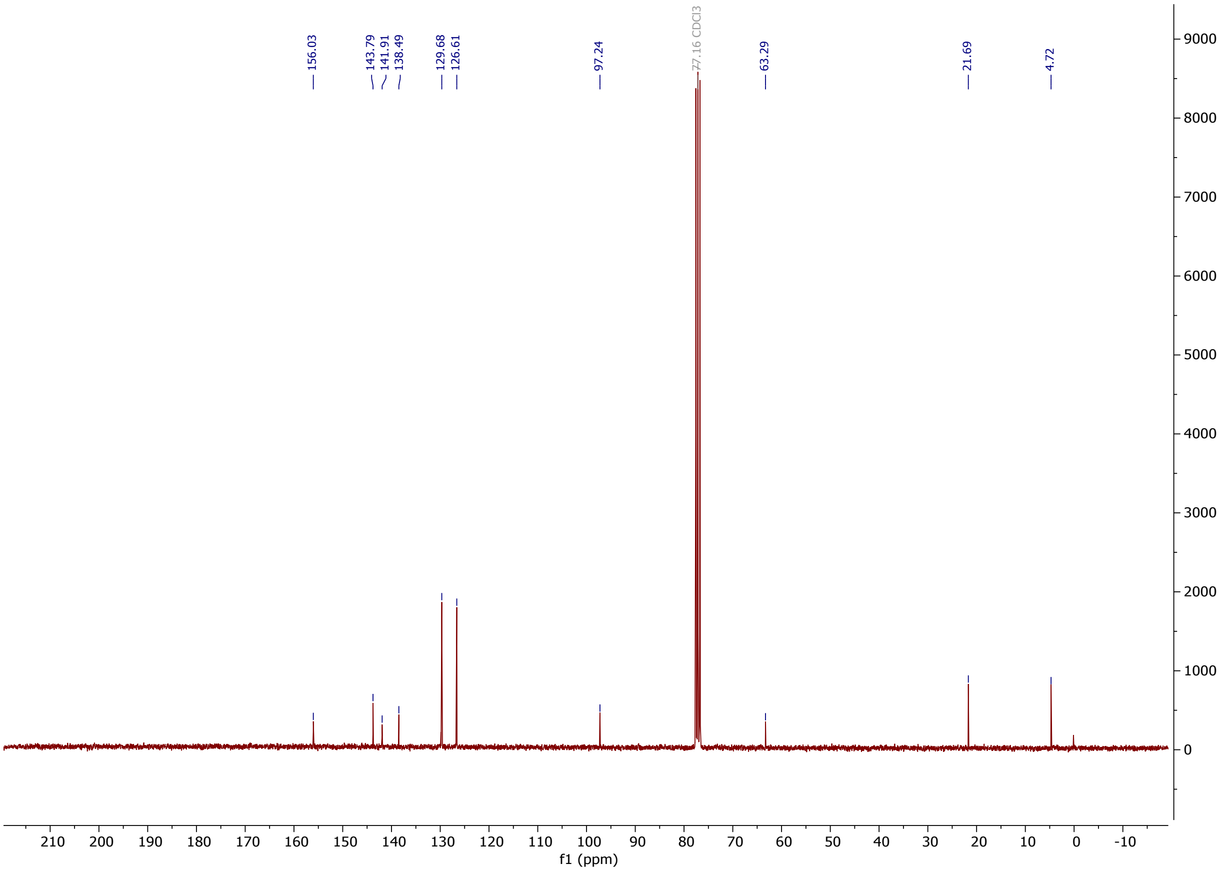

^{1}H spectrum 15q (300 MHz, $CDCl_3$)

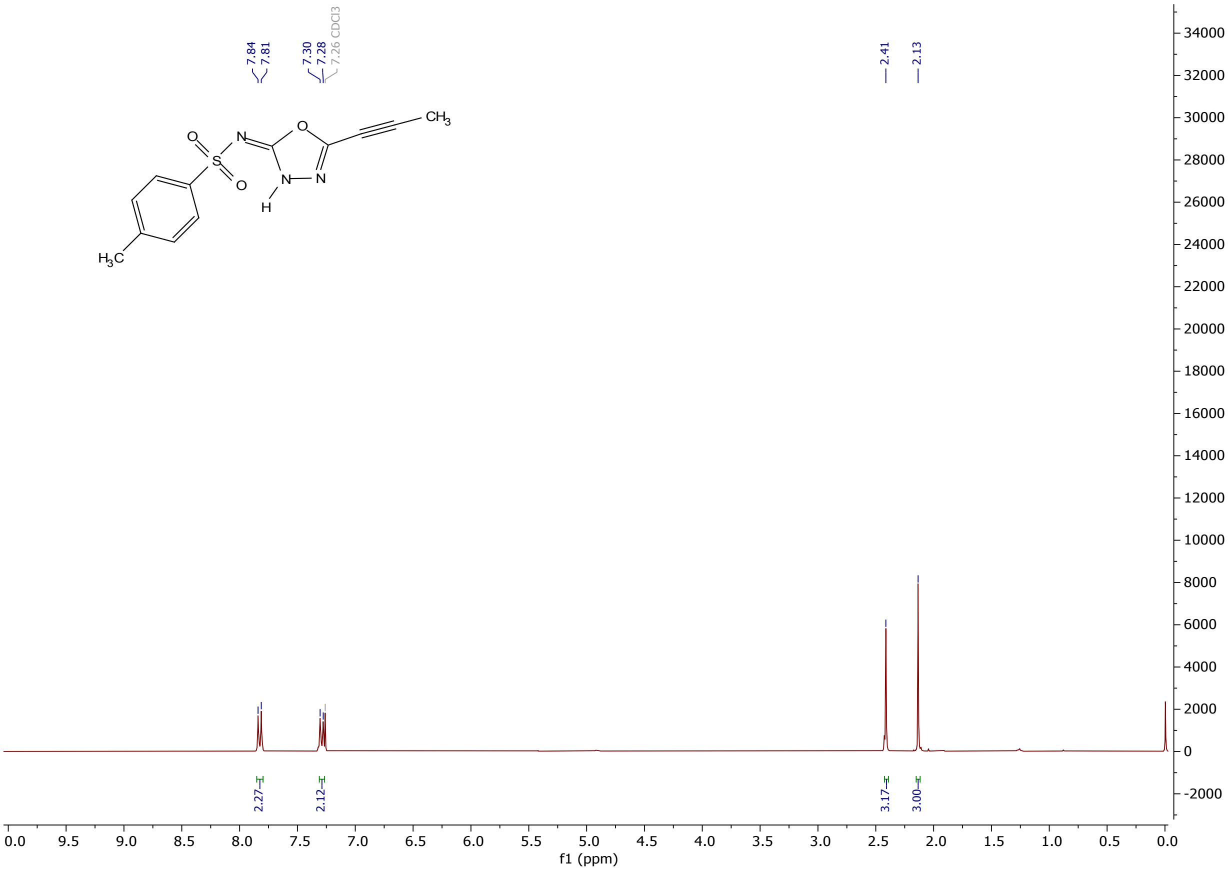

^{13}C spectrum 15q (75 MHz, $CDCl_3$)

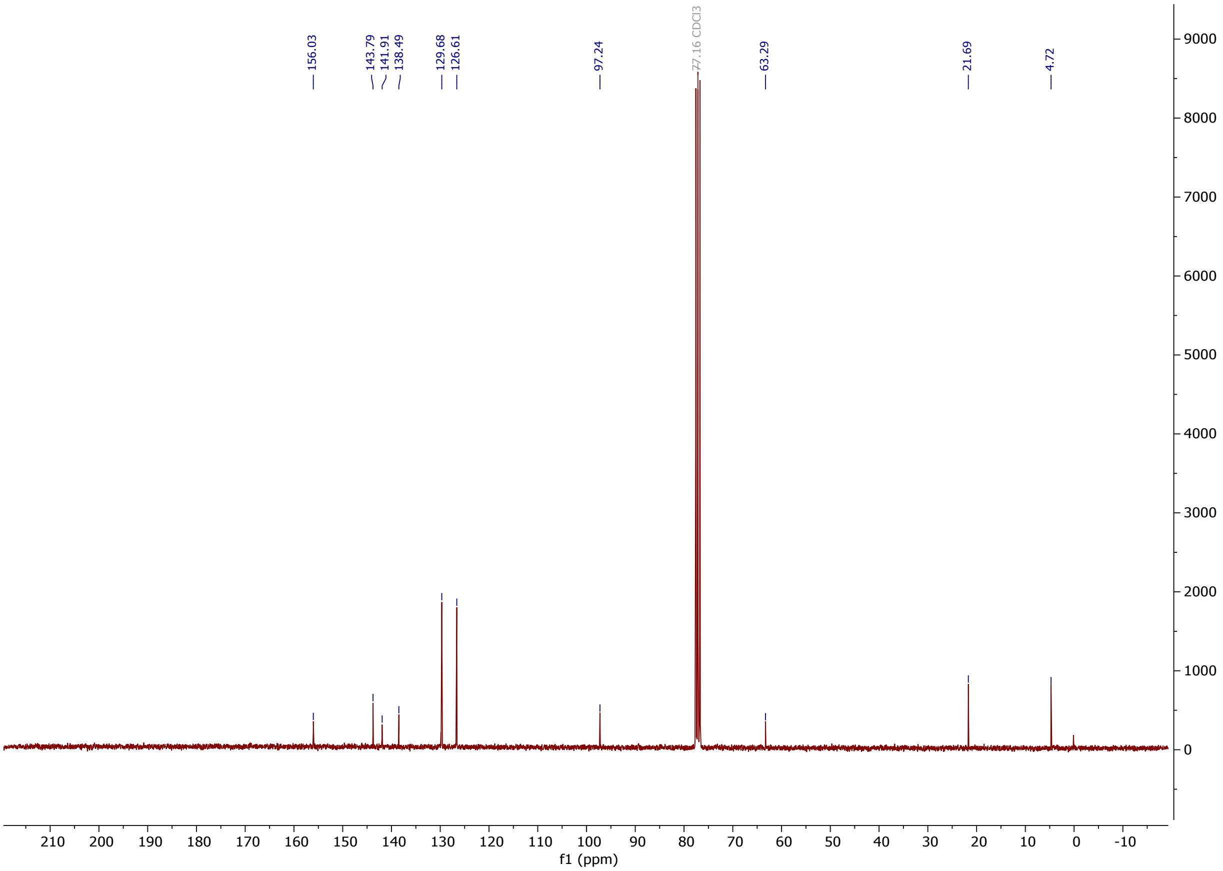

^{1}H spectrum 15r (300 MHz, $CDCl_3$)

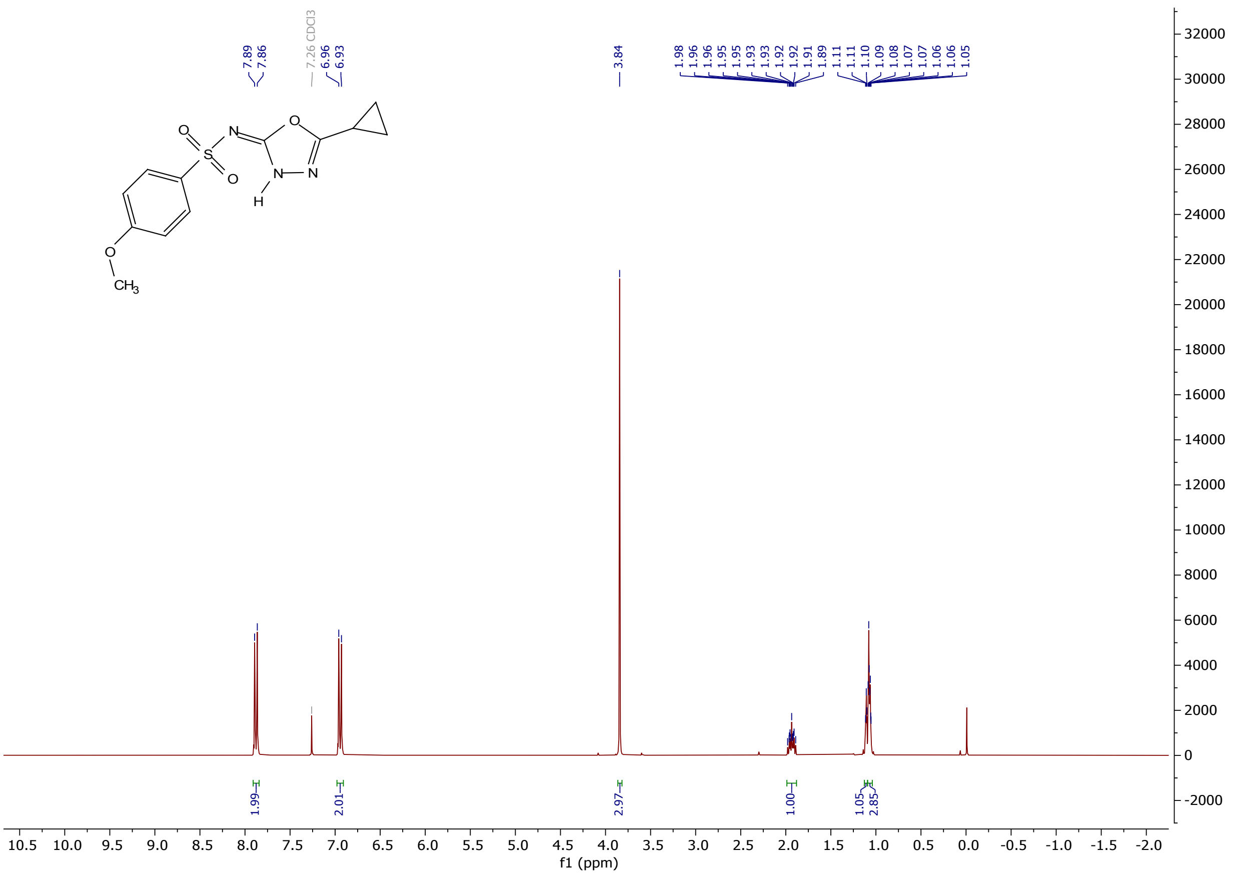

^{13}C spectrum <u>15r</u> (75 MHz, $CDCl_3$)

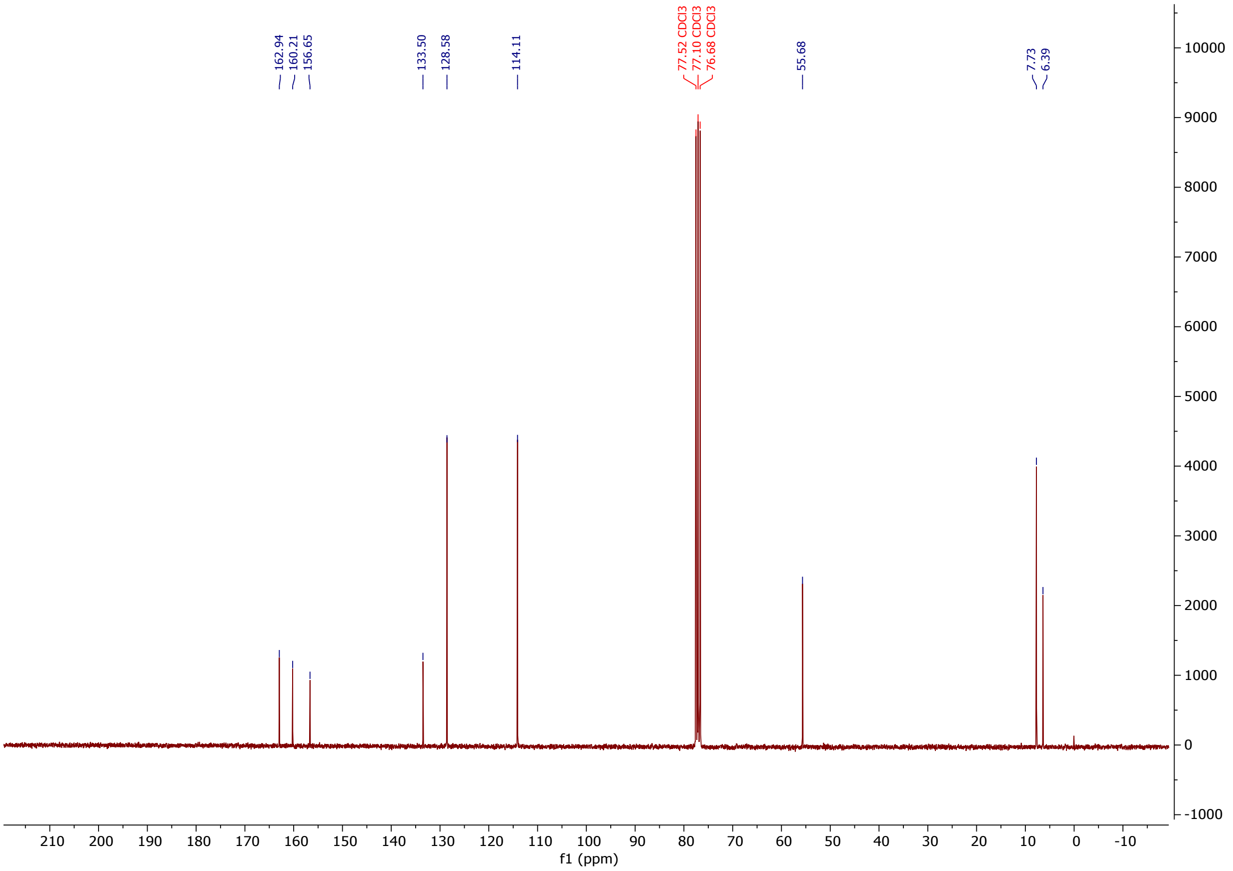

^{1}H spectrum 15s (300 MHz, $CDCl_3$)

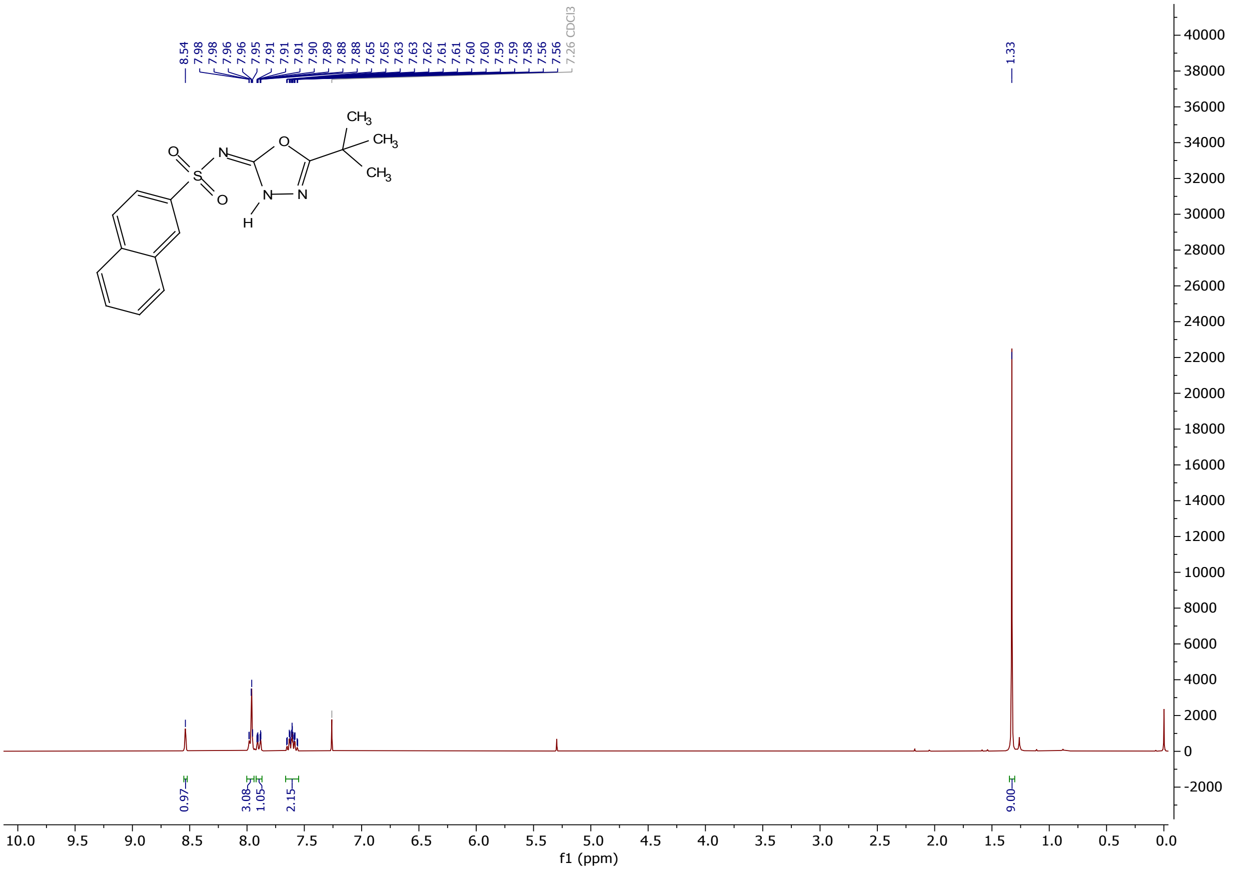

^{13}C spectrum <u>15s</u> (75 MHz, $CDCl_3$)

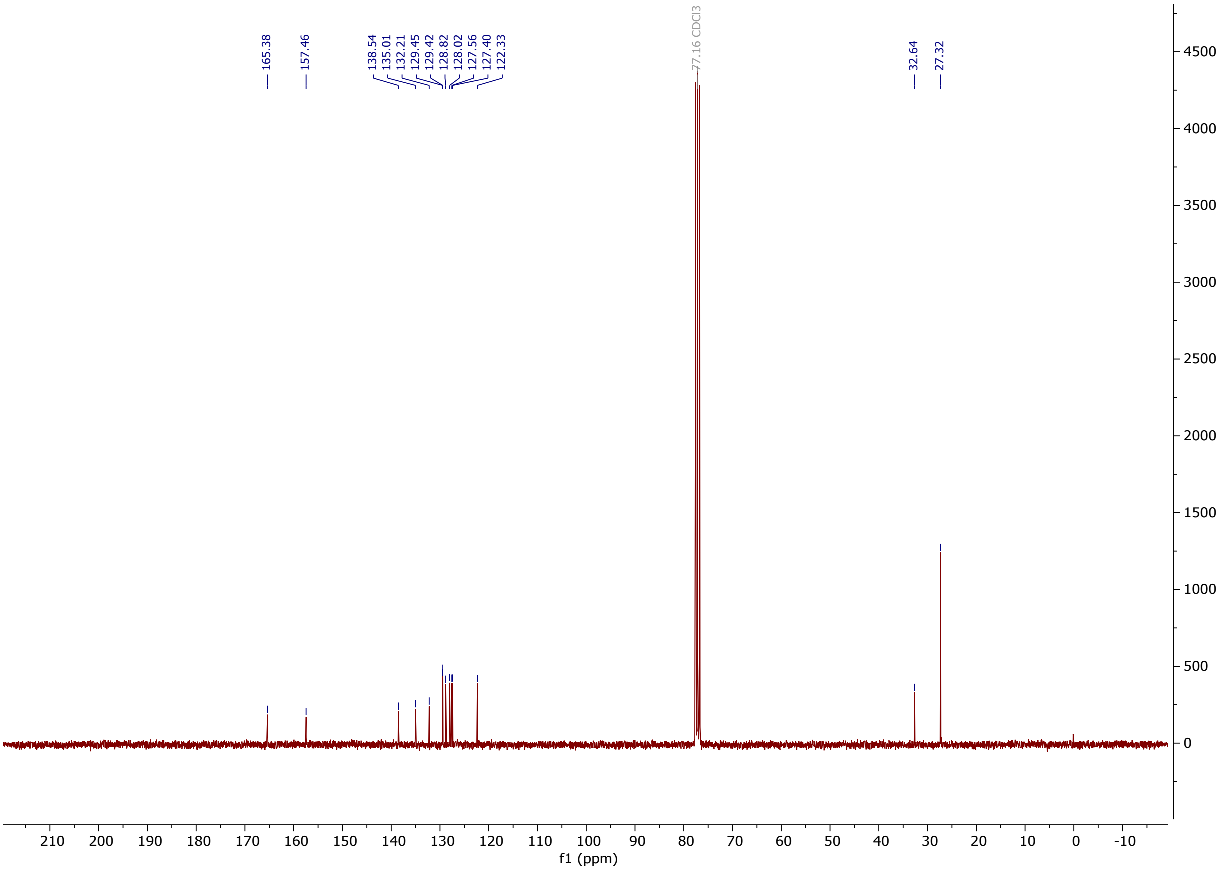

^{1}H spectrum <u>15t</u> (300 MHz, $CDCl_3$)

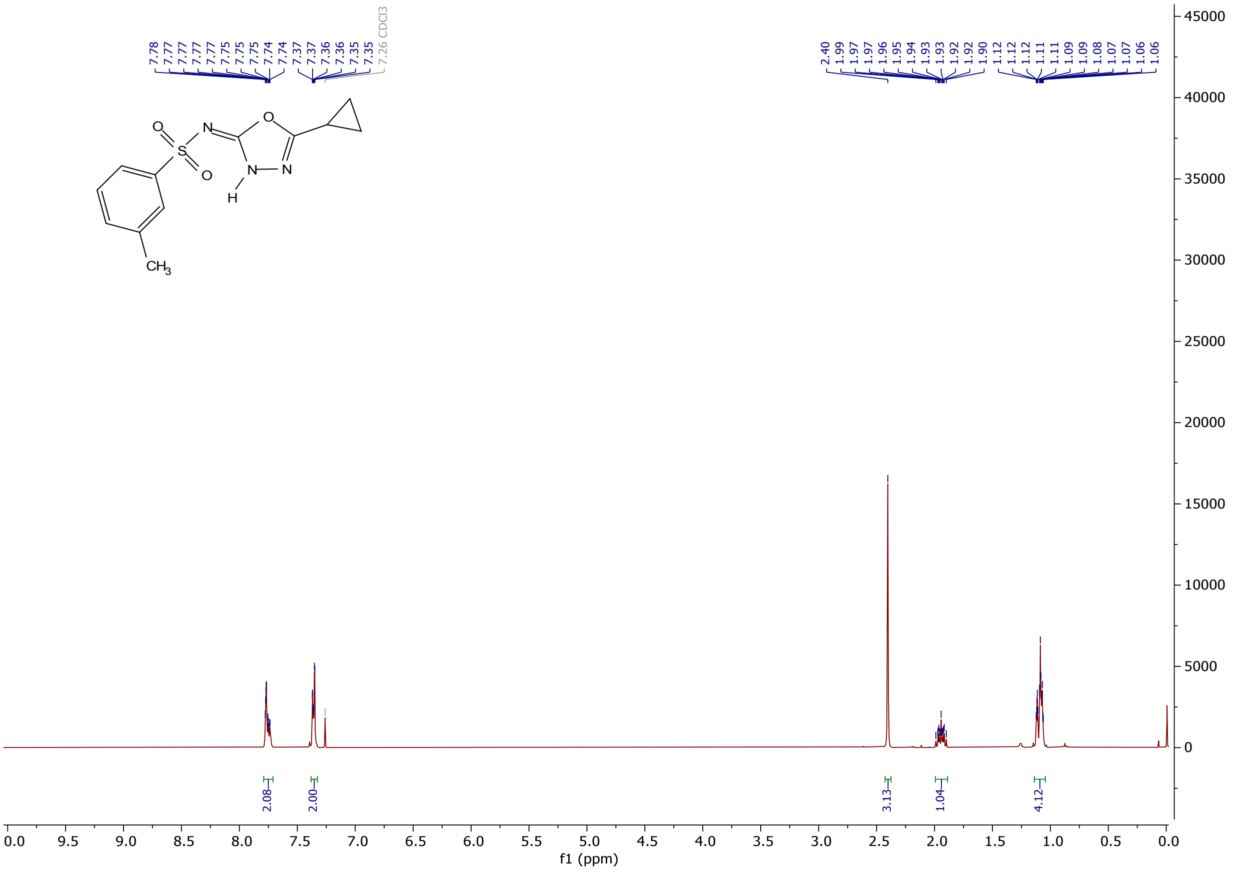

^{13}C spectrum <u>15t</u> (75 MHz, $CDCl_3$)

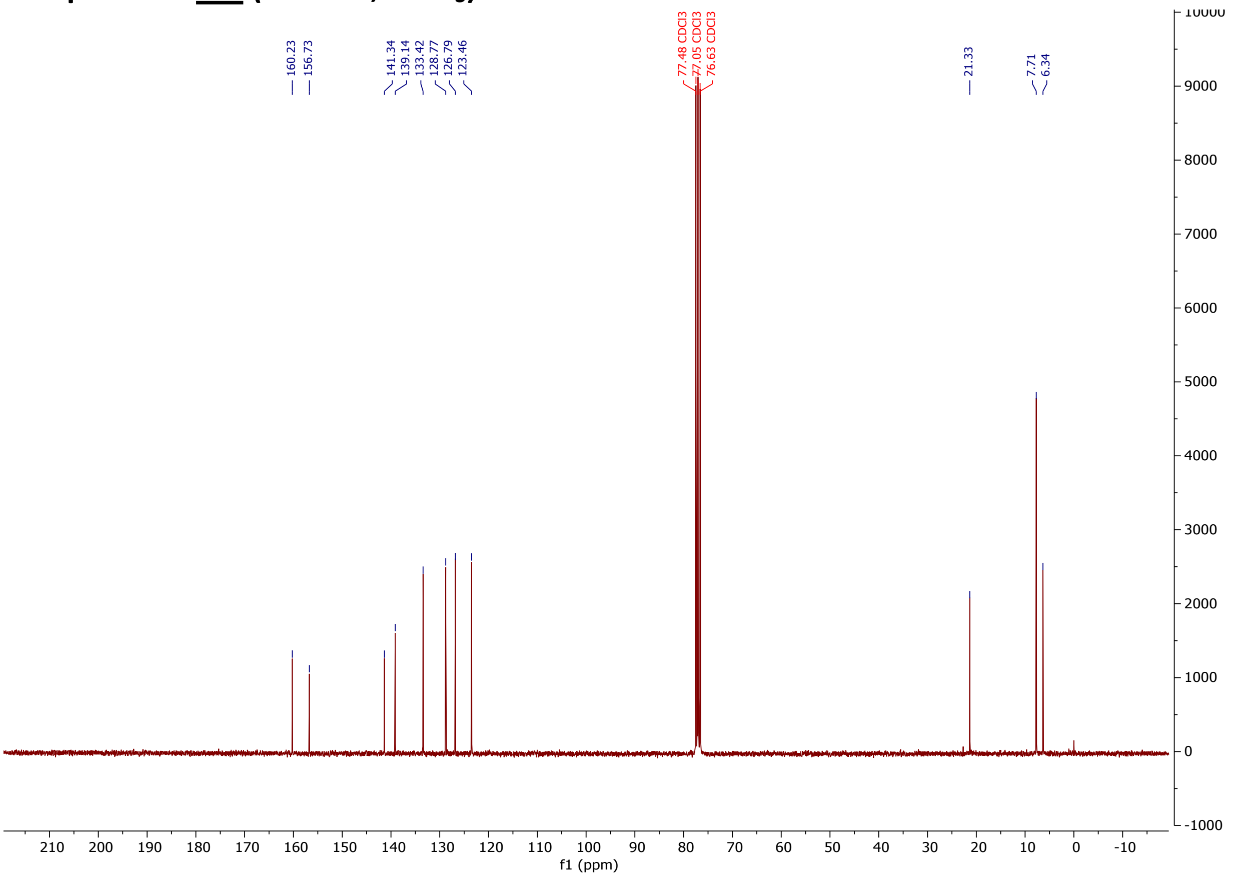

^{1}H spectrum 15u (300 MHz, $CDCl_3$)

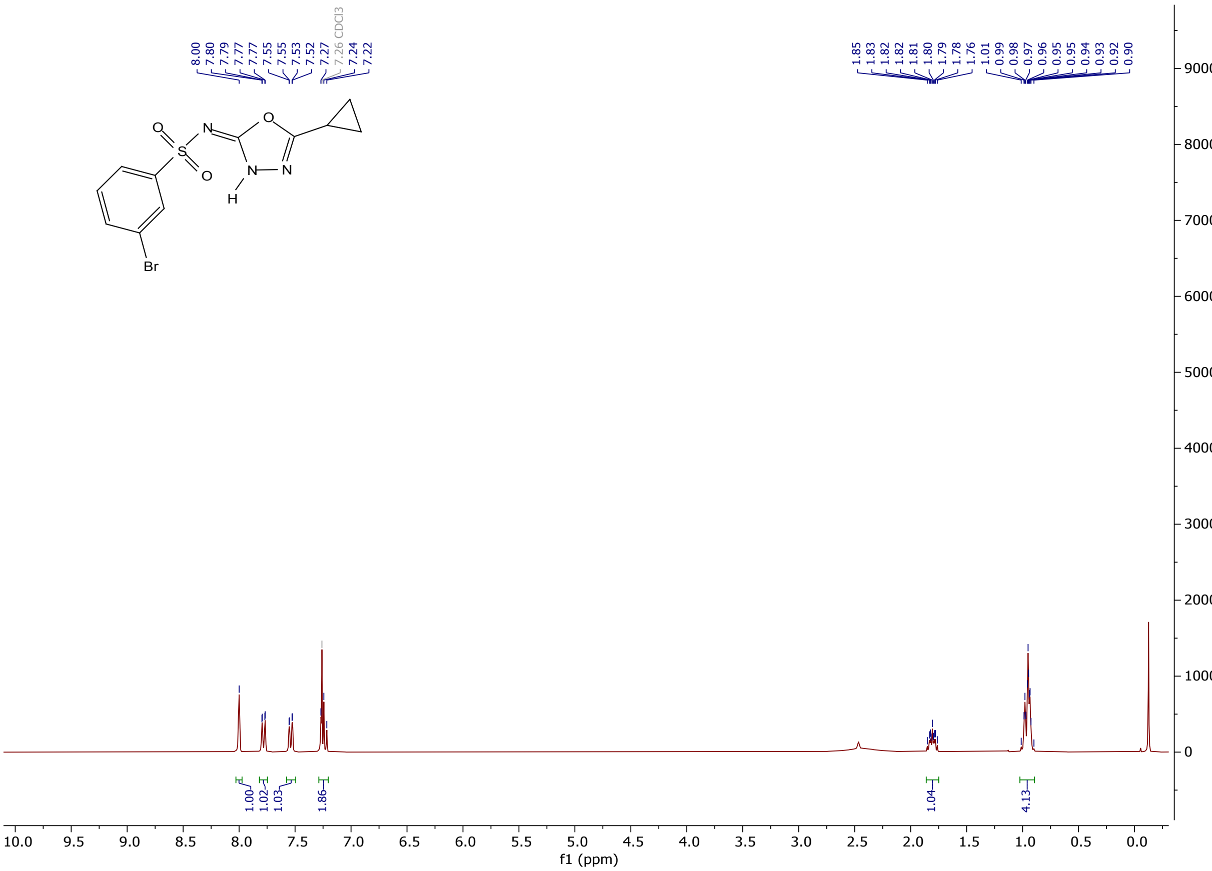

^{13}C spectrum <u>15u</u> (75 MHz, $CDCl_3$)

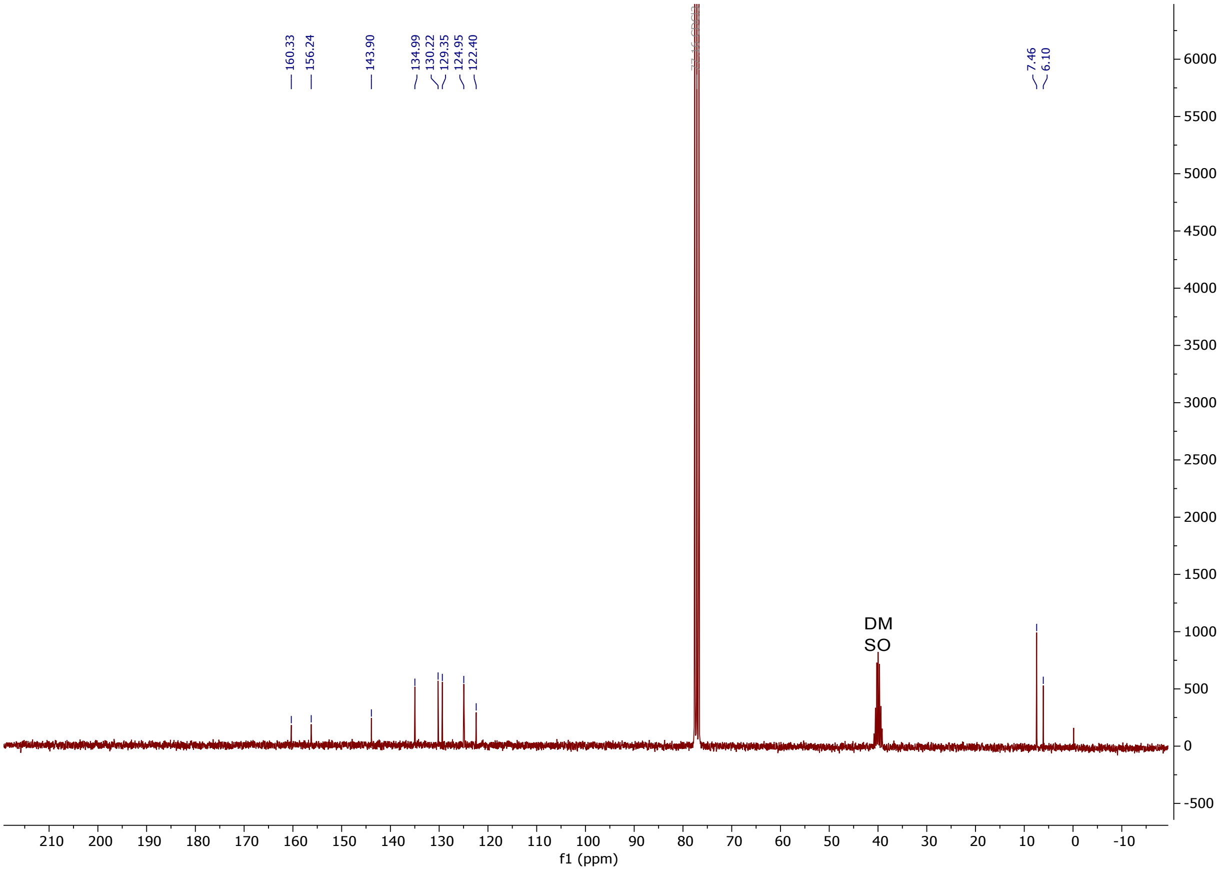

^{1}H spectrum 15v (300 MHz, $CDCl_3$)

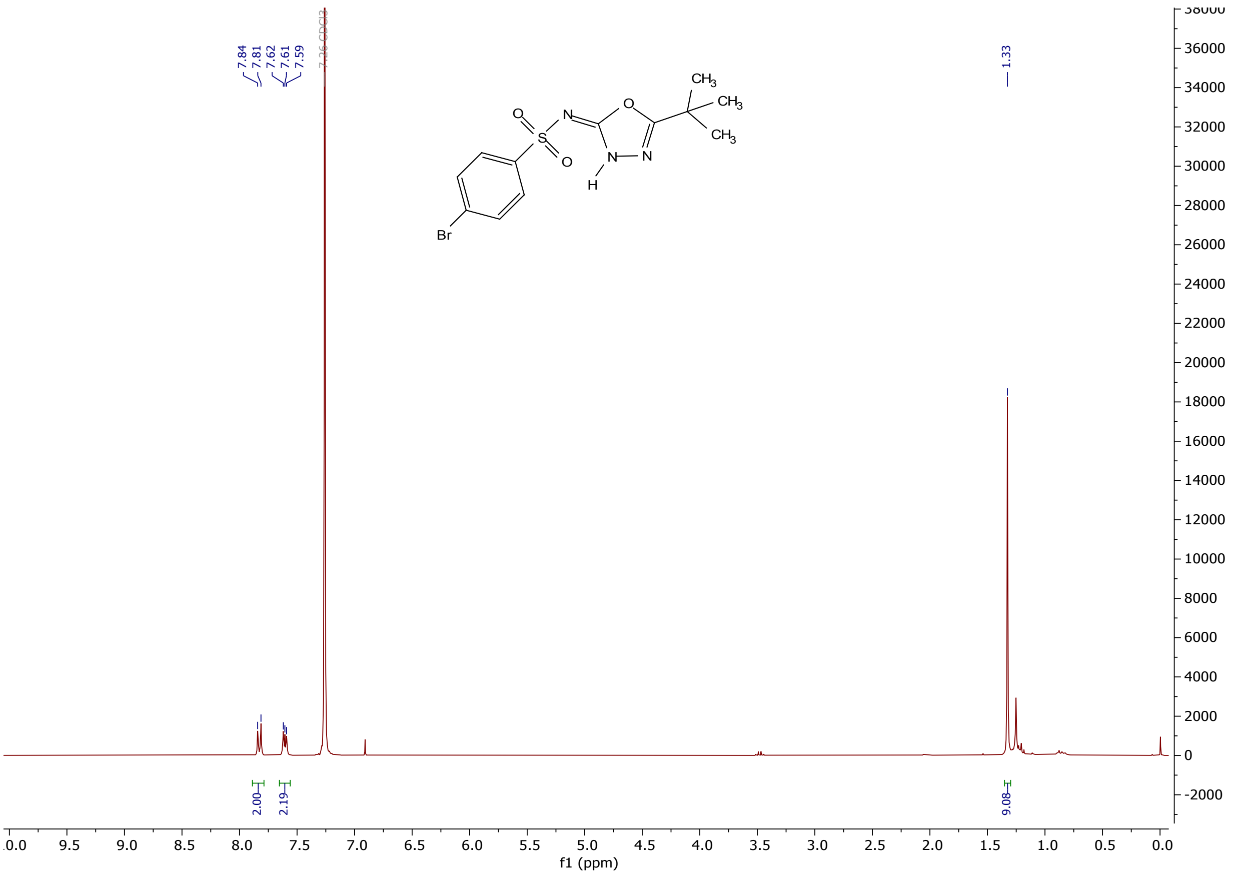

^{13}C spectrum 15v (75 MHz, $CDCl_3$)

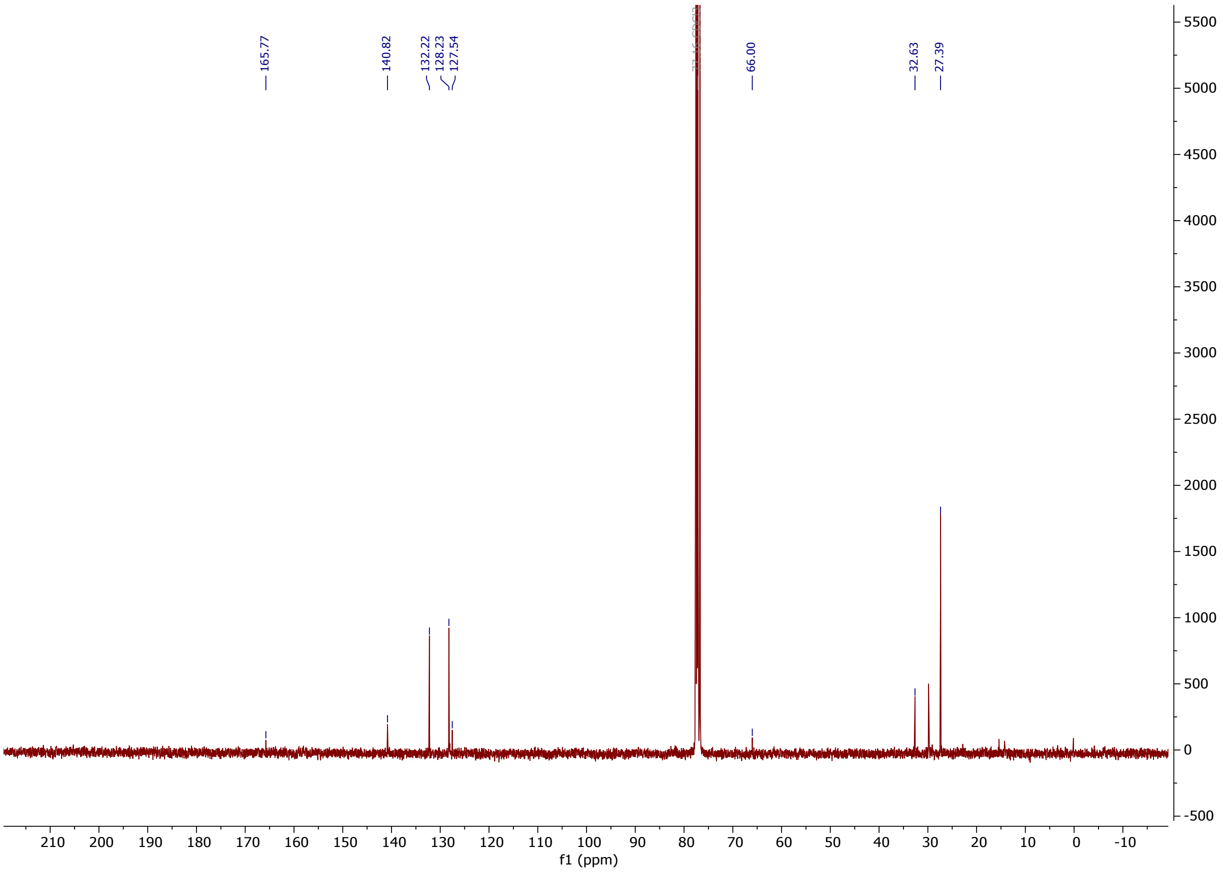

^1H spectrum 15w (300 MHz, $CDCl_3$)

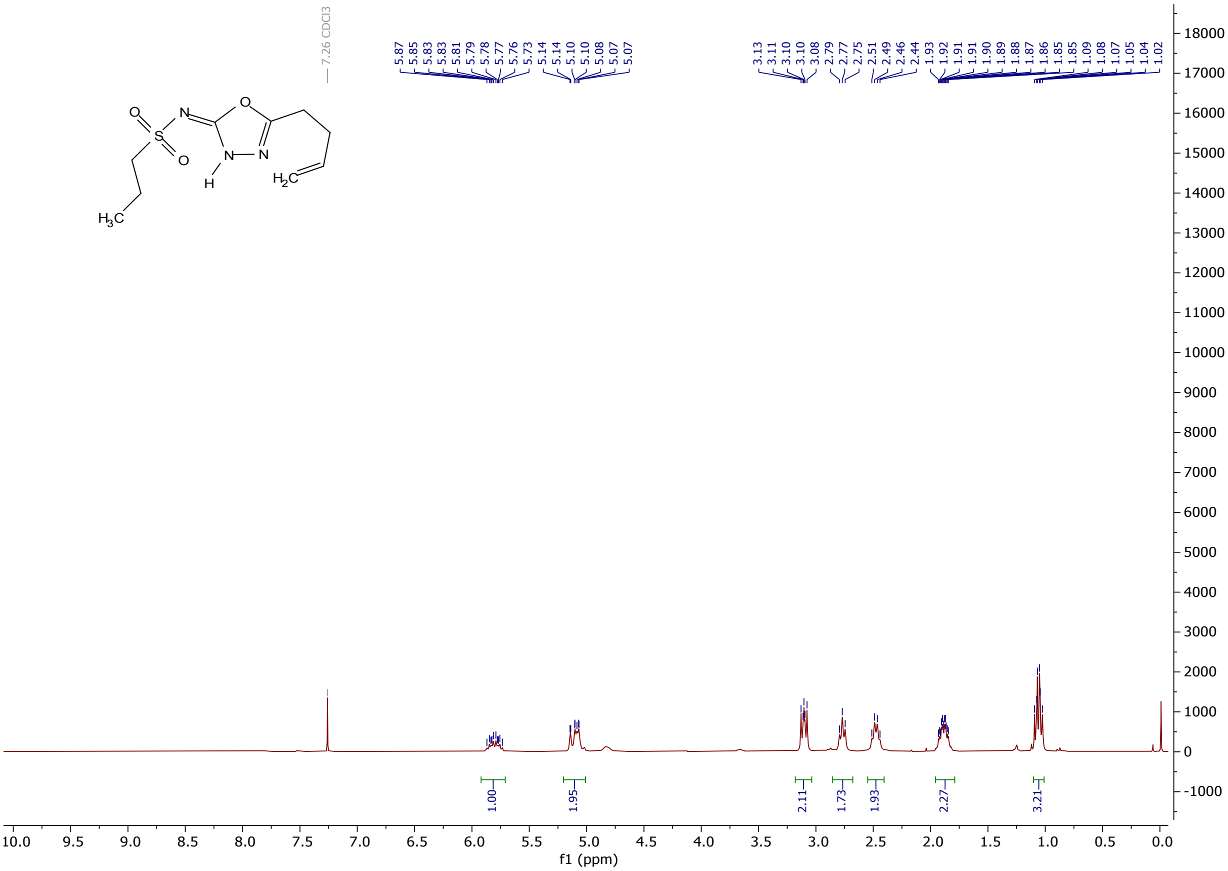

^{13}C spectrum 15w (75 MHz, $CDCl_3$)

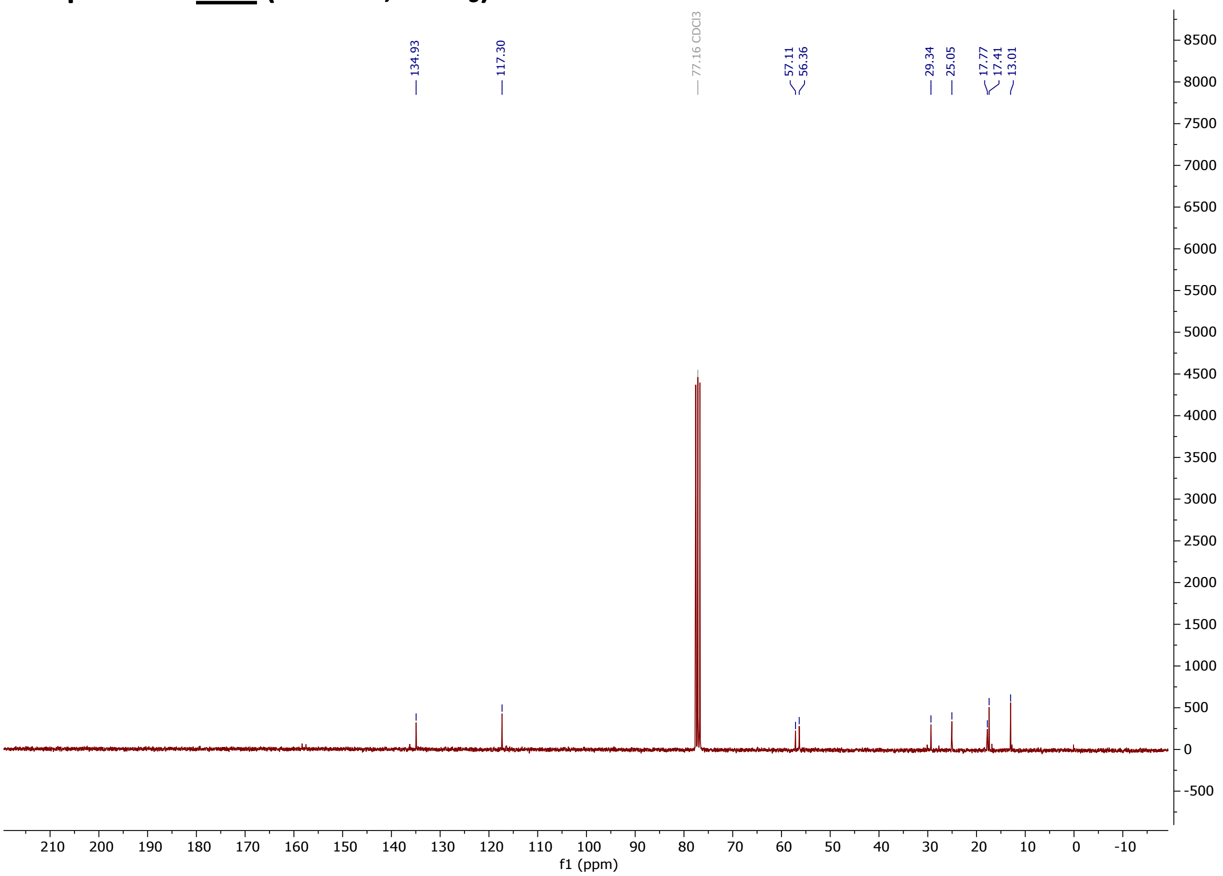

^{1}H spectrum 15x (300 MHz, $CDCl_3$)

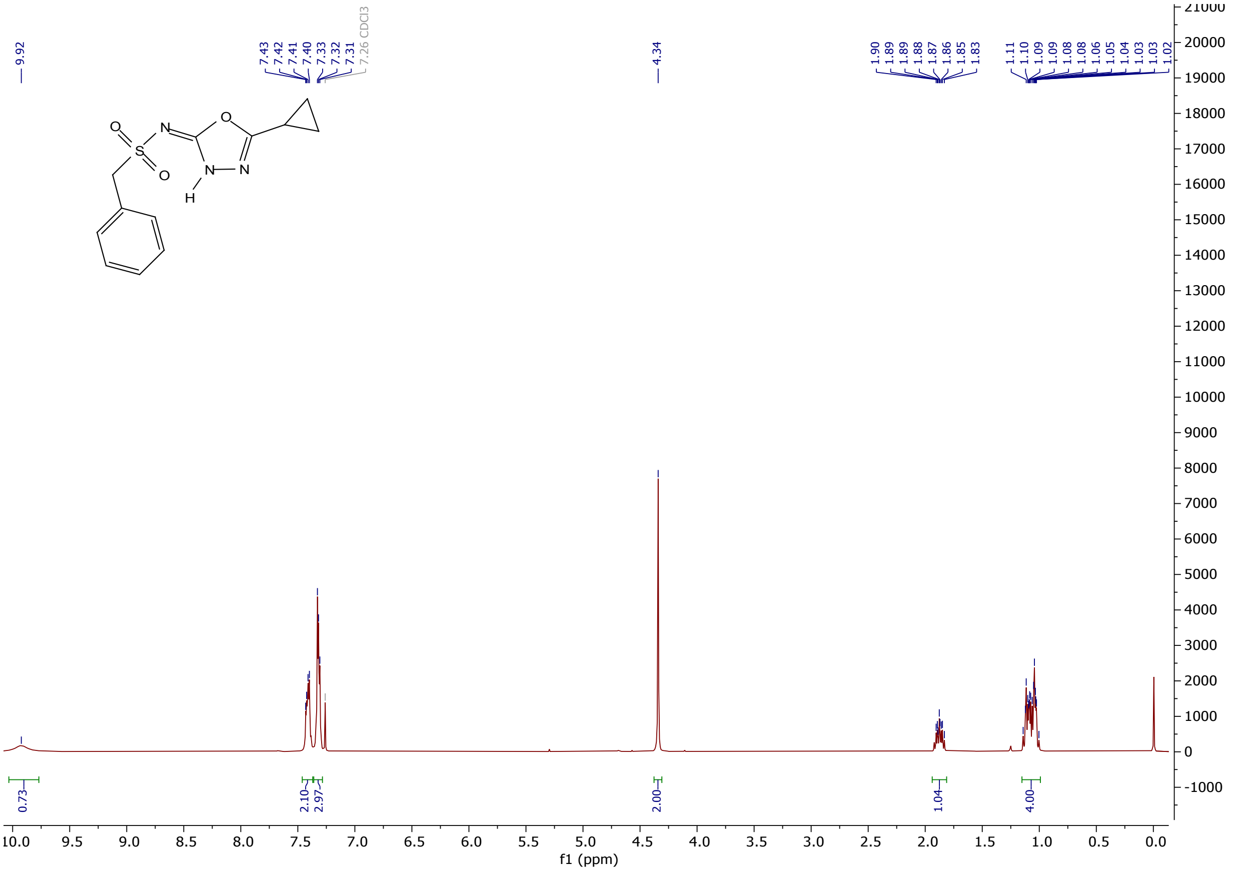

^{13}C spectrum 15x (75 MHz, $CDCl_3$)

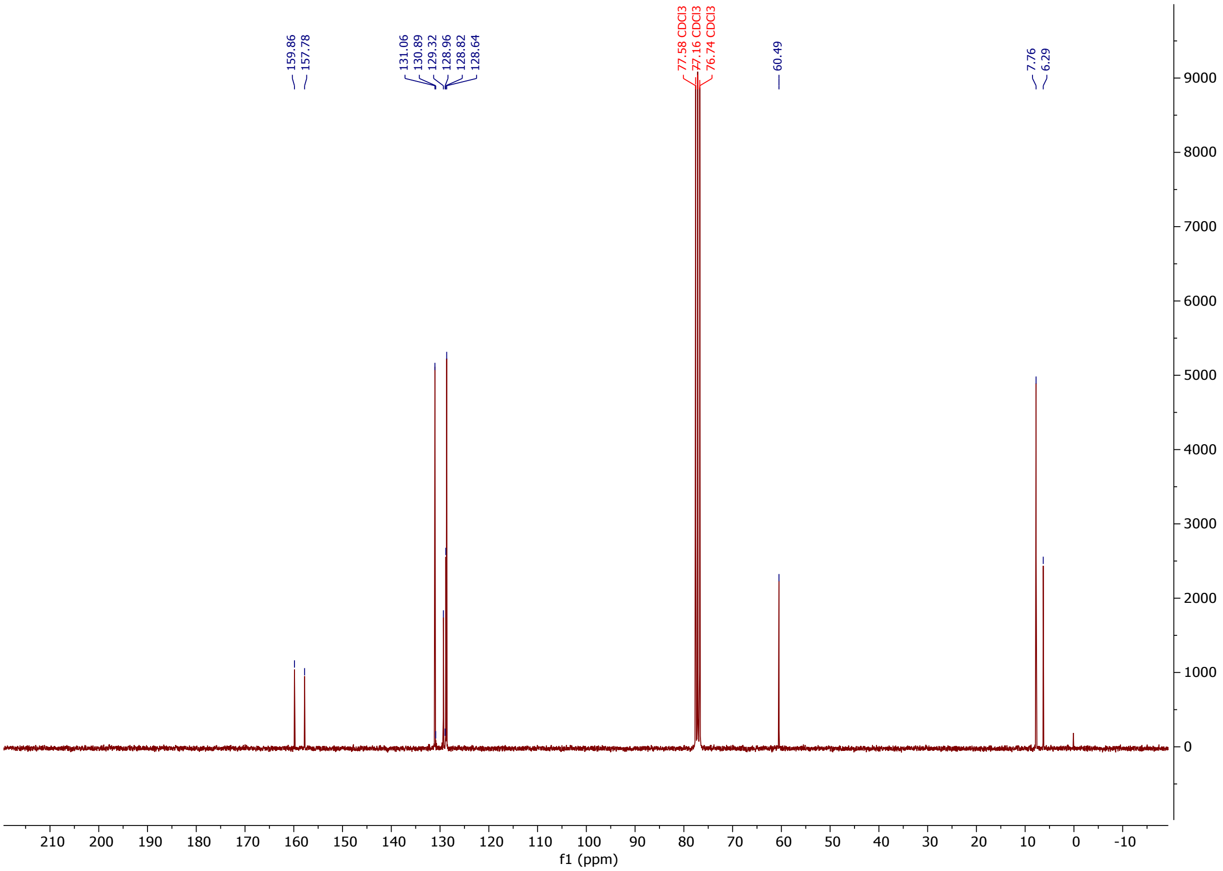

^{1}H spectrum 15y (300 MHz, $CDCl_3$)

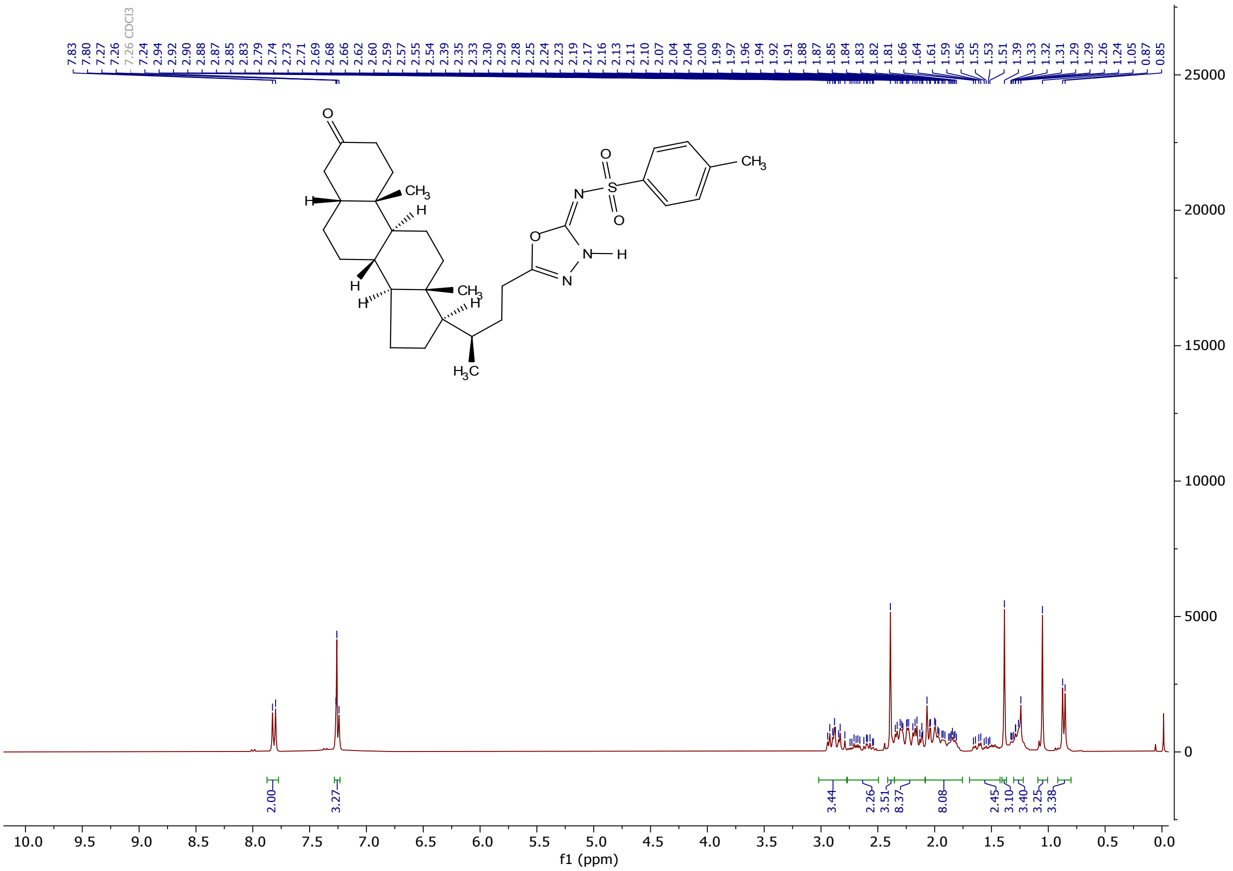

^{13}C spectrum 15y (75 MHz, $CDCl_3$)

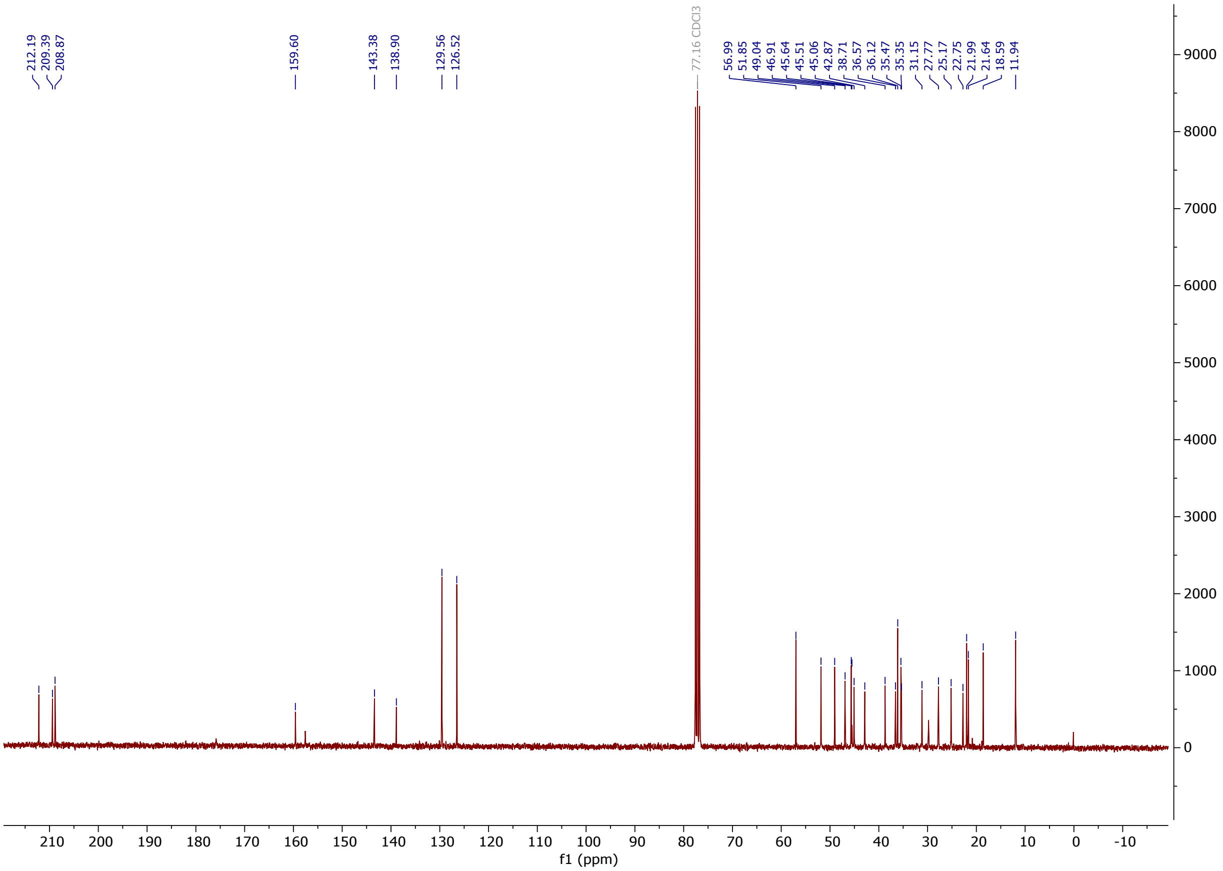

Supplement: Supplementary file 1 — ol3c00959_si_001.pdf [file ol3c00959_si_001.pdf]
